# Polymer‐Grafted Cu(II) Metal–Organic Polyhedra as Scalable, Melt‐Processable Porous Materials

**DOI:** 10.1002/advs.77006

**Published:** 2026-09-07

**Authors:** Alba Cortés‐Martínez, Francisco Sánchez‐Férez, José A. Suárez del Pino, Sergio Royuela, Félix Zamora, Daniel Maspoch, Arnau Carné‐Sánchez

**Affiliations:** ^1^ Catalan Institute of Nanoscience and Nanotechnology (ICN2) CSIC and The Barcelona Institute of Science and Technology Bellaterra Barcelona Spain; ^2^ Departament de Química Facultat de Ciències Universitat Autònoma de Barcelona (UAB) Cerdanyola del Vallès Barcelona Spain; ^3^ Departamento De Química Inorgánica and Condensed Matter Physics Institute (IFIMAC) Universidad Autónoma de Madrid Ciudad Universitaria de Cantoblanco Madrid Spain; ^4^ ICREA Barcelona Spain

**Keywords:** cages, meltable, metal–organic polyhedral (MOP), porosity, porous liquid

## Abstract

The development of porous materials that combine low‐temperature meltability with high gas uptake remains a major challenge. Herein, we report Cu‐BCN‐5, a polymer‐grafted Cu(II) metal–organic polyhedron (MOP), as a scalable melt‐processable material with intrinsic porosity. This material melts at 44°C while preserving cage integrity and accessible porosity. By overcoming diffusion limitations imposed by the polymer corona during sorption measurements, the material reaches a CO_2_ uptake of 0.68 mmol g^−1^ at 298 K and 1 bar, the highest value reported for melt‐processable porous materials under these conditions. Furthermore, the molten state enables fabrication of dense, grain‐boundary‐free macroscopic objects that retain high adsorption performance after shaping. These results establish Cu(II)‐based MOPs as a scalable platform for melt‐processable porous materials.

## Graphical abstract

The use of a direct synthetic route to obtain polymer‐grafted Cu(II)‐based metal–organic polyhedra gives rise to a low‐cost and scalable cage. This material combines low‐temperature melting, achieving phase changes while preserving cage integrity, and high CO_2_ uptake exceeding 0.6 mmol g^−1^. Moreover, the melting enables its processability for the formation of dense macroscopic objects that retain cage structure and porosity.

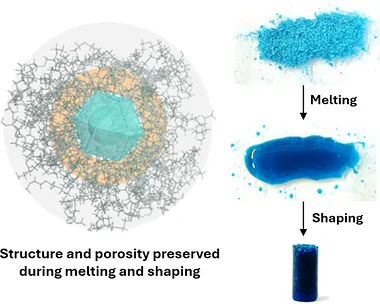

## 1 Introduction

Permanent porosity is typically exhibited by extended crystalline materials such as metal–organic frameworks (MOFs). In these frameworks, pore formation arises from an ordered lattice that enables precise tuning of the pore structure and its associated properties [1, 2, 3, 4, 5]. However, the high stabilization energy of these extended architectures, together with the restricted molecular mobility of their building units, generally suppresses thermal transitions such as melting or glass formation. Consequently, most MOFs decompose before reaching a liquid state, severely limiting their ability to be processed into functional macroscopic objects using conventional melt‐processing techniques such as extrusion or hot pressing [6, 7, 8, 9]. A conceptual breakthrough emerged in 2017, when it was demonstrated that specific MOFs can access a liquid state prior to decomposition, opening new avenues toward thermally processable porous materials [10]. Nevertheless, melting in these systems necessarily entails the displacement or rupture of the coordination bonds sustaining the porous architecture, an energetically demanding process that results in high melting temperatures (typically above 300°C) and introduces uncertainty regarding the final structure and porous properties of the resulting material [11, 12, 13, 14, 15, 16, 17, 18].

In this context, discrete porous molecules offer a fundamentally different design strategy. Metal–organic polyhedra (MOPs) are porous molecular cages stabilized by strong intramolecular coordination bonds that preserve their intrinsic cavities, whereas their long‐range organization and bulk physical state are governed by comparatively weak intermolecular (i.e., MOP‐MOP) interactions [19, 20]. This hierarchical bonding scheme effectively decouples structural integrity from macroscopic organization. This unique feature was first exploited in 2016, when Hosono, Kitagawa, and co‐workers reported the discovery of the first glassy MOP materials [21]. Shortly after, Horike and co‐workers demonstrated that Cu(II)‐based MOPs could also display melting behavior [22]. In these pioneering studies, however, the porosity of the resulting glassy or molten‐derived phases was not evaluated. Permanent porosity in meltable cages was not confirmed until 2023, when Huang and co‐workers reported a MOP constructed from Zn(II) oxoclusters linked to *p‐tert*‐butylsulfonylcalix[4]arene and a PEG‐imidazolium‐functionalized isophthalic derivative [23]. This material exhibited a melting temperature (T_m_) of 58°C and a maximum CO_2_ uptake of ca. 0.25 mmol g^−1^ at 1 bar and 298 K. In 2024, two independent studies from Furukawa and co‐workers and our group expanded the family of porous meltable MOPs to Rh(II)‐based cages, namely MOP‐1A (T_m_ = 25°C) and BCN‐93 (T_m_ = 47°C), respectively [24, 25]. The maximum CO_2_ uptake at 1 bar for MOP‐1A and BCN‐93 was 0.04 mmol g^−1^ (303 K) and 0.2 mmol g^−1^ (298 K), respectively.

While these pioneering examples established meltable cages as a promising class of thermally processable porous materials, they revealed two key limitations: modest gas uptake capacities and limited scalability, the latter arising from synthetically demanding building blocks or the use of expensive metal nodes [26, 27, 28, 29].

Herein, we expand the chemical scope of porous meltable cages by introducing polymer‐grafted Cu(II)‐based MOPs into this emerging family. This advance is particularly significant because Cu(II)‐based MOPs, despite their synthetic accessibility and scalability, have traditionally been considered poor candidates for meltable porous materials due to the greater lability of Cu(II) coordination bonds and the presumed risk of structural collapse upon melting. To address this challenge, we adopted a different synthetic strategy. In our previous work, polymer chains were incorporated post‐synthetically into a Rh(II)‐based MOP, taking advantage of the high robustness of the Rh(II) paddlewheel motif [24, 25]. Here, however, to avoid exposing the more labile Cu(II) cage to post‐synthetic modification, the polymer functionality was pre‐installed in the ligand prior to self‐assembly and subsequently used to construct the Cu(II)‐based MOP. This design also enabled the synthesis of the isostructural Rh(II) analogue, allowing us to assess how metal‐node stability governs melting behavior, structural retention, and porosity.

This comparative study demonstrates that the Cu(II) analogue retains both structural integrity and permanent porosity upon melting, thereby ruling out melt‐induced structural degradation. In parallel, we identified diffusion limitations in previous CO_2_ uptake measurements that had masked the intrinsic uptake capacity of meltable cages. Under optimized conditions, the synthesized Cu(II)‐based MOP delivers CO_2_ uptakes of 0.68 mmol g^−1^ at 1 bar and 298 K, establishing the highest value reported to date for meltable porous materials under these conditions. Finally, by combining low melting temperature, scalability, and melt‐processability, we demonstrate the fabrication of dense macroscopic objects that retain accessible porosity.

## 2 Results and Discussion

### 2.1 Synthesis of Rh(II)‐ and Cu(II)‐Based Meltable MOPs

**FIGURE 1:**
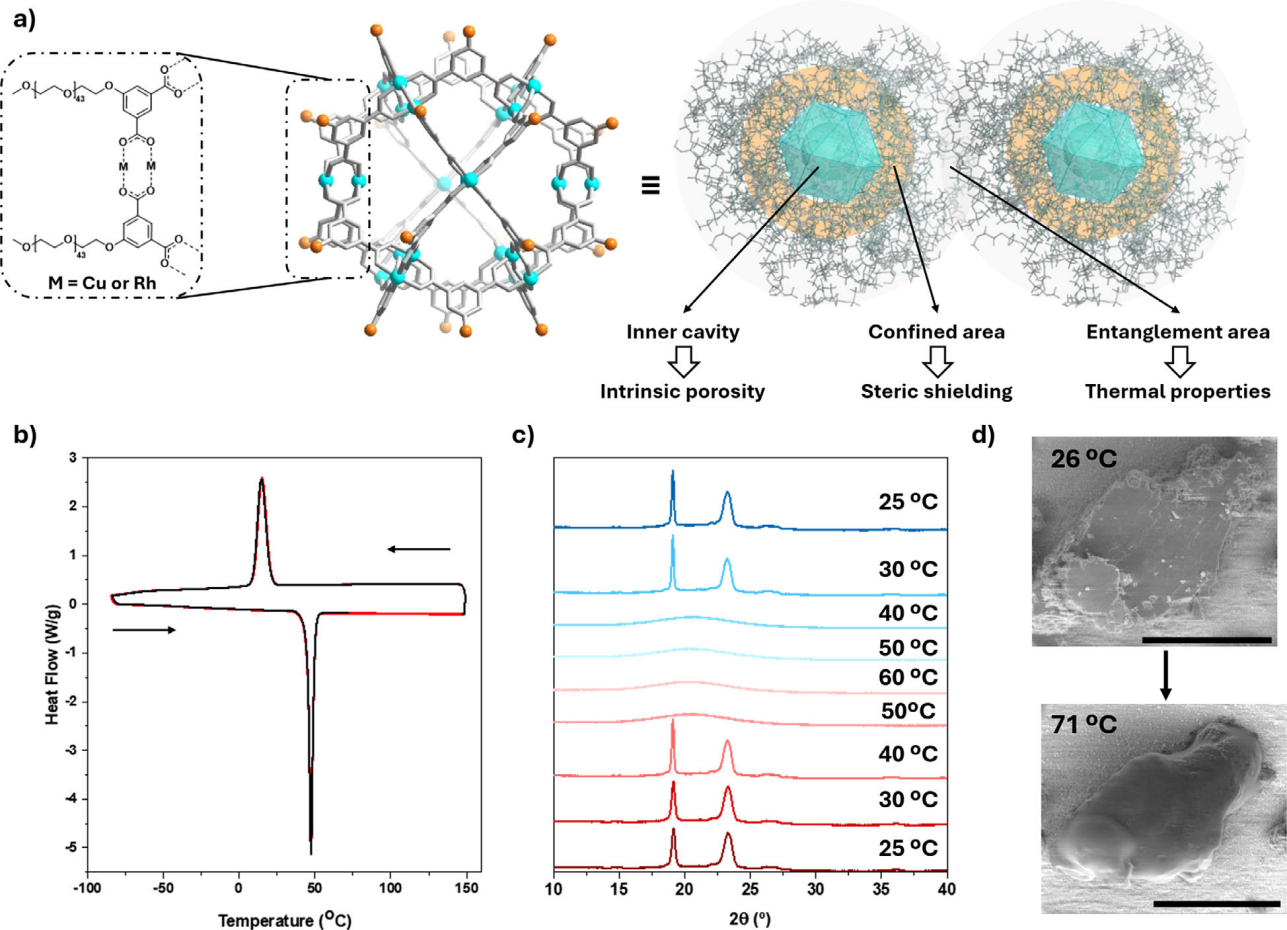
(a) Schematic of the structure of BCN‐5. (b) DSC curve of Cu‐BCN‐5 for the first (red) and second (black) cycle. (c) Variable‐temperature PXRD patterns of Cu‐BCN‐5 collected upon heating from 25 to 60°C (red) and subsequent cooling back to 25°C (blue). (d) Variable temperature FE‐SEM images of Cu‐BCN‐5 before (top) and after (bottom) in situ heating at 71°C. Scale bars = 500 µm.

The synthesis of meltable MOPs entails the installation of polymer chains on the external surface of the cage, as these polymeric coronas are responsible for the melting properties [30, 31, 32, 33]. To develop a meltable porous Cu(II)‐based MOP, we selected the archetypal cuboctahedral MOP scaffold because of its well‐established intrinsic porosity and, importantly, because it can be assembled from either Rh(II) or Cu(II) ions (Figure 1a) [34, 35]. This platform provides an ideal basis for a direct comparison between two isostructural meltable cages. Previous findings showed that surface‐bound polymer chains create a confined region at the periphery of the MOP that inhibits interpenetration between neighboring polymeric chains [25, 36]. As a result, simple linear poly(ethylene glycol) (PEG) chains can be used without bulky terminal groups, while maintaining access to the internal cavity. Regarding PEG chain length, it should be sufficiently long to escape the confined region and promote inter‐cage entanglement, but not so long as to produce extensive entanglement and high melting temperatures [33]. Accordingly, previous studies on cuboctahedral MOPs have identified PEG chains of ca. 2 kDa as an optimal compromise: shorter chains yield glassy materials with no melting transition [22], whereas longer chains would result in higher melting temperatures. Guided by these considerations, we designed a synthetic route to two isostructural meltable MOPs, Rh‐BCN‐5 and Cu‐BCN‐5 (Figure 1a).

In a typical synthesis, isophthalic acid was functionalized at the 5‐position with PEG (ca. 2000 g mol^−1^) to yield 5‐PEG_43_‐isophthalic acid (hereafter denoted H_2_L1) (Figures S1–S10). Reaction of H_2_L1 with either Rh(II) acetate or Cu(II) acetate yielded PEG‐functionalized MOPs with the general formula [M_24_L1_24_] (M = Rh or Cu). The resulting MOPs were purified by dissolution in water followed by centrifugal filtration (10 kDa cut‐off) to remove unreacted reagents. Subsequent extraction into dichloromethane and drying under vacuum afforded Rh‐BCN‐5 and Cu‐BCN‐5 as powders.

Successful cage formation was confirmed by matrix‐assisted laser desorption/ionization time‐of‐flight (MALDI‐TOF) spectrometry, which revealed broad peaks centered at 52867 g mol^−1^ for Rh‐BCN‐5 (expected: 52917 ± 4800 g mol^−1^) and 52549 g mol^−1^ for Cu‐BCN‐5 (expected: 51973 ± 4800 g mol^−1^) (Figures S11 and S15). The absence of free ligand was corroborated by Fourier transform infrared spectroscopy (FTIR): both samples displayed only coordinated carboxylate stretching bands centered at 1640 cm^−1^ (Cu‐BCN‐5) and 1570 cm^−1^ (Rh‐BCN‐5), redshifted relative to the uncoordinated carboxylic band at 1720 cm^−1^ (Figures S12 and S16). UV–vis spectra in dichloromethane revealed broad absorption bands at 614 nm for Rh‐BCN‐5 and 677 nm for Cu‐BCN‐5, consistent with the presence of the corresponding paddlewheel clusters (Figures S13 and S17) [37, 38, 39, 40]. Finally, powder x‐ray diffraction (PXRD) patterns of both as‐synthesized samples exhibited sharp reflections at 2θ = 19.4° and 23.5°, characteristic of crystalline PEG domains (Figures S14 and S18) [41]. Notably, no reflections attributable to long‐range ordering of the MOP cores were observed, indicating that crystallinity in the bulk material arises primarily from PEG chain packing rather than cage lattice organization.

### 2.2 Thermal Properties of Rh(II)‐ and Cu(II)‐Based MOPs

Once both Cu(II) and Rh(II)‐MOPs were synthesized, we proceeded to evaluate their thermal behavior. Both samples underwent concomitant melting and amorphization upon heating at 44°C, as confirmed by differential scanning calorimetry (DSC), variable‐temperature powder x‐ray diffraction (VT‐PXRD), and variable‐temperature field‐emission scanning electron microscopy (VT‐FE‐SEM) (Figure 1b–d and Figures S19–S21 and S26–S28). Thermogravimetric analysis (TGA) confirmed that this transition occurred well below their decomposition temperature (T_d_ > 200°C) and did not involve any mass loss (Figures S22 and S29). Upon cooling, both MOPs recovered crystallinity below 14°C for Cu‐BCN‐5 and 7°C for Rh‐BCN‐5. These crystallization temperatures (T_c_) are significantly lower than that of the H_2_L1 ligand itself (T_c_ = 24°C), consistent with the radial organization of PEG chains on the MOP surface, which hinders their ability to reorganize into crystalline domains. The lower T_c_ of Rh‐BCN‐5 compared to Cu‐BCN‐5 is attributed to the reduced molecular mobility of the Rh(II) paddlewheel relative to the Cu(II) one, which may further hinder PEG ordering [42]. The pronounced thermal hysteresis between the crystallization and melting temperatures in both BCN‐5 analogues indicates the formation of a supercooled liquid phase. However, under ambient conditions, the kinetic stability of this metastable phase was too short to allow its direct isolation or characterization [43].

The crystallization and melting temperatures remained constant over several DSC measurement cycles, demonstrating the thermal robustness of both materials under repeated melting‐cooling cycles (Figure 1b and Figures S19 and S26). Further evidence of structural integrity after processing was obtained from MALDI‐TOF mass spectrometry, UV–vis and FTIR spectroscopy of post‐melted samples, all of which were identical to those of the pristine materials. These results confirm that the molecular structure of both MOPs is preserved throughout successive phase transitions (Figures S23–S25 and S30–S32).

### 2.3 Mechanical Properties of Rh(II)‐ and Cu(II)‐Based MOPs

**FIGURE 2:**
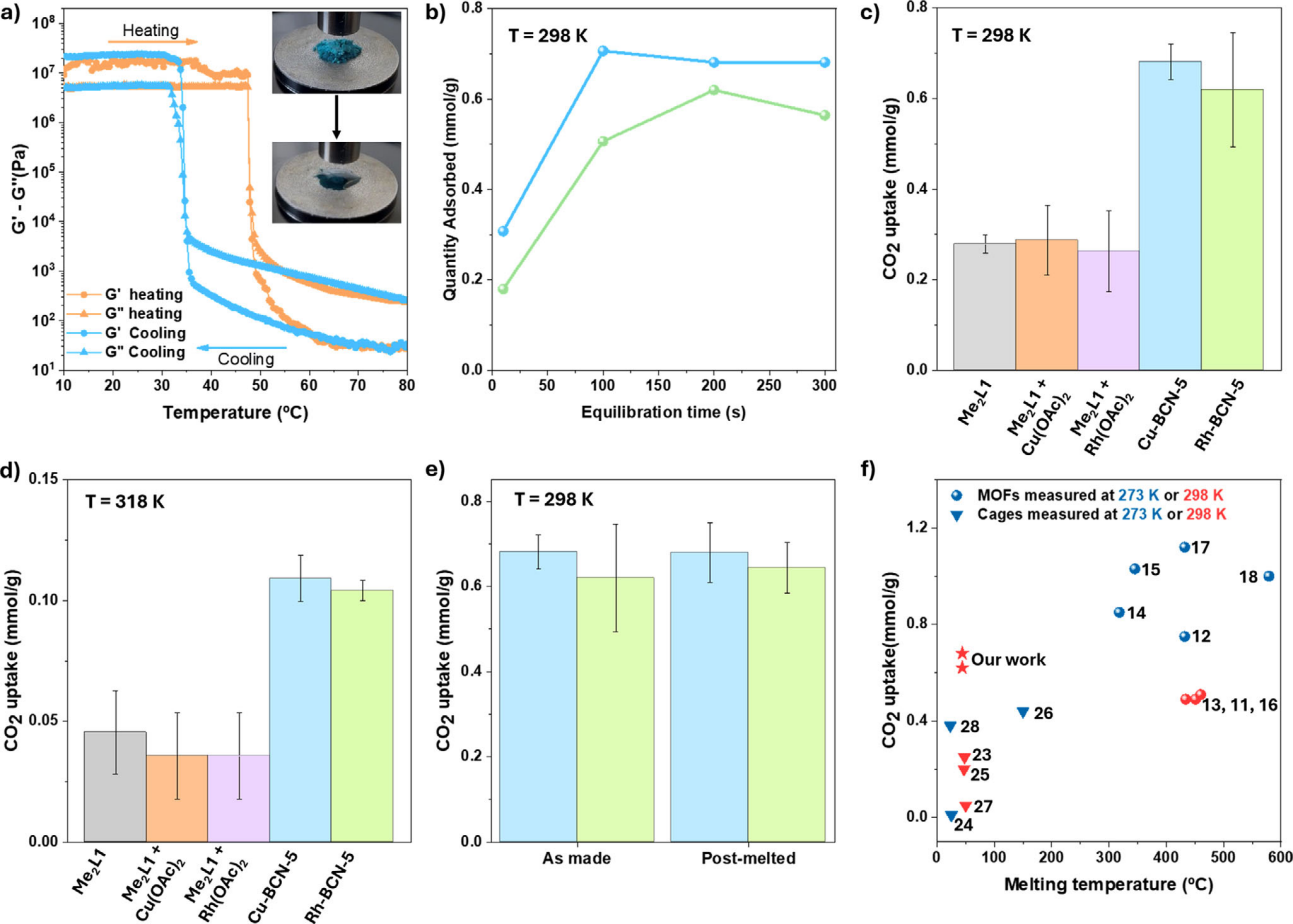
(a) Evolution of the storage modulus (G′) and the loss modulus (G″) with temperature for Cu‐BCN‐5 during a heating and cooling process. (b) Maximum CO_2_ uptake at 1 bar and 298 K for Cu‐BCN‐5 (blue) and Rh‐BCN‐5 (green) as a function of equilibration time per pressure step (10 – 300 s), obtained from adsorption isotherms. (c) CO_2_ uptake at 1 bar and 298 K of Me_2_L1 (gray), physical mixture of Me_2_L1 and copper(II) acetate (orange), physical mixture of Me_2_L1 and rhodium(II) acetate (purple), Cu‐BCN‐5 (blue) and Rh‐BCN‐5 (green). (d) CO_2_ uptake at 1 bar and 318 K of Me_2_L1 (gray), physical mixture of Me_2_L1 and copper(II) acetate (orange), physical mixture of Me_2_L1 and rhodium(II) acetate (purple), Cu‐BCN‐5 (blue) and Rh‐BCN‐5 (green). (e) CO_2_ uptake at 1 bar and 298 K for Cu‐BCN‐5 (blue) and Rh‐BCN‐5 (green), measured in the as‐made state and after a melting‐cooling cycle. (f) Comparison of reported porous meltable materials in terms of melting temperature and CO_2_ uptake at 1 bar and 273 or 298 K; each data point is labelled with its corresponding literature reference.

Variable‐temperature rheological measurements were performed to correlate the thermal phase behavior of the BCN‐5 analogues with their macroscopic mechanical response. At low temperatures (T < 40°C), both materials exhibited predominantly solid‐like or highly viscoelastic behavior, with high complex viscosities (|η*| ≈ 10^6^–10^7^ Pa s) and storage moduli (G′ ≈ 10^7^ Pa) exceeding the loss moduli (G′′ ≈ 10^6^ Pa), consistent with a semicrystalline organization of the PEG chains (Figure 2a and Figures S33–S36).

Upon heating above 47°C, both samples underwent an abrupt decrease in viscosity to the 10^1^–10^2^ Pa s regime. This drop was accompanied by a sharp decrease in G′ to the 10^2^–10^3^ Pa regime and by crossover of G′′ over G′. These results indicate a transition from a solid‐like material to a liquid phase characterized by dominant viscous dissipation. This transition closely coincides with the melting event observed by DSC and VT‐PXRD (Figure 1b,c), indicating that the loss of long‐range PEG crystallinity directly translates into a transition from a mechanically arrested state to a macroscopic flowable liquid. Upon further heating to 80°C, no signs of mechanical degradation or irreversible softening were observed, as both viscosity and moduli remained essentially constant throughout this temperature range. This behavior confirms that melting proceeds without compromising the integrity of the underlying MOP framework.

During cooling, recovery of solid‐like mechanical properties occurred over a broader temperature window below the melting temperature, reflecting the kinetic nature of PEG crystallization. Within this temperature range, the materials remained in a metastable supercooled liquid state characterized by G′′ > G′ and progressively increasing viscosity. Consistent with VT‐PXRD, crystallization at 25°C had progressed sufficiently to restore solid‐like behavior.

Therefore, rheological analysis confirms that the melting in Cu‐ and Rh‐BCN‐5 is governed by thermal reorganization of the polymer corona rather than disruption of the metal–organic core. Moreover, the marked contrast between both states highlights their processability: the liquid phase exhibits fluidity, enabling molding and extrusion, whereas the solid phase displays a high storage modulus that ensures shape retention. The facile and reversible interconversion between these two states establishes meltable MOPs as solvent‐free, reprocessable porous materials.

### 2.4 Porous Properties of Rh(II)‐ and Cu(II)‐Based MOPs

Having established that the Cu(II) derivative displays thermal behavior, stability, and mechanical properties comparable to those of its Rh(II) benchmark analogue, we next sought to analyze its sorption properties. Both analogues were found to be non‐porous to N_2_, as commonly observed for surface‐functionalized MOPs (Figures S37 and S43) [44, 45]. In contrast, the smaller kinetic diameter of CO_2_ makes it a more suitable probe for assessing porosity in surface‐functionalized MOPs [46]. Accordingly, CO_2_ adsorption isotherms were measured at 298 K up to 1 bar to evaluate their accessible porosity.

It has been reported by Yin and co‐workers that gas uptake in polymer‐grafted MOPs can be hindered by diffusion through the polymer shell, in a process resembling the solution‐diffusion mechanism described for polymeric materials and mixed‐matrix membranes [47]. As a result, equilibrium may not be reached at each targeted pressure during gas adsorption measurements, leading to underestimated capacities. To address this issue, we investigated the effect of equilibration time at each CO_2_ pressure point on the measured uptake for Rh‐ and Cu‐BCN‐5. CO_2_ adsorption isotherms were therefore collected using equilibration times of 10, 100, 200, and 300 s per pressure point. For Rh‐BCN‐5, the uptake increased from 0.18 to 0.62 mmol g^−1^ as the equilibration time was extended from 10 to 200 s (Figure 2b and Figures S38 and S39). The hysteresis observed during the desorption branch is consistent with previous reports on polymer‐grafted cages and porous liquids, where delayed desorption has been attributed to diffusion limitations [28, 48]. No further increase was observed at longer times, indicating that diffusion limitations had been overcome under these conditions. Importantly, Cu‐BCN‐5 exhibited the same trend and reached a comparable maximum uptake of 0.68 ± 0.07 mmol g^−1^, thereby confirming that replacement of Rh(II) by Cu(II) does not compromise accessible porosity (Figure 2b and Figures S44 and S45). On this basis, an equilibration time of 200 s was used for all subsequent measurements, which are reported as mean ± standard deviation from three independent samples.

The structure of BCN‐5 provides three distinct potential interaction sites for CO_2_: (i) the internal cavity, where CO_2_ can be adsorbed; (ii) the PEG shell, where CO_2_ may dissolve within transient voids; and (iii) open metal sites, where CO_2_ can bind. To assess the relative contribution of these sites, we measured CO_2_ uptake for the methyl ester derivative of H_2_L1 (Me_2_L1) and for mixtures of Me_2_L1 with the corresponding metal acetates. At 1 bar and 298 K, these control samples showed significantly lower uptakes than either BCN‐5 analogue, with values of 0.28 mmol g^−1^ for Me_2_L1, 0.29 mmol g^−1^ for Me_2_L1 with copper(II) acetate, and 0.26 mmol g^−1^ for Me_2_L1 with rhodium(II) acetate (Figure 2c and Figures S49–S54). These results indicate that the enhanced adsorption of BCN‐5 arises predominantly from the preserved cage cavity, demonstrating that the PEG corona does not occlude the pore volume.

Once the porosity of Cu‐BCN‐5 and Rh‐BCN‐5 had been confirmed in the solid state, we further evaluated both materials in their neat liquid state (Figures S42 and S48). To this end, we performed CO_2_ adsorption measurements at 318 K. Under these conditions, both analogues exhibited approximately three‐fold higher uptake than the corresponding blank samples described above, achieving a CO_2_ uptake of ca. 0.11 mmol g^−1^ (Figure 2d and Figures S41, S47 and S55–S57). This uptake places BCN‐5 among the highest‐performing Type I porous liquids reported to date (Table S1). These experiments confirm that the intrinsic cavity remains accessible even in the liquid state. Importantly, after cooling back to room temperature, the post‐melted samples exhibited CO_2_ uptakes identical to those of the as‐synthesized materials (Figure 2e and Figures S40 and S46). These results demonstrate that melting does not induce pore collapse, pore blocking, or chemical degradation, and that the intrinsic porosity of Rh‐ and Cu‐BCN‐5 is preserved throughout the melt‐solid transition.

Taken together, thermal and sorption studies position BCN‐5 analogues within a distinctive class of meltable porous materials that combine low melting temperatures with high gas uptake. This combination is particularly significant for practical implementation, as it couples solvent‐free thermal processability with persistent functional porosity (Figure 2f and Table S2).

### 2.5 Scalable Synthesis and Melt Shaping of Cu‐BCN‐5

**FIGURE 3:**
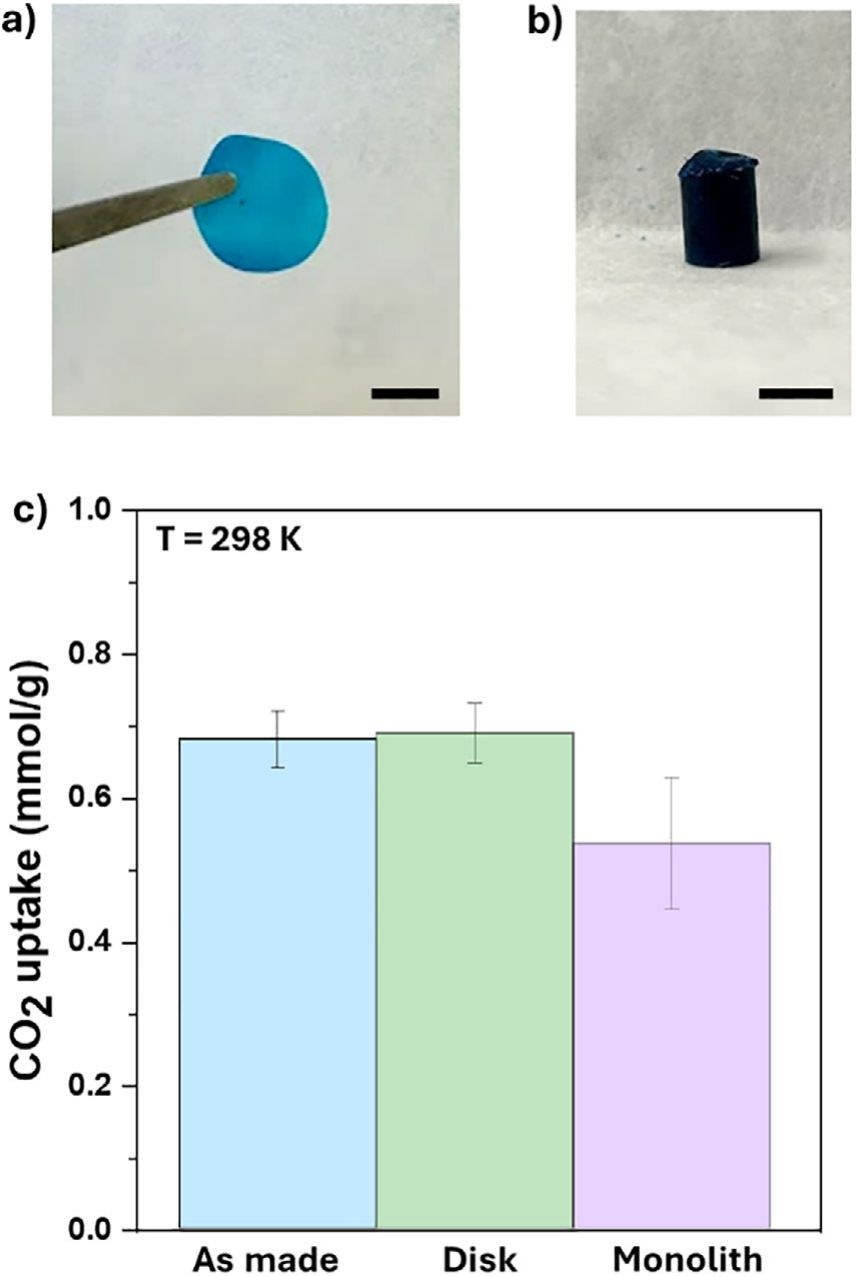
(a) Optical image of a Cu‐BCN‐5 disk. (b) Optical image of a cylindrical Cu‐BCN‐5 monolith. Scale bars = 0.5 cm (c) CO_2_ uptake at 1 bar and 298 K for Cu‐BCN‐5 in the as‐made powder (blue), disk (green), and monolith (purple) forms.

The thermal processability, mechanical properties, and preserved porosity of Cu‐BCN‐5 prompted us to explore its fabrication into macroscopic shaped objects via melt‐assisted processing methods commonly used for thermoplastic materials. To this end, we first scaled up the synthesis of Cu‐BCN‐5 to the gram scale, affording 1.8 g of material in a single batch with an isolated yield of 71 %, while maintaining the same spectroscopic, thermal, and adsorption properties as samples prepared on a smaller scale (Figures S58–S63). Flat, uniform disks were prepared by hot pressing the melt, whereas cylindrical monoliths were produced by extruding the molten material into a mold (Figure 3a,b and Figures S64 and S69).

FE‐SEM characterization of both the surface and cross‐section confirmed the monolithic nature of the processed materials, with no observable grain boundaries (Figures S65 and S70). Cross‐sectional imaging of the disks further revealed a uniform thickness of 195.0 ± 2.4 µm (Figure S66). The Young's modulus of the monolith, determined by uniaxial compression tests, was 200 ± 10 MPa, consistent with values reported for monolithic materials derived from star polymers (Figure S73) [49, 50, 51]. PXRD patterns of both shaped materials confirmed the retention of the crystalline PEG domains after processing (Figures S67 and S71).

To assess the impact of shaping on porosity, CO_2_ adsorption measurements were performed on the macroscopic materials at 298 K and 1 bar, and the results were compared with those of the as‐made powder. The disk fully retained the excellent performance of the powder, exhibiting a maximum uptake of 0.69 ± 0.04 mmol g^−1^ (Figure S68). In contrast, the monolith showed a slightly lower uptake of 0.61 ± 0.06 mmol g^−1^ (Figure S72). This ca. 20% reduction is attributed to the lower fraction of surface‐accessible MOPs in the dense, grain‐boundary‐free bulk architecture. By comparison, the thin geometry of the disk ensures a higher proportion of exposed MOPs. Such behavior mirrors trends reported for other shaped porous solids, including monolithic MOFs, where densification can modestly reduce apparent uptake while preserving intrinsic porosity [52, 53, 54, 55].

## 3 Conclusions

To date, the field of meltable porous materials has been dominated either by extended frameworks that melt only at elevated temperatures (T > 300°C) or, more recently, by proof‐of‐concept MOPs that exhibit lower melting temperatures but modest adsorption capacities. The results presented herein demonstrate that these two features, low‐temperature meltability and high adsorption capacity, do not need to be mutually exclusive. By combining readily available Cu(II) paddlewheel clusters with a simple PEG‐grafting strategy that circumvents the need for end‐capped polymers, we synthesized scalable meltable MOPs that unite thermal processability with permanent porosity. Importantly, by overcoming diffusion constraints imposed by the polymer corona during sorption measurements, we uncovered the intrinsic CO_2_ uptake capacity of these materials, reaching 0.68 ± 0.07 mmol g^−1^ at 298 K and 1 bar; to the best of our knowledge, the highest reported for meltable metal–organic materials under practically relevant conditions. Despite the significant progress achieved, the CO_2_ uptake of melt‐processable porous materials remains below that of many benchmark crystalline porous materials, which often exceed 2 mmol g^−^^1^ under comparable conditions. Bridging this gap represents an important challenge for the field and will require the simultaneous optimization of both the MOP cavity and the dynamics of the surrounding polymer corona.

Furthermore, we demonstrate that melting proceeds without compromising cage integrity, and that the liquid state can be directly exploited to fabricate self‐standing dense macroscopic objects while preserving adsorption performance. This ability to transition reversibly between processable melts and functional porous solids provides a rare combination of manufacturability and performance in metal–organic materials. Taken together, these findings establish Cu(II)‐based polymer‐grafted MOPs as a scalable platform for highly porous, low‐melting materials, opening new opportunities for solvent‐free processing, shaped adsorbents, membranes, and other applications requiring formability without loss of function.

## Author Contributions

**Alba Cortés‐martínez**: investigation, Writing – original draft, conceptualization, methodology. **Daniel Maspoch**: conceptualization, writing – review and editing, investigation. **Arnau Carné‐sánchez**: conceptualization, investigation, writing – review and editing. **José A. Suárez del Pino**: methodology, validation. **Sergio Royuela**: methodology, validation. **Francisco Sánchez‐férez**: investigation, methodology, conceptualization. **Félix Zamora**: methodology, validation.

## Funding

This work was supported by the Spanish MINECO (project RTI2018‐095622 − B‐I00 and PID2022‐138908NB‐C31), the Catalan AGAUR (project 2021 SGR 00458), the CERCA Program/Generalitat de Catalunya, the MCIN/AEI/10.13039/501100011033, and by the European Union “NextGenerationEU”/PRTR(EUR2020‐112294). ICN2 was supported by the Severo Ochoa Centres of Excellence program, Grant CEX2021‐001214‐S, funded by MCIN/AEI/10.13039.501100011033. A.C.‐S. was indebted to the Ramón y Cajal Program (RYC2020‐029749‐I Fellowship), the CNS2024‐154427 project financed by MICIU/AEI /10.13039/501100011033. Work produced with the support of a 2025 Leonardo Grant for Scientific Research and Cultural Creation, BBVA Foundation (LEO25‐1‐17844‐CBB‐QUI‐3)..

## Conflicts of Interest

The authors declare no conflicts of interest.

## Supporting information




**Supporting File**: advs77006‐sup‐0001‐SuppMat.pdf.

## Data Availability

The data that support the findings of this study are available on request from the corresponding author. The data are not publicly available due to privacy or ethical restrictions.

## References

[1] J. Wang , Y. Zhang , Y. Su , et al., “Fine Pore Engineering In A Series Of Isoreticular Metal‐Organic Frameworks For Efficient C_2_H_2_/CO_2_ Separation,” Nature Communications 13 (2022): 200, 10.1038/s41467-021-27929-7. PMC8752597 35017555

[2] Y. Yang , P. Fernández‐Seriñán , B. Ortín‐Rubio , et al., “Merging and Clipping Nets for the Synthesis of Three‐ and Two‐Merged Net Metal–Organic Frameworks,” Journal of the American Chemical Society 147 (2025): 1344–1355, 10.1021/jacs.4c15936. 39715447 PMC11726571

[3] P. Fernández‐Seriñán , K. Roztocki , V. Safarifard , et al., “Modulation of the Dynamics of a Two‐Dimensional Interweaving Metal–Organic Framework Through Induced Hydrogen Bonding,” Inorganic Chemistry 63 (2024): 5552–5558, 10.1021/acs.inorgchem.3c04522. 38484385 PMC10966731

[4] M. Eddaoudi , J. Kim , N. Rosi , et al., “Systematic Design Of Pore Size And Functionality In Isoreticular MOFs And Their Application In Methane Storage,” Science 295 (2002): 469–472, 10.1126/science.1067208. 11799235

[5] H. Deng , S. Grunder , K. E. Cordova , et al., “Large‐Pore Apertures In A Series Of Metal‐Organic Frameworks,” Science 336 (2012): 1018–1023, 10.1126/science.1220131. 22628651

[6] M. Kim , H.‐S. Lee , D.‐H. Seo , S. J. Cho , E.‐C. Jeon , and H. R. Moon , “Melt‐Quenched Carboxylate Metal–Organic Framework Glasses,” Nature Communications 15 (2024): 1174, 10.1038/s41467-024-45326-8. PMC10853212 38331892

[7] A. M. Bumstead , M. L. Ríos Gómez , M. F. Thorne , et al., “Investigating The Melting Behaviour Of Polymorphic Zeolitic Imidazolate Frameworks,” CrystEngComm 22 (2020): 3627–3637, 10.1039/D0CE00408A.

[8] C. Healy , K. M. Patil , B. H. Wilson , et al., “The Thermal Stability Of Metal‐Organic Frameworks,” Coordination Chemistry Reviews 419 (2020): 213388, 10.1016/j.ccr.2020.213388.

[9] V. Nozari , C. Calahoo , J. M. Tuffnell , D. A. Keen , T. D. Bennett , and L. Wondraczek , “Ionic Liquid Facilitated Melting Of The Metal‐Organic Framework ZIF‐8,” Nature Communications 12 (2021): 5703, 10.1038/s41467-021-25970-0. PMC8481281 34588462

[10] R. Gaillac , P. Pullumbi , K. A. Beyer , et al., “Liquid Metal–Organic Frameworks,” Nature Materials 16 (2017): 1149–1154, 10.1038/nmat4998. 29035353

[11] C. Zhou , L. Longley , A. Krajnc , et al., “Metal‐Organic Framework Glasses With Permanent Accessible Porosity,” Nature Communications 9 (2018): 5042, 10.1038/s41467-018-07532-z. PMC6262007 30487589

[12] L. Frentzel‐Beyme , M. Kloß , R. Pallach , et al., “Porous Purple Glass—A Cobalt Imidazolate Glass With Accessible Porosity From A Meltable Cobalt Imidazolate Framework,” Journal of Materials Chemistry A 7 (2019): 985–990, 10.1039/C8TA08016J.

[13] Y. Wang , H. Jin , Q. Ma , et al., “A MOF Glass Membrane for Gas Separation,” Angewandte Chemie International Edition 59 (2020): 4365–4369, 10.1002/anie.201915807. 31893511

[14] A. M. Bumstead , C. Castillo‐Blas , I. Pakamore , et al., “Formation Of A Meltable Purinate Metal–Organic Framework And Its Glass Analogue,” Chemical Communications 59 (2023): 732–735, 10.1039/D2CC05314D. 36541403

[15] A. M. Bumstead , I. Pakamore , K. D. Richards , et al., “Post‐Synthetic Modification of a Metal–Organic Framework Glass,” Chemistry of Materials 34 (2022): 2187–2196, 10.1021/acs.chemmater.1c03820. 35578693 PMC9100367

[16] H. Xia , H. Jin , Y. Zhang , et al., “A Long‐Lasting TIF‐4 MOF Glass Membrane For Selective CO_2_ Separation,” Journal of Membrane Science 655 (2022): 120611, 10.1016/j.memsci.2022.120611.

[17] S. Li , C. Ma , J. Hou , et al., “Highly Porous Metal‐Organic Framework Glass Design And Application For Gas Separation Membranes,” Nature Communications 16 (2025): 1622, 10.1038/s41467-025-56295-x. PMC11825956 39948062

[18] L. Frentzel‐Beyme , P. Kolodzeiski , J.‐B. Weiß , A. Schneemann , and S. Henke , “Quantification Of Gas‐Accessible Microporosity In Metal‐Organic Framework Glasses,” Nature Communications 13 (2022): 7750, 10.1038/s41467-022-35372-5. PMC9751146 36517486

[19] K. Omoto , N. Hosono , M. Gochomori , and S. Kitagawa , “Paraffinic Metal–Organic Polyhedrons: Solution‐Processable Porous Modules Exhibiting Three‐Dimensional Molecular Order,” Chemical Communications 54 (2018): 7290–7293, 10.1039/C8CC03705A. 29863206

[20] G. A. Craig , P. Larpent , S. Kusaka , R. Matsuda , S. Kitagawa , and S. Furukawa , “Switchable Gate‐Opening Effect In Metal–Organic Polyhedra Assemblies Through Solution Processing,” Chemical Science 9 (2018): 6463–6469, 10.1039/C8SC02263A. 30310576 PMC6115636

[21] N. Hosono , M. Gochomori , R. Matsuda , H. Sato , and S. Kitagawa , “Metal–Organic Polyhedral Core as a Versatile Scaffold for Divergent and Convergent Star Polymer Synthesis,” Journal of the American Chemical Society 138 (2016): 6525–6531, 10.1021/jacs.6b01758. 27119553

[22] S. S. Nagarkar , M. Tsujimoto , S. Kitagawa , N. Hosono , and S. Horike , “Modular Self‐Assembly and Dynamics in Coordination Star Polymer Glasses: New Media for Ion Transport,” Chemistry of Materials 30 (2018): 8555–8561, 10.1021/acs.chemmater.8b03481.

[23] C. He , Y.‐H. Zou , D.‐H. Si , et al., “A Porous Metal‐Organic Cage Liquid For Sustainable CO_2_ Conversion Reactions,” Nature Communications 14 (2023): 3317, 10.1038/s41467-023-39089-x. PMC10247695 37286561

[24] P.‐C. Han , C.‐H. Chuang , S.‐W. Lin , et al., “Phase‐Transformable Metal‐Organic Polyhedra For Membrane Processing And Switchable Gas Separation,” Nature Communications 15 (2024): 9523, 10.1038/s41467-024-53560-3. PMC11560977 39537589

[25] C. von Baeckmann , J. Martínez‐Esaín , J. A. Suárez del Pino , et al., “Porous and Meltable Metal–Organic Polyhedra for the Generation and Shaping of Porous Mixed‐Matrix Composites,” Journal of the American Chemical Society 146 (2024): 7159–7164, 10.1021/jacs.4c00407. 38467030 PMC10958503

[26] M. C. Brand , F. Greenwell , R. Clowes , et al., “Melt‐Quenched Porous Organic Cage Glasses,” Journal of Materials Chemistry A 9 (2021): 19807–19816, 10.1039/D1TA01906F.

[27] K. Jie , N. Onishi , J. A. Schott , et al., “Transforming Porous Organic Cages Into Porous Ionic Liquids via a Supramolecular Complexation Strategy,” Angewandte Chemie International Edition 59 (2020): 2268–2272, 10.1002/anie.201912068. 31778000

[28] L. Tan , K. Zheng , J.‐H. Zhou , et al., “Direct Liquefying Organic Cages Into Porous Liquid Molecules For Enhanced Near‐Infrared Photothermal Conversion And Catalysis,” Nature Communications 16 (2025): 8033, 10.1038/s41467-025-63126-6. PMC12394646 40877236

[29] X. Xiang , Z. Wang , M. Yamashita , et al., “Viscosity and Pore Accessibility Control of Type I Porous Liquids by Modifying Surface Functionality of Metal‐Organic Polyhedra,” ChemRxiv (2026), 10.26434/chemrxiv.15001444.v1. 42619071

[30] L. Huang and K. Nishinari , “Interaction Between Poly(Ethylene Glycol) And Water As Studied By Differential Scanning Calorimetry,” Journal of Polymer Science Part B: Polymer Physics 39 (2001): 496–506, 10.1002/1099-0488(20010301)39:5<496.

[31] A. Z. Samuel and S. Umapathy , “Energy Funneling And Macromolecular Conformational Dynamics: A 2D Raman Correlation Study Of PEG Melting,” Polymer Journal 46 (2014): 330–336, 10.1038/pj.2014.10.

[32] R. Paberit , E. Rilby , J. Göhl , et al., “Cycling Stability of Poly(ethylene glycol) of Six Molecular Weights: Influence of Thermal Conditions for Energy Applications,” ACS Applied Energy Materials 3 (2020): 10578–10589, 10.1021/acsaem.0c01621.

[33] F. Sánchez‐Férez , A. Carné‐Sánchez , and D. Maspoch , “Transferring Porosity Across Physical States Using Metal–Organic Cages: Porous Liquids, Glasses, Rubbers, and More,” Angewandte Chemie International Edition 65 (2026): 21455, 10.1002/anie.202521455. PMC12759227 41243760

[34] S. Furukawa , N. Horike , M. Kondo , et al., “Rhodium‐Organic Cuboctahedra as Porous Solids With Strong Binding Sites,” Inorganic Chemistry 55 (2016): 10843–10846, 10.1021/acs.inorgchem.6b02091. 27748586

[35] M. Eddaoudi , J. Kim , J. B. Wachter , H. K. Chae , M. O'Keeffe , and O. M. Yaghi , “Porous Metal−Organic Polyhedra: 25 Å Cuboctahedron Constructed From 12 Cu_2_(CO_2_)_4_ Paddle‐Wheel Building Blocks,” Journal of the American Chemical Society 123 (2001): 4368–4369, 10.1021/ja0104352. 11457217

[36] M. Zhang , Y. Lai , M. Li , et al., “The Microscopic Structure–Property Relationship of Metal–Organic Polyhedron Nanocomposites,” Angewandte Chemie International Edition 58 (2019): 17412–17417, 10.1002/anie.201909306. 31545541

[37] E. Warzecha , T. C. Berto , C. C. Wilkinson , and J. F. Berry , “Rhodium Rainbow: A Colorful Laboratory Experiment Highlighting Ligand Field Effects of Dirhodium Tetraacetate,” Journal of Chemical Education 96 (2019): 571–576, 10.1021/acs.jchemed.6b00648.

[38] A. Cortés‐Martínez , C. von Baeckmann , L. Hernández‐López , A. Carné‐Sánchez , and D. Maspoch , “Giant Oligomeric Porous Cage‐Based Molecules,” Chemical Science 15 (2024): 7992–7998, 10.1039/D4SC01974A. 38817590 PMC11134396

[39] S. Ruiz‐Relaño , D. Nam , J. Albalad , et al., “Synthesis of Metal–Organic Cages via Orthogonal Bond Cleavage in 3D Metal–Organic Frameworks,” Journal of the American Chemical Society 146 (2024): 26603–26608, 10.1021/jacs.4c09431. 39311525 PMC11450890

[40] E. B. Boyar and S. D. Robinson , “Rhodium(II) Carboxylates″,” Coordination Chemistry Reviews 50 (1983): 109–208, 10.1016/0010-8545(83)85028-0.

[41] R. Li , Y. Wu , Z. Bai , J. Guo , and X. Chen , “Effect Of Molecular Weight Of Polyethylene Glycol On Crystallization Behaviors, Thermal Properties And Tensile Performance Of Polylactic Acid Stereocomplexes,” RSC Advances 10 (2020): 42120–42127, 10.1039/D0RA08699A. 35516761 PMC9057859

[42] A. Khobotov‐Bakishev , C. von Baeckmann , B. Ortín‐Rubio , et al., “Multicomponent, Functionalized HKUST‐1 Analogues Assembled via Reticulation of Prefabricated Metal–Organic Polyhedral Cavities,” Journal of the American Chemical Society 144 (2022): 15745–15753, 10.1021/jacs.2c06131. 35973046 PMC9437915

[43] S. Napolitano , E. Glynos , and N. B. Tito , “Glass Transition Of Polymers In Bulk, Confined Geometries, And Near Interfaces,” Reports on Progress in Physics 80 (2017): 036602, 10.1088/1361-6633/aa5284. 28134134

[44] E. V. Perez , K. J. Balkus , J. P. Ferraris , and I. H. Musselman , “Metal‐Organic Polyhedra 18 Mixed‐Matrix Membranes for Gas Separation,” Journal of Membrane Science 463 (2014): 82–93, 10.1016/j.memsci.2014.03.045.

[45] T.‐H. Chen , L. Wang , J. V. Trueblood , V. H. Grassian , and S. M. Cohen , “Poly(isophthalic acid)(ethylene oxide) as a Macromolecular Modulator for Metal–Organic Polyhedra,” Journal of the American Chemical Society 138, no. 30 (2016): 9646–9654, 10.1021/jacs.6b04971. 27400759

[46] A. J. Gosselin , C. A. Rowland , and E. D. Bloch , “Permanently Microporous Metal–Organic Polyhedra,” Chemical Reviews 120, no. 16 (2020): 8987–9014, 10.1021/acs.chemrev.9b00803. 32519860

[47] Y. Lai , J. Yang , L. Cai , et al., “Precise Modulation of Surface Layer Dynamics for Tunable Flowability and Gas Absorption Properties of Molecular Porous Liquids,” Advanced Functional Materials 33 (2023): 2210122, 10.1002/adfm.202210122.

[48] A. Ganesan and S. Dai , “Porous Liquids: Versatile Fusion of Porosity and Fluidity,” Chemistry of Materials 36, no. 20 (2024): 10003–10007, 10.1021/acs.chemmater.4c02399.

[49] S. Basu , A. Cano‐Odena , and I. F. J. Vankelecom , “Asymmetric Matrimid®/[Cu_3_(BTC)_2_] Mixed‐Matrix Membranes For Gas Separations,” Journal of Membrane Science 362 (2010): 478–487, 10.1016/j.memsci.2010.07.005.

[50] J. C. Tan and A. K. Cheetham , “Mechanical Properties Of Hybrid Inorganic–Organic Framework Materials: Establishing Fundamental Structure–Property Relationships,” Chemical Society Reviews 40 (2011): 1059, 10.1039/c0cs00163e. 21221446

[51] Y. L. Lee , D. W. Lester , J. R. Jones , and T. K. Georgiou , “Effect of Polymer Molecular Mass and Structure on the Mechanical Properties of Polymer–Glass Hybrids,” ACS Omega 7 (2022): 786–792, 10.1021/acsomega.1c05424. 35036745 PMC8757365

[52] J. Hou , A. F. Sapnik , and T. D. Bennett , “Metal–Organic Framework Gels And Monoliths,” Chemical Science 11 (2020): 310–323, 10.1039/C9SC04961D. 32153752 PMC7021205

[53] X. Yu , B. Li , L. Wu , D. Shi , and S. Han , “Review and Perspectives of Monolithic Metal–Organic Frameworks: Toward Industrial Applications,” Energy & Fuels 37 (2023): 9938–9955, 10.1021/acs.energyfuels.3c00858.

[54] D. Muringaniza , L. Zulu , L. G. Marazani , P. Tshuma , and G. Mehlana , “Recent Progress In Synthesis And Applications Of Monolithic Metal–Organic Frameworks,” Materials Advances 7 (2026): 40–82, 10.1039/D5MA01105A.

[55] D. G. Madden , R. Babu , C. Çamur , et al., “Monolithic Metal–Organic Frameworks For Carbon Dioxide Separation,” Faraday Discussions 231 (2021): 51–65, 10.1039/d1fd00017a. 34235530 PMC8517963

