# Supplementary material for: Polymer‐Grafted Cu(II) Metal–Organic Polyhedra as Scalable, Melt‐Processable Porous Materials
Source: Adv Sci (Weinh). 2026 Sep 7:e77006. Online ahead of print. doi: 10.1002/advs.77006 (PMC13548290; doi:10.1002/advs.77006)
Supplement: Supplementary file 1 [file ADVS-9999-e77006-s001.pdf]

# Supporting Information

## Polymer-grafted Cu(II) Metal–Organic Polyhedra as Scalable, Melt-Processable Porous Materials

Alba Cortés-Martínez, Francisco Sánchez-Férez, José A. Suárez del Pino, Sergio Royuela, Félix Zamora, Daniel Maspoch*, and Arnau Carné-Sánchez*

# Table of contents


S1. Materials ........ 3

S2. Synthetic procedures ........ 5

S2.1. Synthesis of *5-PEG-isophthalic acid* ($H_2L1$): ........ 5

S2.2. Synthesis of Rh-BCN-5 ........ 5

S2.3. Synthesis of Cu-BCN-5 ........ 6

S2.4. Scale-up synthesis of Cu-BCN-5 ........ 6

S2.5. Formation of Cu-BCN-5 disk ........ 6

S2.6. Formation of Cu-BCN-5 monolith ........ 7

S2.7. Sample activation ........ 7

S2.8. $CO_2$ adsorption measurements of liquid samples ........ 7

S2.9. Preparation of blank samples ........ 7

S3. Characterization ........ 8

S3.1. $Me_2L1$ ........ 8

S3.2. $H_2L1$ ........ 13

S3.3. Characterization Rh and Cu-BCN-5 ........ 18

S3.3.1. Rh-BCN-5 ........ 18

S3.3.2. Cu-BCN-5 ........ 22

S3.4. Thermal properties and stability ........ 26

S3.4.1. Rh-BCN-5 ........ 26

S3.4.2. Cu-BCN-5 ........ 33

S3.5. Mechanical properties ........ 40

S3.5.1. Rh-BCN-5 ........ 40

S3.5.2. Cu-BCN-5 ........ 42

S3.6. Porous properties S3.6.1. Rh-BCN-5 ........ 44

S3.6.2. Cu-BCN-5 ........ 50

S3.6.3. Blank samples ........ 58

S3.7. Scale-up synthesis of Cu-BCN-5 ........ 67

S3.8. Shaping ........ 73

S3.8.1. Cu-BCN-5 disk ........ 73

S3.8.2. Cu-BCN-5 monolith ........ 78

S4. References ........ 83

## S1. Materials

Rhodium(II) acetate was purchased from ABCR. $Na_2CO_3$, LiOH, $K_2CO_3$, $MgSO_4$, copper(II) acetate were purchased from Sigma-Aldrich. Diethyl 5-hydroxisphtalate were purchased from BLD. α-methoxy-ω-bromo-PEG was purchased from RappPolymere. All deuterated solvents were purchased from Eurisotop. Solvents at HPLC grade were purchased from Fischer Chemicals.

**Ultraviolet-Visible (UV–Vis)** spectra were measured using a Thermo Scientific™ NanoDrop 2000 at room temperature (*ca.* 25 °C).

**Proton Nuclear Magnetic Resonance ($^1$H-NMR)** spectra were acquired using Bruker Ascend 300 MHz at "Servei de Resonància Magnètica Nuclear" from Autonomous University of Barcelona (UAB). Chemical shifts (δ) for $^1$H-NMR spectra are reported in parts per million (ppm) and relative to the solvent residual peak.

**Diffusion Ordered NMR Spectroscopy (DOSY)** spectra were acquired using Bruker Ascend 300 MHz at "Servei de Resonància Magnètica Nuclear" from Autonomous University of Barcelona (UAB).

**Matrix Assisted Laser Desorption/Ionization mass spectrometry (MALDI-TOF)** measurements were performed using a 4800 Plus MALDI TOF/TOF (ABSCIEX – 2010). All samples were analysed in positive ionization mode using trans-2-[3-(4-*tert*-butylphenyl)-2-methyl-2-propenylidene]malononitrile (DCTB) as ionization matrix.

**Fourier-Transform Infrared Spectroscopy (FTIR)** spectra were acquired on a Bruker IR Alpha II spectrometer equipped with a diamond attenuated total reflection (ATR) accessory. All spectra were collected in neat samples in ambient atmosphere.

**Thermogravimetric analysis (TGA)** was performed on a Netzsch STA 449F1 instrument from 23 to 800 °C using a heating rate of 10 °C/min.

**Volumetric $CO_2$ isotherms** were collected at 298 K and 318 K using an ASAP 2020 (Micromeritics). Temperature for $CO_2$ isotherms measurement was controlled by a chiller. The standard deviation was calculated from 3 independent samples.

**Powder X-ray diffraction (PXRD)** diagrams were collected on a Panalytical X'pert diffractometer with monochromatic Cu-$K_\alpha$ radiation ($\lambda_{Cu}$ = 1.5406 Å).

**Variable temperature X-ray diffraction** (VT-XRD) measurements were performed in a TTK600 Low Temperature Chamber from Anton Paar which was mounted inside an X'pert Pro MPD Malvern Panalytical Diffractometer. This temperature chamber allows to perform studies from -20 ºC to 600 ºC using different atmospheres (vacuum, inert gases or air). In this experiment, the samples were mounted in a Zero-Background Si Holder for reflection geometry. The sample was heated up until 110 ºC under air. Different temperature steps were performed:

1st Process → From 25 ºC to 30 ºC,

increment of temperature =10 ºC/min, and a D-well= 5min to stabilize temperature in each step.

2nd Process → From 30 ºC to 40 ºC,

increment of temperature =5 ºC/min, and a D-well= 5min to stabilize temperature in each step.

3rd Process → From 40 ºC to 50 ºC,

increment of temperature =10 ºC/min, and a D-well= 5min to stabilize temperature in each step.

4th Process → from 50 ºC to 60 ºC,

increment of temperature =10 ºC/min, and a D-well= 5min to stabilize temperature in each step.

5th Process → From 60 ºC to 50 ºC,

increment of temperature =10 ºC/min, and a D-well= 5min to stabilize temperature in each step.

6th Process → From 50 ºC to 40 ºC,

increment of temperature =5 ºC/min, and a D-well= 5min to stabilize temperature in each step.

7th Process → From 40 ºC to 30 ºC,

increment of temperature =10 ºC/min, and a D-well= 5min to stabilize temperature in each step.

8th Process → from 30 ºC to 25 ºC,

increment of temperature =10 ºC/min, and a D-well= 5min to stabilize temperature in each step.

The scans were measured from 10º - 40º, with a step size of 0.01º and a counting time of 50 s.

**Differential Scanning calorimetry (DSC)** measurements were carried out using a TA Instrument DSC25 under Nitrogen atmosphere (50 mL / min) and a heating rate of 10 ºC·min$^{-1}$.

**Scanning Electron Microscopy (SEM)** images were obtained with a FEI Magellan 400L XHR microscope by supporting the sample on a conductive aluminum tape. Samples were coated with Pt (5.04 nm) using an Emitech K550X/K250 C cathodic metallizer.

**Variable temperature SEM (VT-SEM)** was performed in a FEI Quanta 650FEG ESEM microscope.

**Rheology measurements** were performed using an MCR 102e (Anton Paar, equipped with a peltier temperature control and a parallel plate geometry of 25 mm diameter). Samples were placed as powders and shaped prior to being measured. Then, disk-shaped samples were subjected to variable-temperature measurements under constant strain of 1 % from 10 ºC to 80 ºC.

**Uniaxial compression tests** were carried out on an Instron 3400 Series Universal Testing Machine with a 50 N load cell. Samples were compressed to a maximum strain of 80 % at a rate of 1.0 mm min$^{-1}$, with a preload of 0.1 N.

## S2. Synthetic procedures

### S2.1. Synthesis of *5-PEG-isophthalic acid* ($H_2L1$):

*Synthesis of dimethyl 5-PEG-isophthalate (**$Me_2L1$**)*

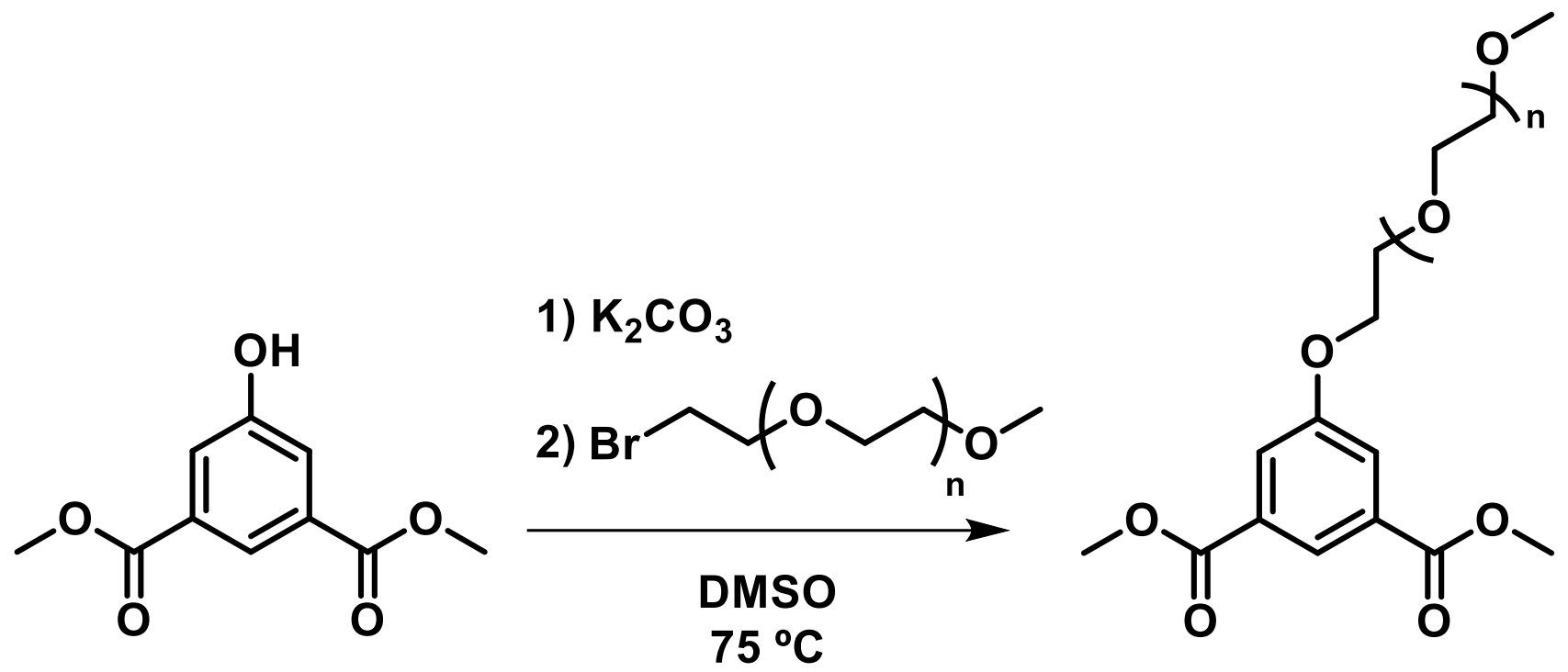


Dimethyl 5-hydroxyisophthalate (100 mg, 0.48 mmol), $K_2CO_3$ (332 mg, 2.4 mmol) and *N,N*-dimethylformamide (10 mL) were mixed and stirred at room temperature for 30 mins. Then, α-methoxy-ω-bromo-PEG (972 mg, 0.49 mmol) was added to the mixture. The resulting reaction mixture was stirred at 75 ºC overnight. After the reaction time, the crude was diluted with dichloromethane (15 mL), and the mixture was extracted with $H_2O$:MeOH (1:1, 30 mL) three times. Finally, the organic phase was dried over $MgSO_4$ and evaporated under vacuum to afford $Me_2L_1$ as a pale yellow solid (yield = 76 %).

*Synthesis of 5-PEG-isophthalic acid (**$H_2L2$**)*

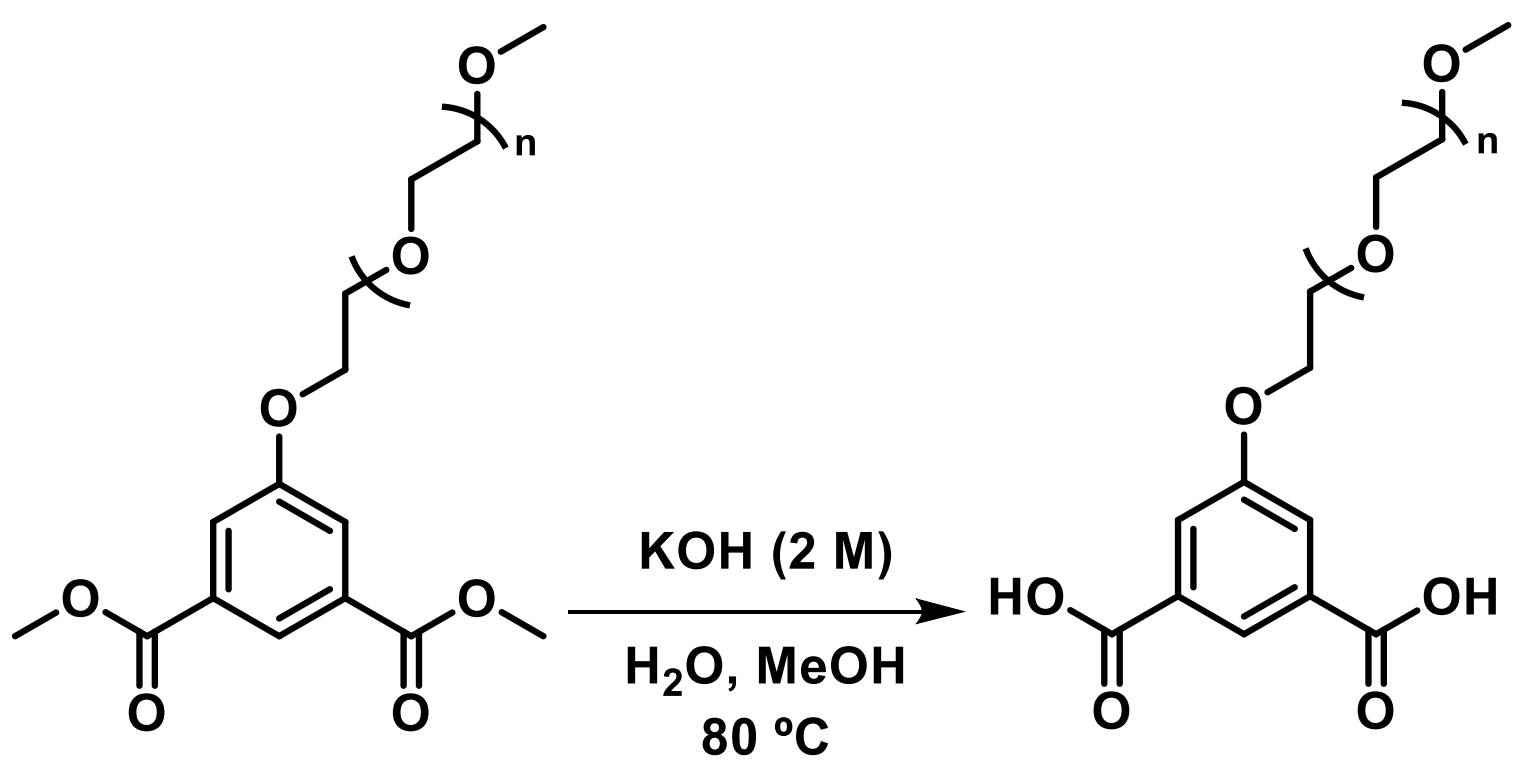


$Me_2L1$ (1.0 g, 0.47 mmol) was dissolved in methanol (25 mL) and saturated LiOH (5 mL). Then, the mixture was stirred under reflux at 85 °C overnight. After the reaction, the crude was concentrated under vacuum, followed by the addition of HCl 3 M (10 mL). Then, the solution was extracted with dichloromethane (15 mL) twice, with the addition of MeOH (15 mL) to facilitate the separation between the two phases. Subsequently, the organic phase was extracted twice with $H_2O$:MeOH (1:1, 20 mL). Finally, the organic phase was dried over $MgSO_4$ and evaporated under vacuum to afford $H_2L_1$ as a pale yellow solid (yield = 90 %).

### S2.2. Synthesis of Rh-BCN-5

Rhodium acetate (20.0 mg, 0.04 mmol), $Na_2CO_3$ (23.5 mg, 0.22 mmol), $H_2L1$ (462 mg, 0.22 mmol) and 2 mL of DMA were added into a 6 mL scintillation vial. The mixture was heated at 100 °C for

24 h. A deep green solution was obtained and separated from the residual solids by centrifugation. Then, diethyl ether (40 mL) was added to the supernatant to precipitate the crude product, which was separated by centrifugation. Subsequently, the green solid was dissolved in $H_2O$ (15 mL) and filtered by centrifugal filters with a molecular weight cut-off of 10 kDa, where our product was retained whereas unreacted reagents went through the filter. This filtering process was repeated several times with fresh water until the filtrate was colorless. Then, the aqueous solution was extracted three times with dichloromethane (5 mL), with the addition of MeOH (5 mL) to facilitate the separation between the two phases. Afterwards, the organic phase was dried under vacuum to obtain a green solid that was further washed with a mixture of diethyl ether:acetone (3:1, 40 mL) to remove any traces of water. Finally, the green solid was dried under vacuum to obtain Rh-BCN-5 (yield: 65%).

**S2.3. Synthesis of Cu-BCN-5**

Copper acetate (50.0 mg, 0.28 mmol), $H_2L1$ (520 mg, 0.25 mmol) and 2 mL of tetrahydrofuran were added into a 13 mL vial. The mixture was stirred at room temperature overnight. A deep blue solution was obtained. Then, diethyl ether (40 mL) was added to the solution to precipitate the crude product, which was separated by centrifugation. Subsequently, the blue solid was dissolved in $H_2O$ (15 mL) and filtered by centrifugal filters with a molecular weight cut-off of 10 kDa, where our product was retained whereas unreacted reagents went through the filter. This filtering process was repeated several times with fresh water until the filtrate was colorless. Then, the aqueous solution was extracted three times with dichloromethane (5 mL), with the addition of MeOH (5 mL) to facilitate the separation between the two phases. Afterwards, the organic phase was dried under vacuum to obtain a blue solid that was further washed with a mixture of diethyl ether:acetone (3:1, 40 mL) to remove any traces of water. Finally, the blue solid was dried under vacuum to obtain Cu-BCN-5 (yield: 52%).

**S2.4. Scale-up synthesis of Cu-BCN-5**

Copper acetate (240.4 mg, 1.35 mmol), $H_2L1$ (2.5 g, 1.20 mmol) and 10 mL of tetrahydrofuran were added into a 23 mL vial. The mixture was stirred at room temperature overnight. A deep blue solution was obtained. Then, diethyl ether (40 mL) was added to the solution to precipitate the crude product, which was separated by centrifugation. Subsequently, the blue solid was dissolved in $H_2O$ (15 mL) and filtered by centrifugal filters with a molecular weight cut-off of 10 kDa, where our product was retained whereas unreacted reagents went through the filter. This filtering process was repeated several times with fresh water until the filtrate was colorless. Then, the aqueous solution was extracted three times with dichloromethane (5 mL), with the addition of MeOH (5 mL) to facilitate the separation between the two phases. Afterwards, the organic phase was dried under vacuum to obtain a blue solid that was further washed with a mixture of diethyl ether:acetone (3:1, 40 mL) to remove any traces of water. Finally, the blue solid was dried under vacuum to obtain Cu-BCN-5 (yield: 71%).

**S2.5. Formation of Cu-BCN-5 disk**

Disks were prepared by hot-press processing using a rheometer (Figure S54). Approximately 15 mg of sample were placed in the rheometer and heated to 47.5 °C to induce melting of the Cu-BCN-5. Once in the liquid state, a pressure of 0.1 N was applied, and the sample was subsequently cooled to 10 °C while maintaining the applied pressure. This process yielded self-standing disks with a diameter of ca. 0.8 cm and a thickness of *ca.* 200 µm.

**S2.6. Formation of Cu-BCN-5 monolith**

A cylindrical monolith was formed by placing *ca.* 150 mg of Cu-BCN-5 inside a syringe and heating it at 65 ºC for 15 min. Once the sample was liquid, it was extruded to a mold. Then, the sample in the mold was kept at room temperature until it solidified. The resulting monolith was then removed (Figure S59). Using this method, monoliths with a diameter of approximately 0.5 cm and a height of approximately 0.6 cm were obtained.

**S2.7. Sample activation**

Prior to $CO_2$ adsorption measurements, samples were washed three times with 40 mL of a 3:1 (v/v) mixture of diethyl ether and acetone. The solids were then dried and kept under vacuum overnight at room temperature.

**S2.8. $CO_2$ adsorption measurements of liquid samples**

Approximately 100 mg of Cu- or Rh-BCN-5 was placed in an adsorption cell and heated to 65 °C for 15 min to induce melting. The temperature was then maintained at 45 °C throughout the measurement to preserve the liquid state.

**S2.9. Preparation of blank samples**

$Me_2L_1$ was selected as a blank to account for $CO_2$ adsorption by the polymer layer, rather than $H_2L_1$, in order to avoid interactions between free carboxylic acid groups and $CO_2$. Such interactions are not present in BCN-5 samples.

To evaluate the contribution of open metal sites within the polymeric medium, physical mixtures of $Me_2L_1$ with the corresponding metal acetates were prepared, maintaining the same polymer-to-metal molar ratio as in BCN-5. Specifically, $Me_2L_1$ (150 mg, 0.05 mmol) was mixed with copper(II) acetate (9.0 mg, 0.05 mmol of Cu(II)) or rhodium(II) acetate (11.0 mg, 0.025 mmol of $Rh_2(OAc)_4$, corresponding to 0.05 mmol of Rh(II) centers). In both cases, the mixtures were ground and subsequently activated under vacuum.

## S3. Characterization

### S3.1. $Me_2L1$

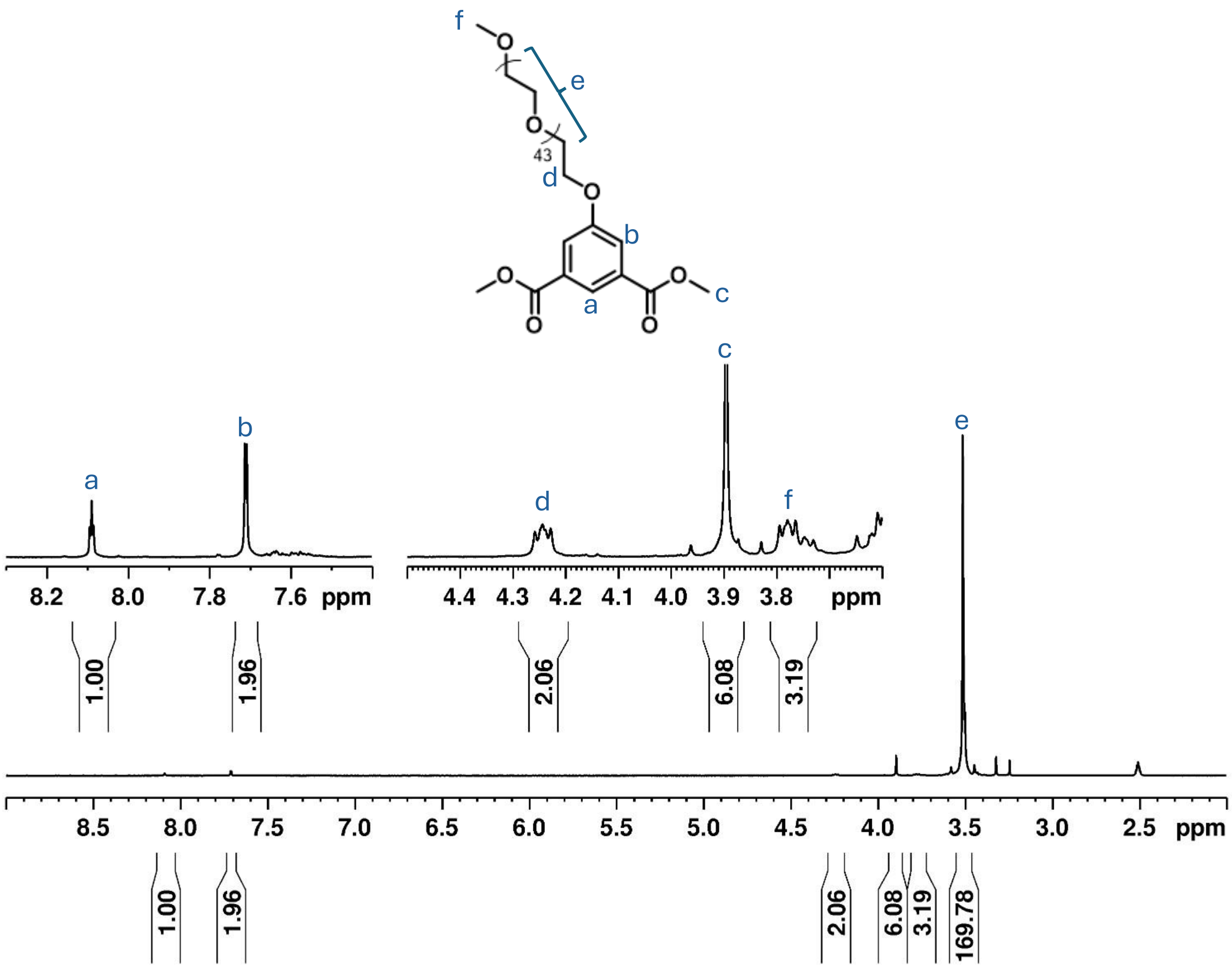


**Figure S1.** $^1$H-NMR spectrum (300 MHz, 25 ºC) of $Me_2L1$ in DMSO-$d_6$.

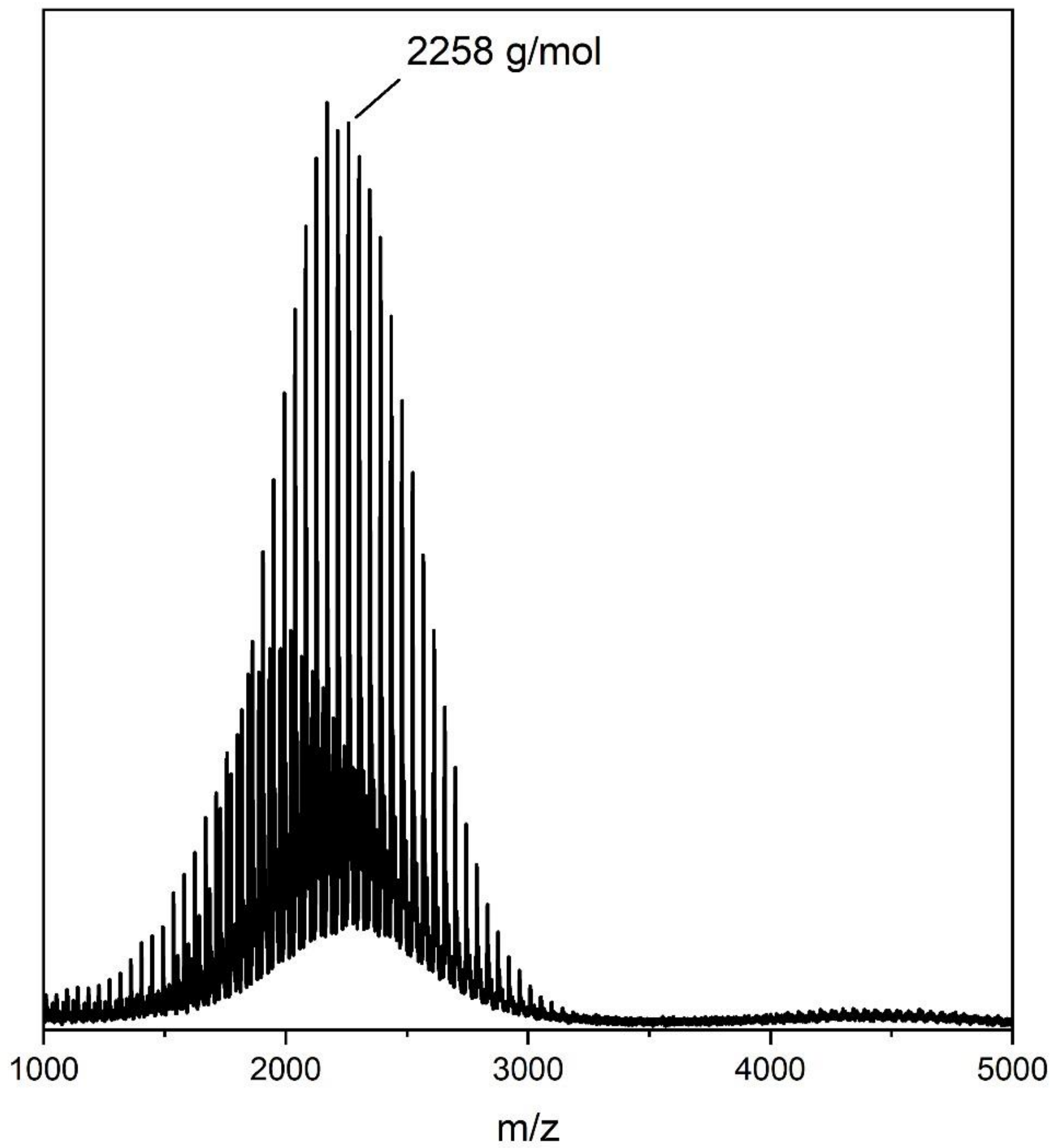


**Figure S2.** MALDI-TOF spectrum of $Me_2L1$. The highlighted mass corresponds to the molecular formula of $[C_{97}O_{49}H_{186} + H^+]^+$ (expected m/z = 2134 ± 200 g·mol$^{-1}$; found m/z = 2258 g·mol$^{-1}$).

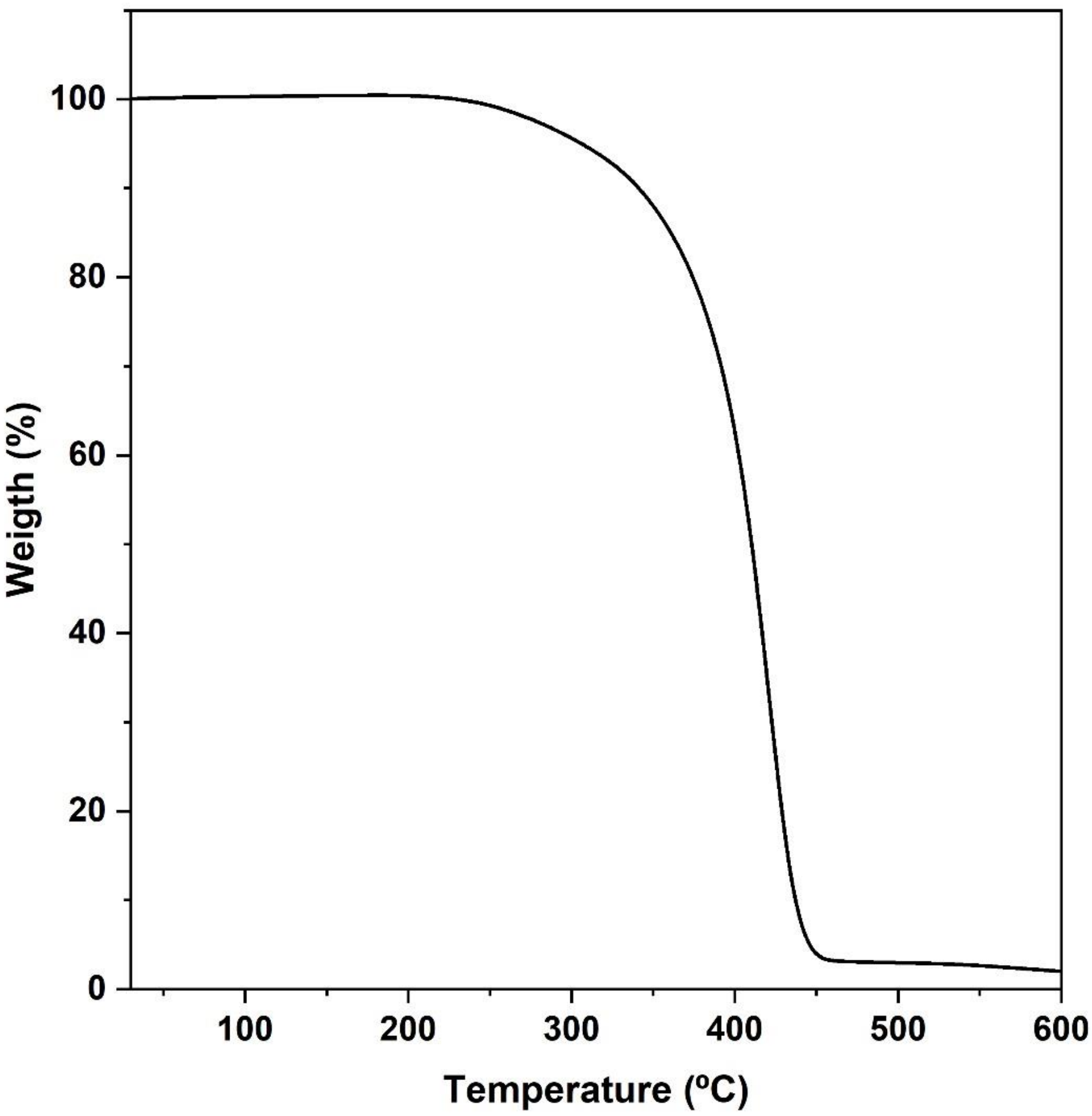


**Figure S3.** TGA curve of $Me_2L1$, showing a decomposition temperature at ca. 290 °C.

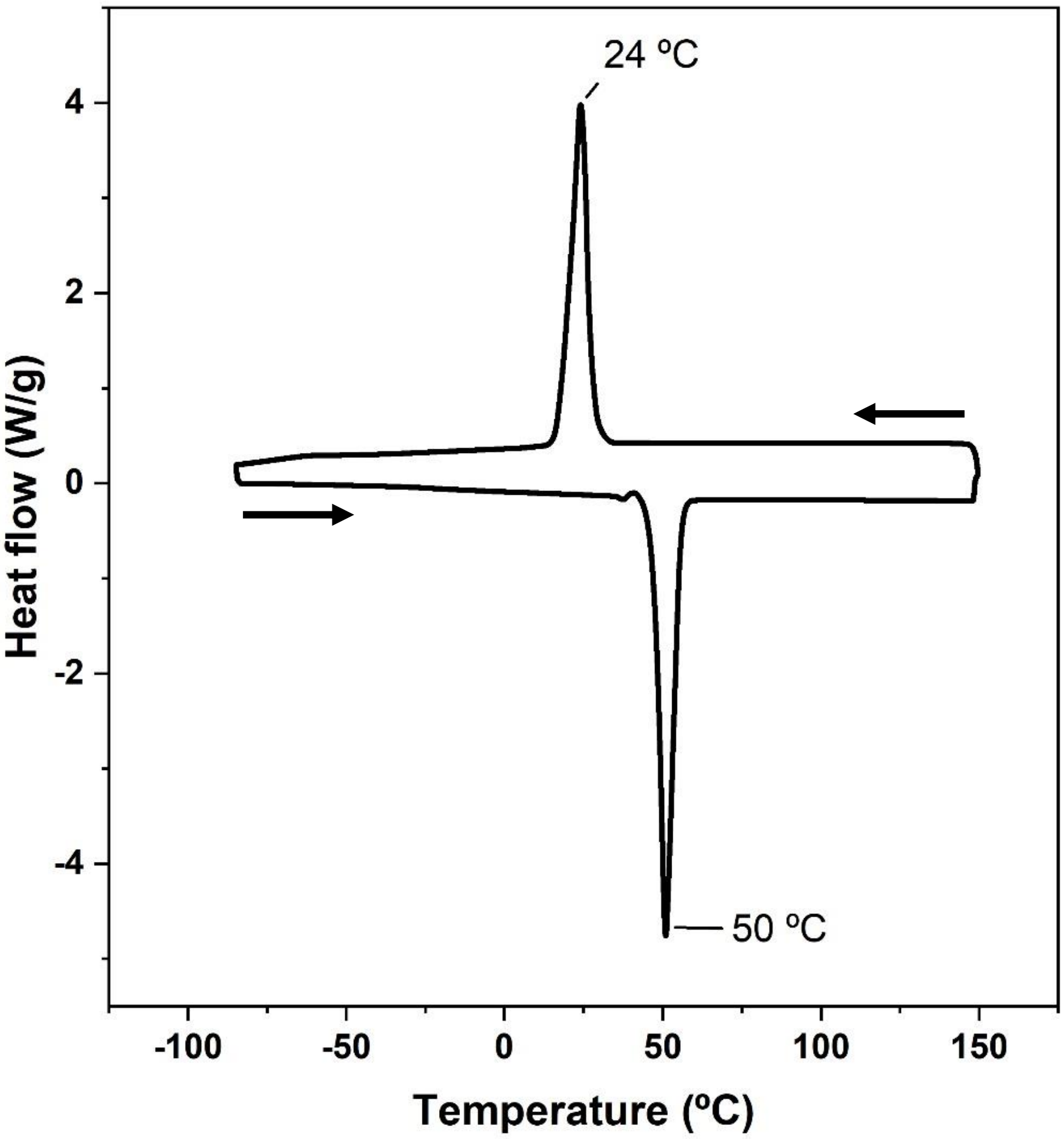


**Figure S4.** DSC curve of $Me_2L1$.

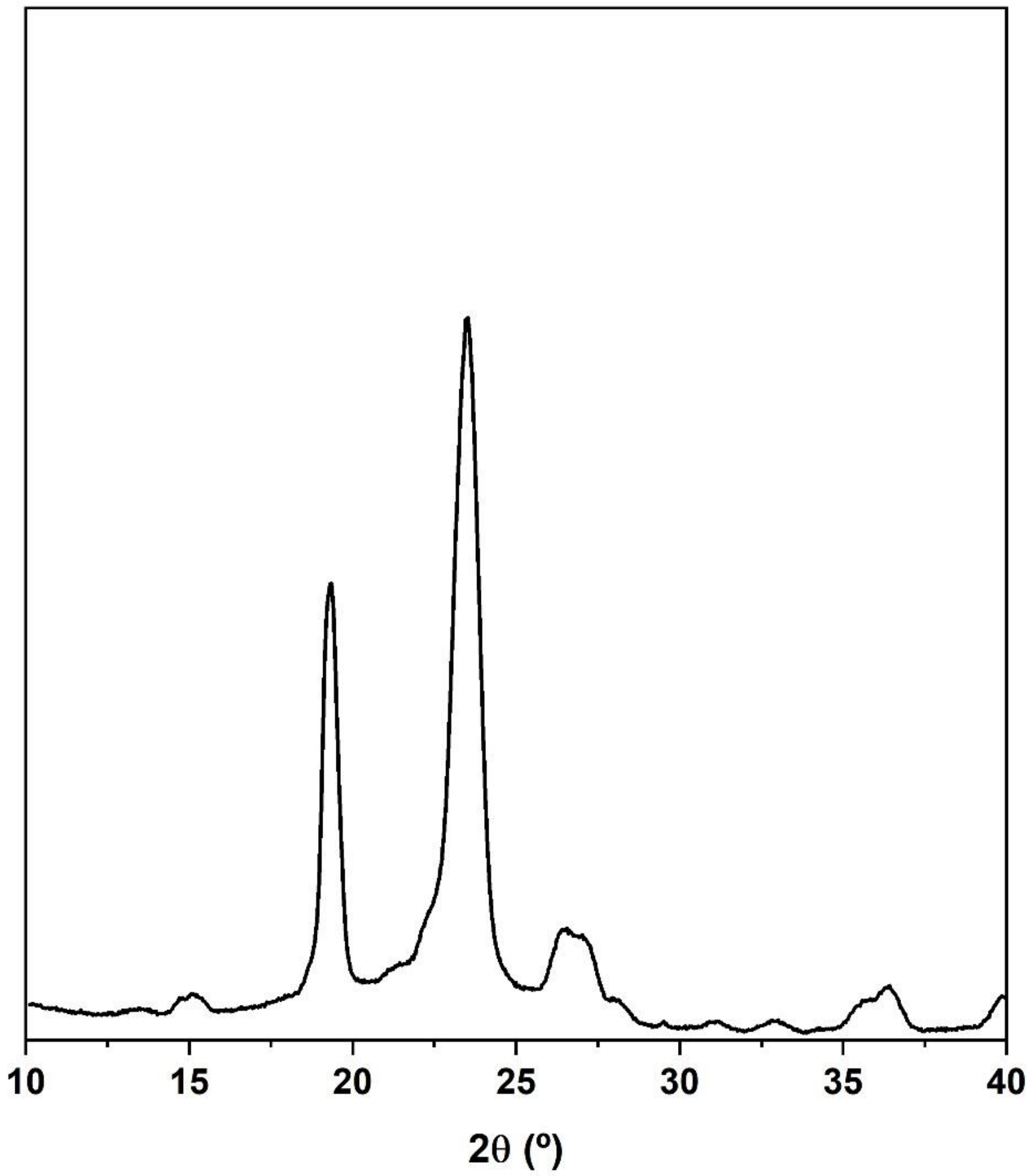


**Figure S5.** PXRD pattern of $Me_2L1$.

S3.2. $H_2L1$

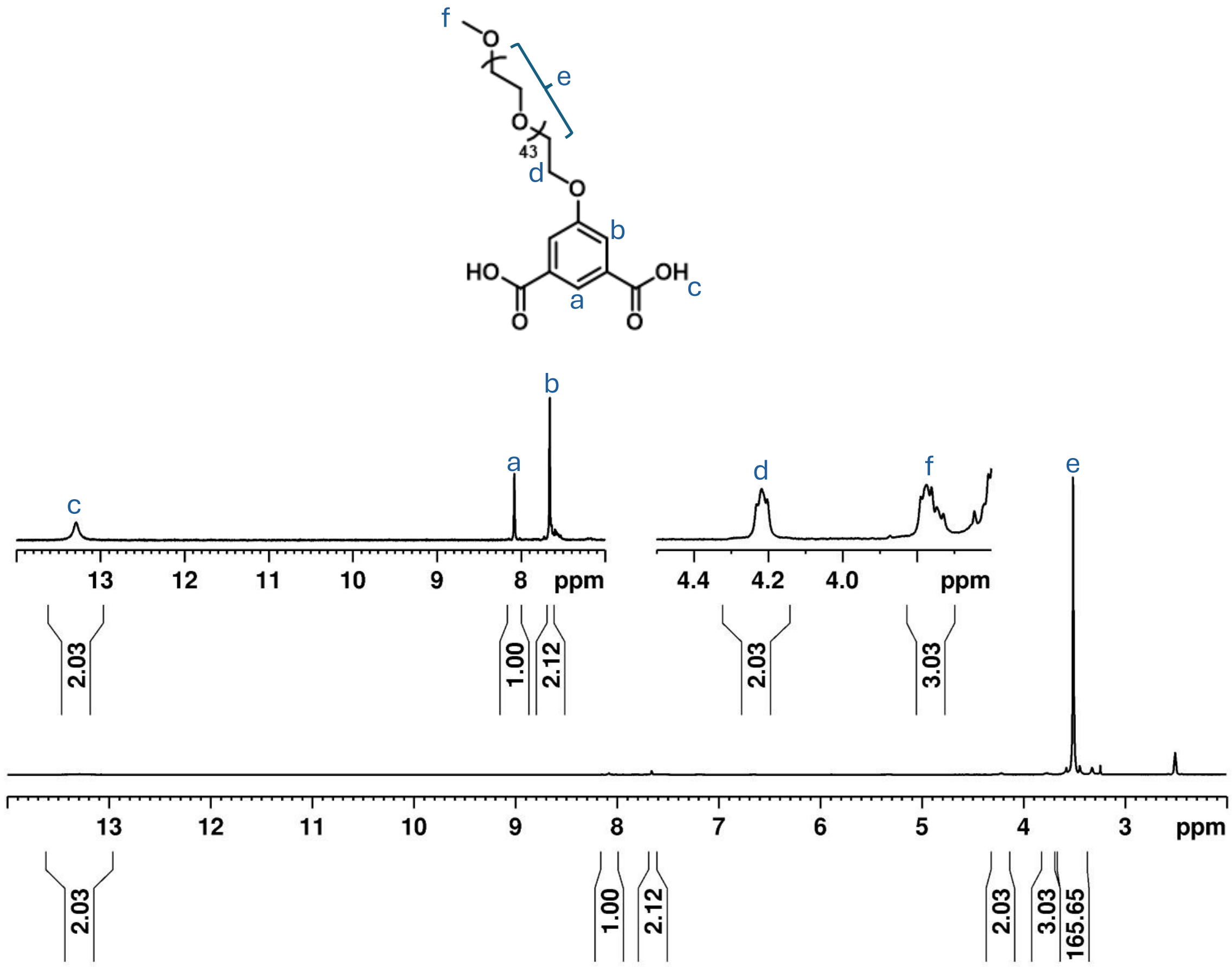


**Figure S6.** $^{1}$H-NMR spectrum (300 MHz, 25 ºC) of $H_2L1$ in DMSO-$d_6$.

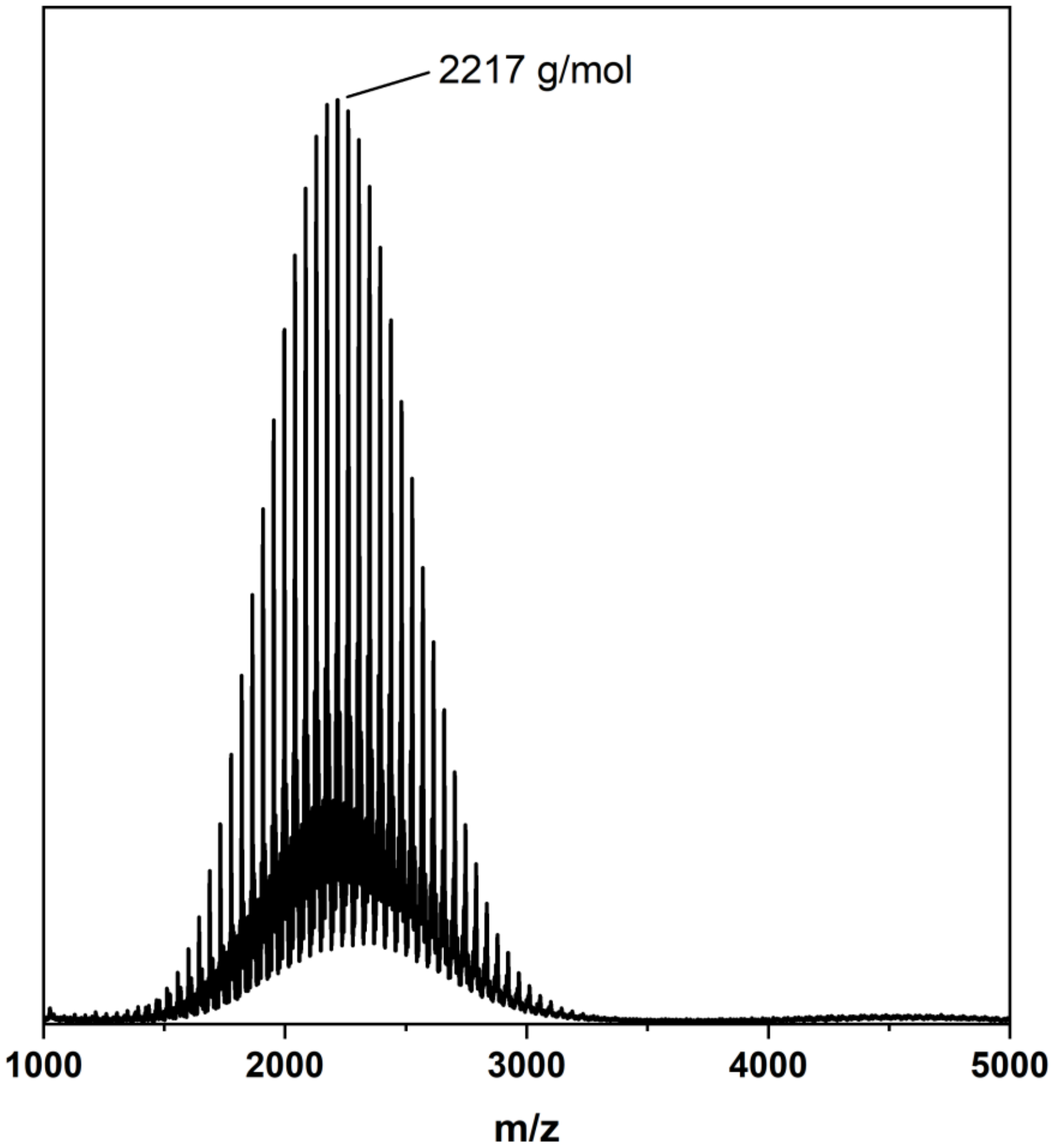


**Figure S7.** MALDI-TOF spectrum of $H_2L1$. The highlighted mass corresponds to the molecular formula of $[C_{95}O_{49}H_{180} + H^+]^+$ (expected m/z = 2105 ± 200 g·mol$^{-1}$; found m/z = 2217 g·mol$^{-1}$).

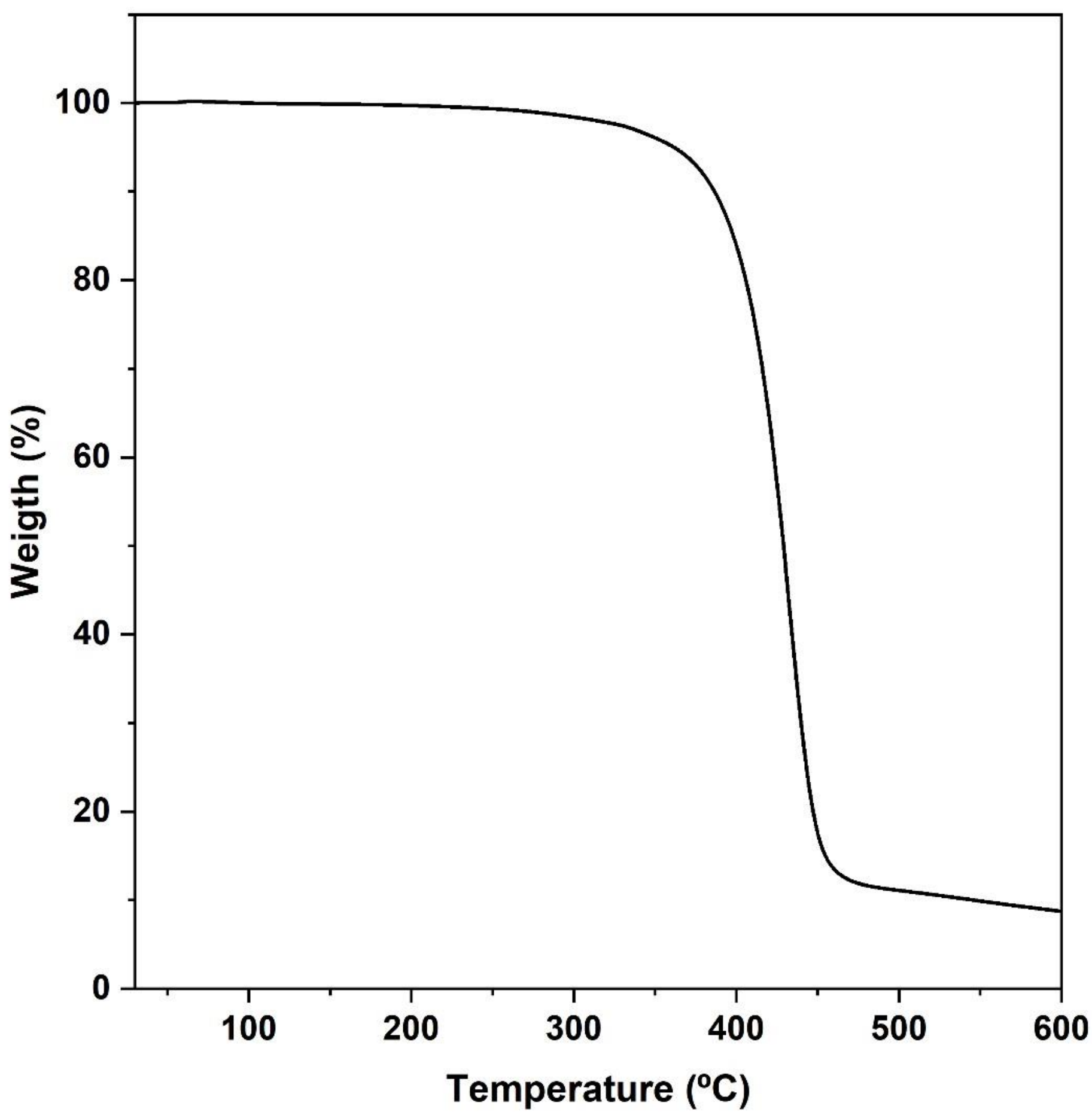


**Figure S8.** TGA curve of $H_2L1$, showing a decomposition temperature at ca. 300 ˚C.

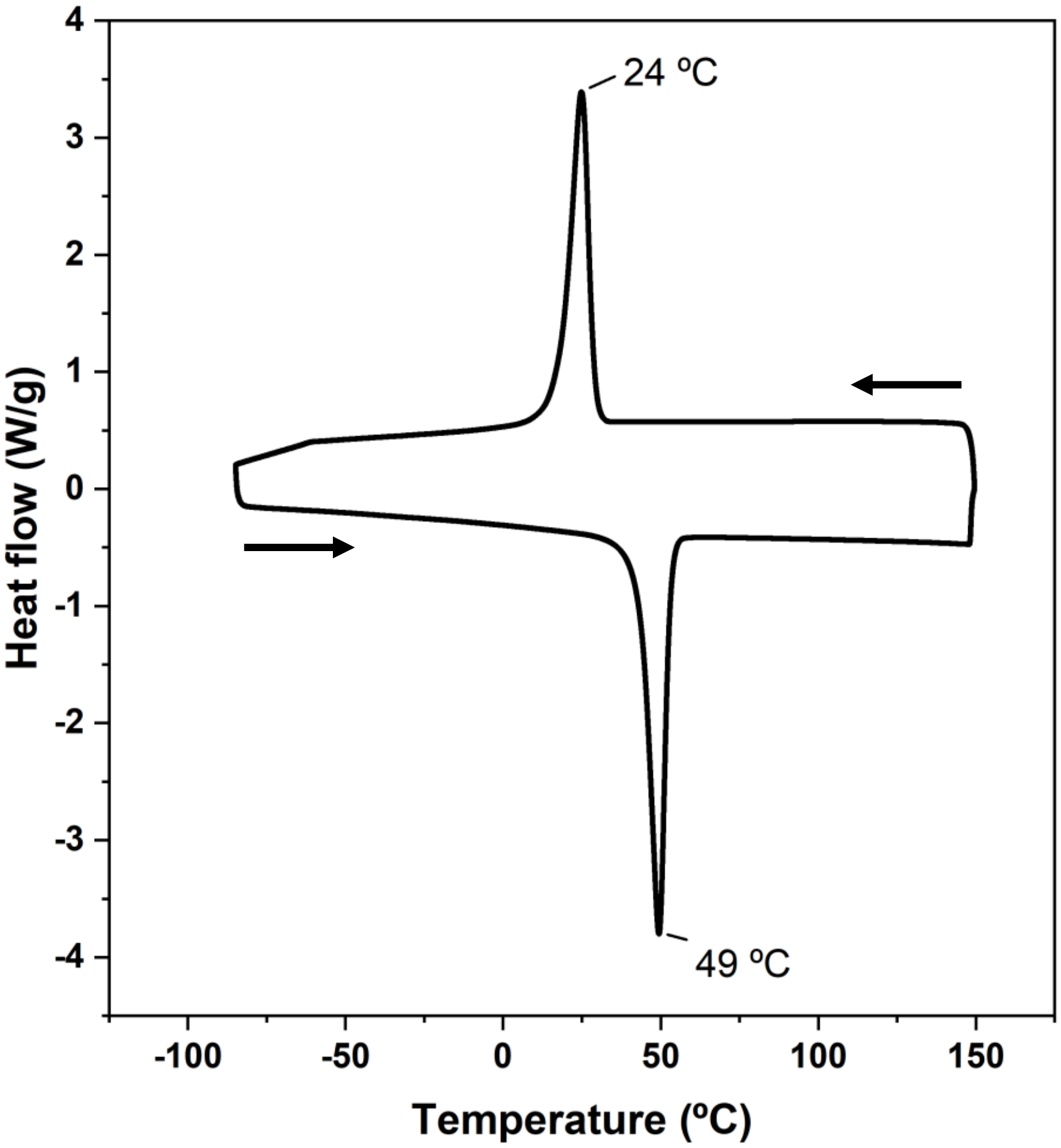


**Figure S9.** DSC curve of $H_2L1$.

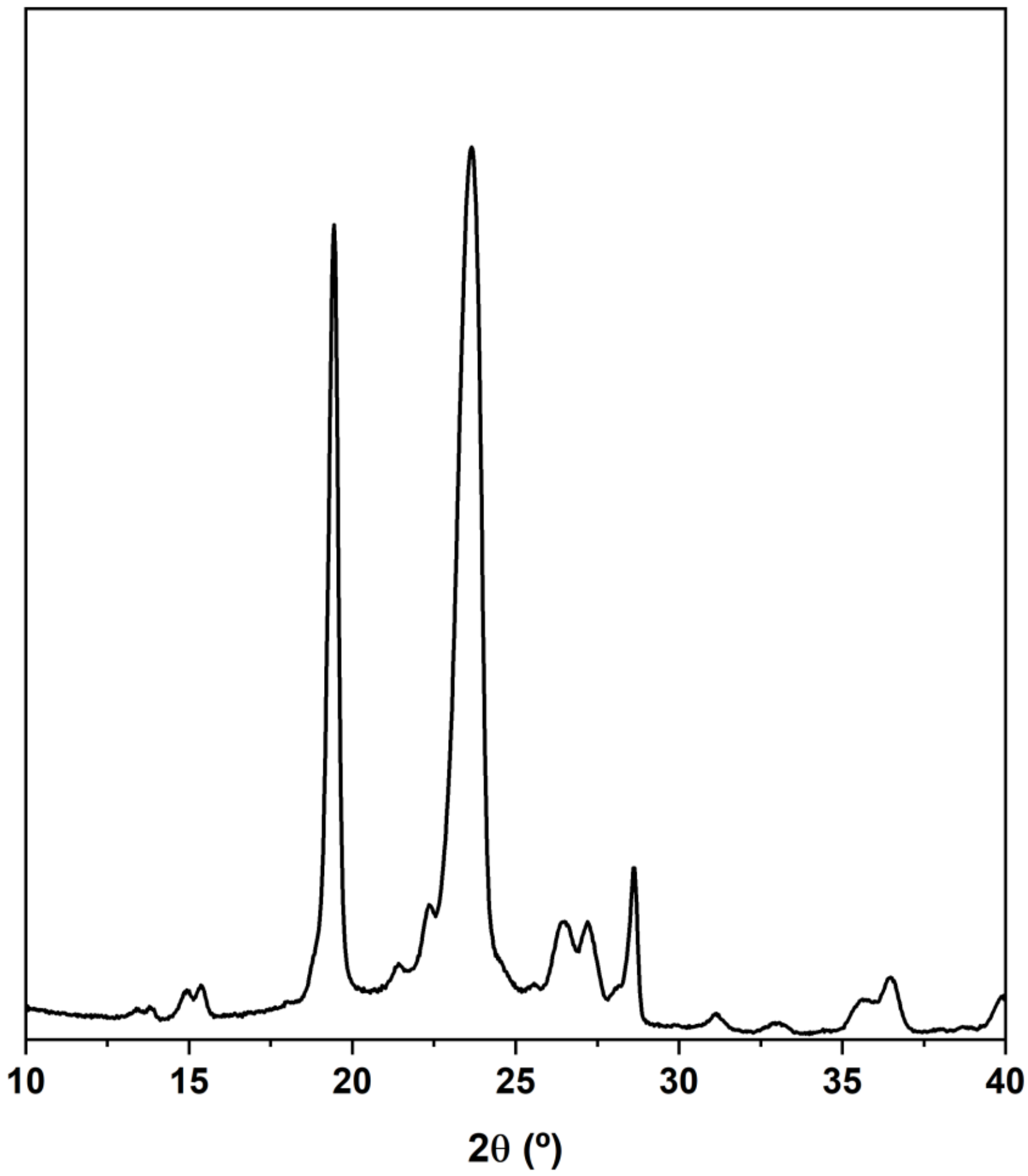


**Figure S10.** PXRD pattern of $H_2L1$.

### S3.3. Characterization Rh and Cu-BCN-5

### S3.3.1. Rh-BCN-5

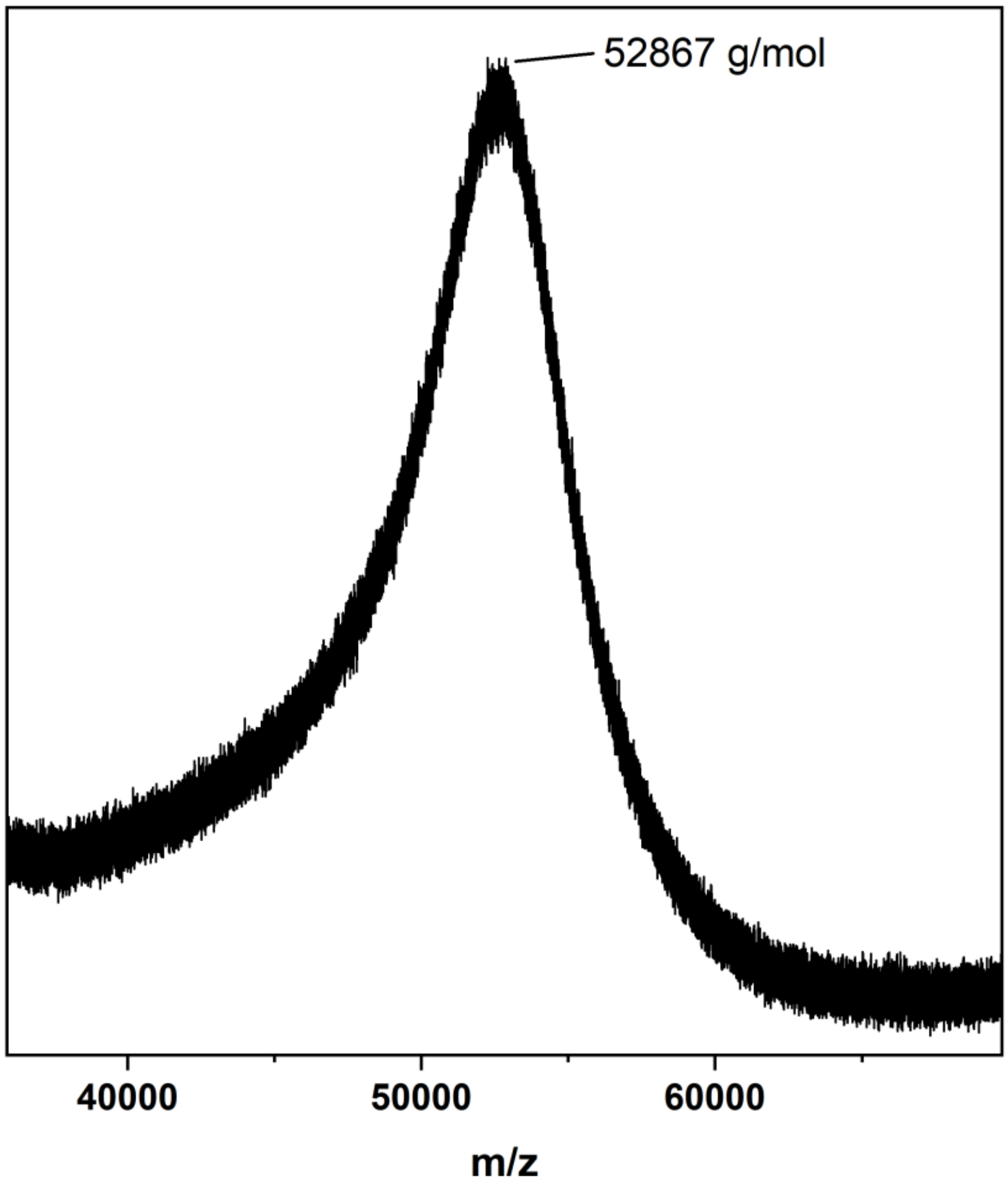


**Figure S11.** MALDI-TOF spectrum of Rh-BCN-5. The highlighted mass corresponds to the molecular formula of $[Rh_{24}(PEG\text{-}bdc)_{24} + H^{+}]^{+}$ (expected m/z = 52917 ± 4800 g·mol$^{-1}$; found m/z = 52867 g·mol$^{-1}$).

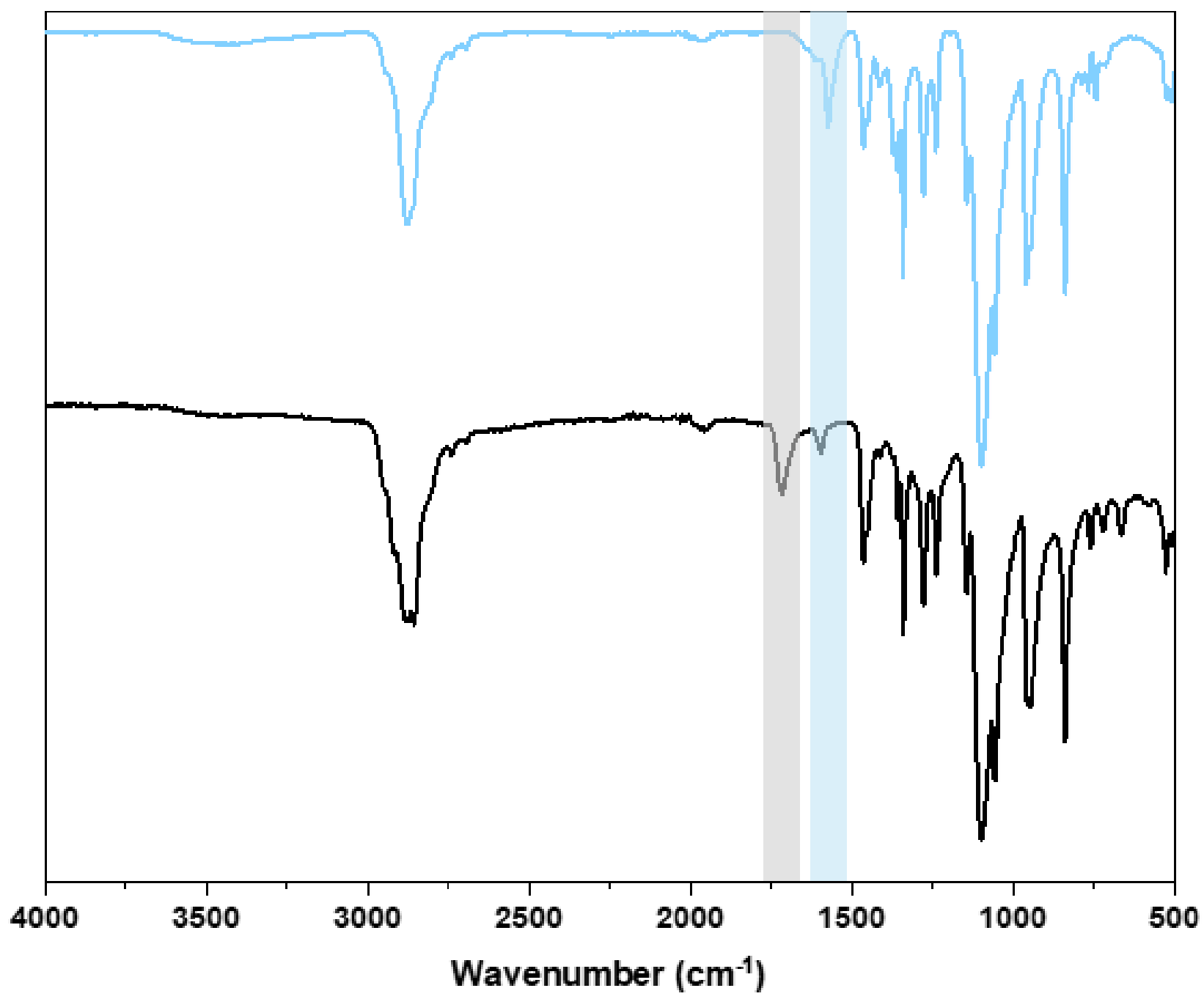


**Figure S12.** FTIR spectrum of $H_2L1$ (black, bottom) and Rh-BCN-5 (blue, top). The highlighted bands correspond to the asymmetric vibration of the C=O of the free carboxylic acid (light grey) and coordinated one (light blue).

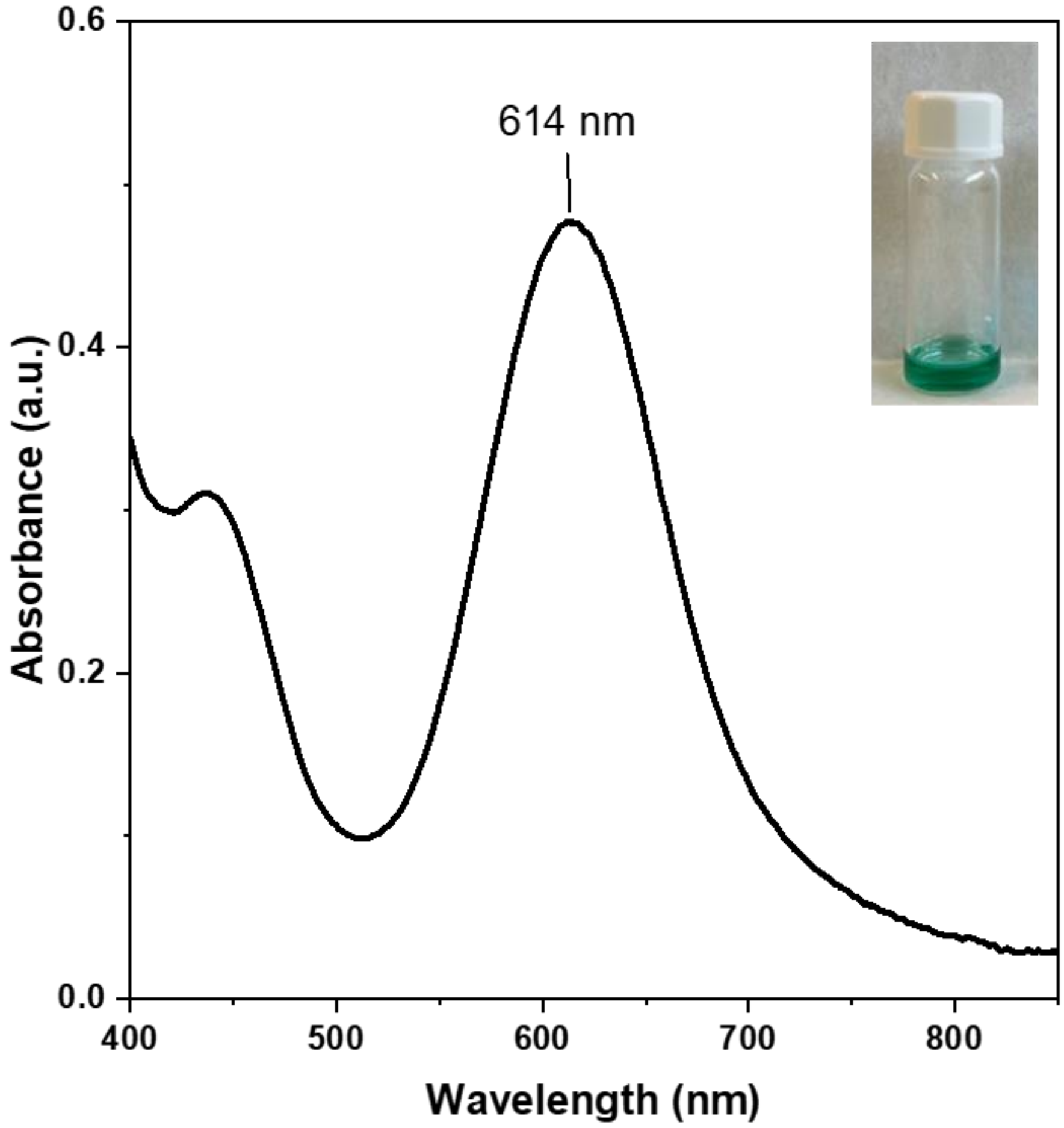


**Figure S13.** UV-Vis spectrum of Rh-BCN-5 in dichloromethane at a concentration of 3.5 mg·mL$^{-1}$.

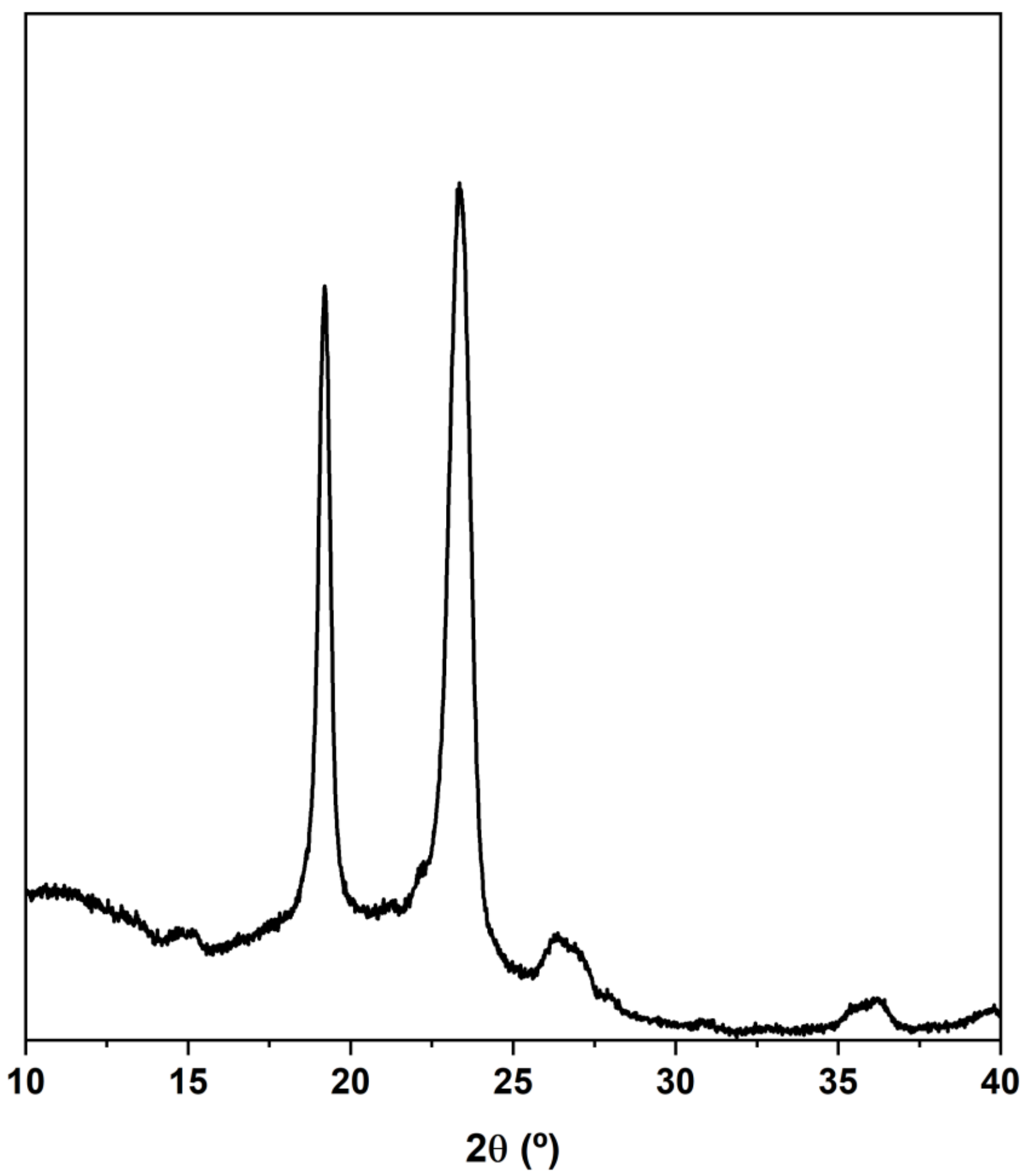


**Figure S14.** PXRD pattern of Rh-BCN-5 at room temperature.

### S3.3.2. Cu-BCN-5

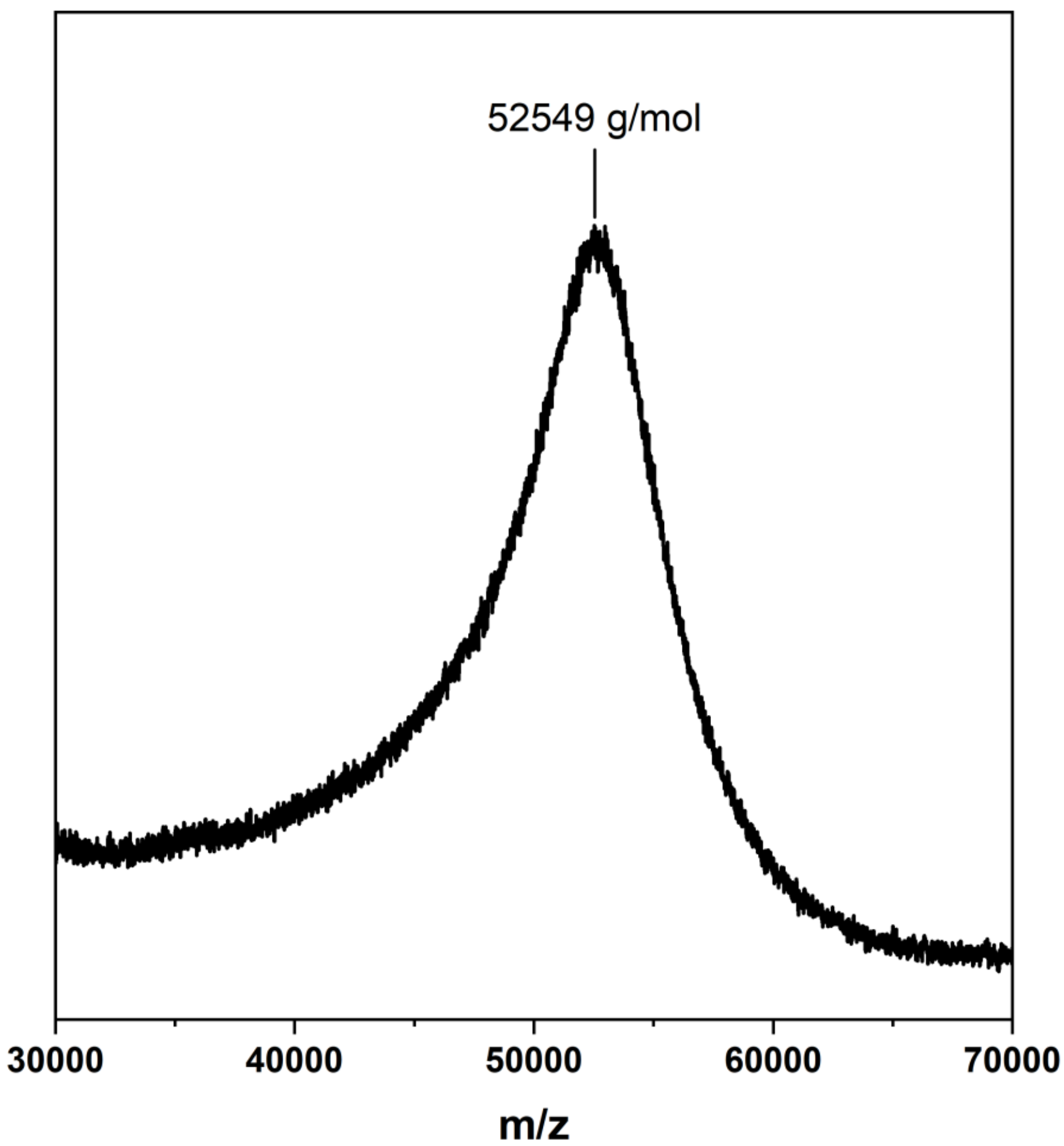


**Figure S15.** MALDI-TOF spectrum of Cu-BCN-5. The highlighted mass corresponds to the molecular formula of $[Cu_{24}(PEG\text{-}bdc)_{24} + H^+]^+$ (expected m/z = 51973 ± 4800 g·mol$^{-1}$; found m/z = 52549 g·mol$^{-1}$).

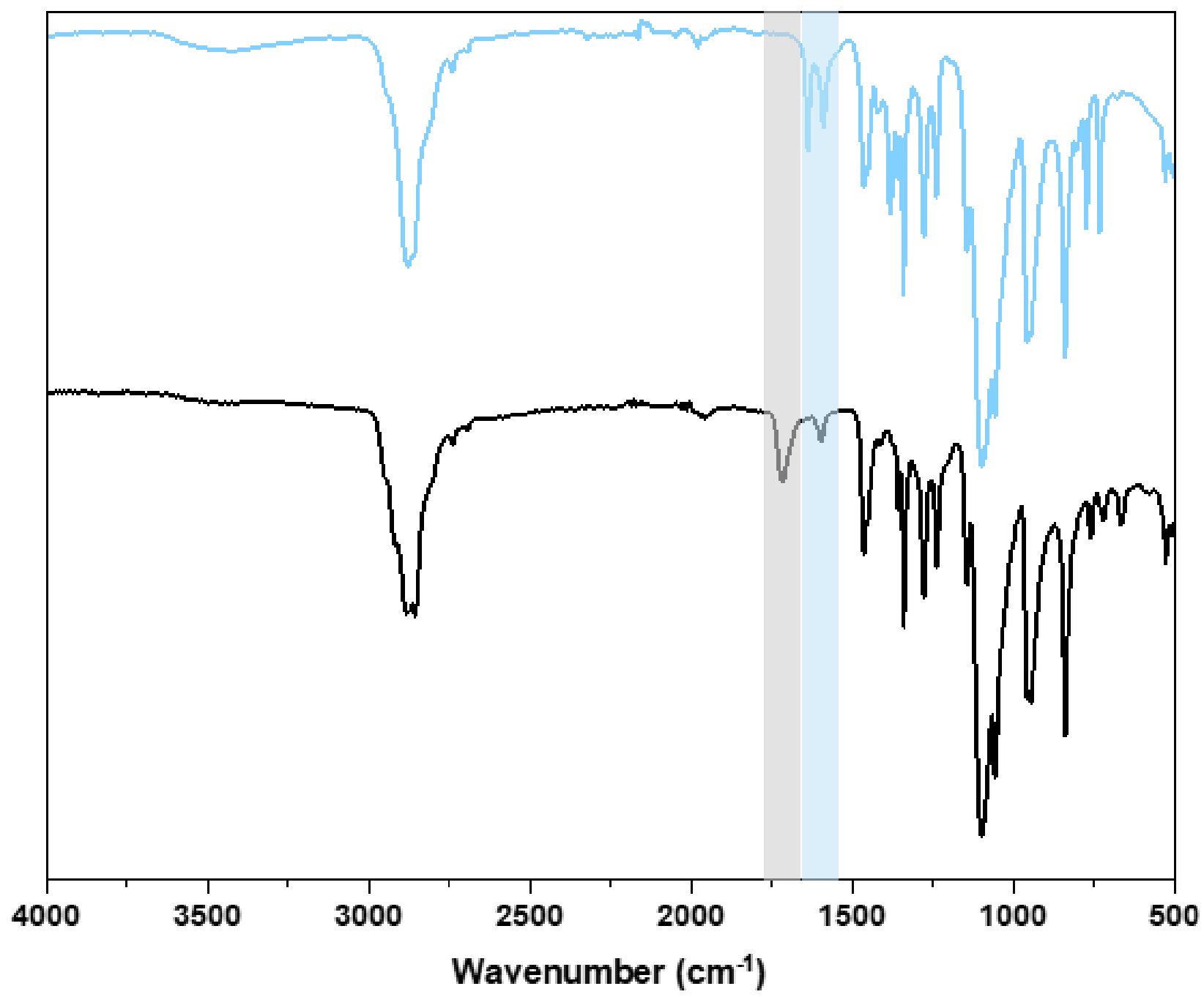


**Figure S16.** FTIR spectrum of $H_2L1$ (black, bottom) and Cu-BCN-5 (blue, top). The highlighted bands correspond to the asymmetric vibration of the C=O of the free carboxylic acid (light grey) and coordinated one (light blue).

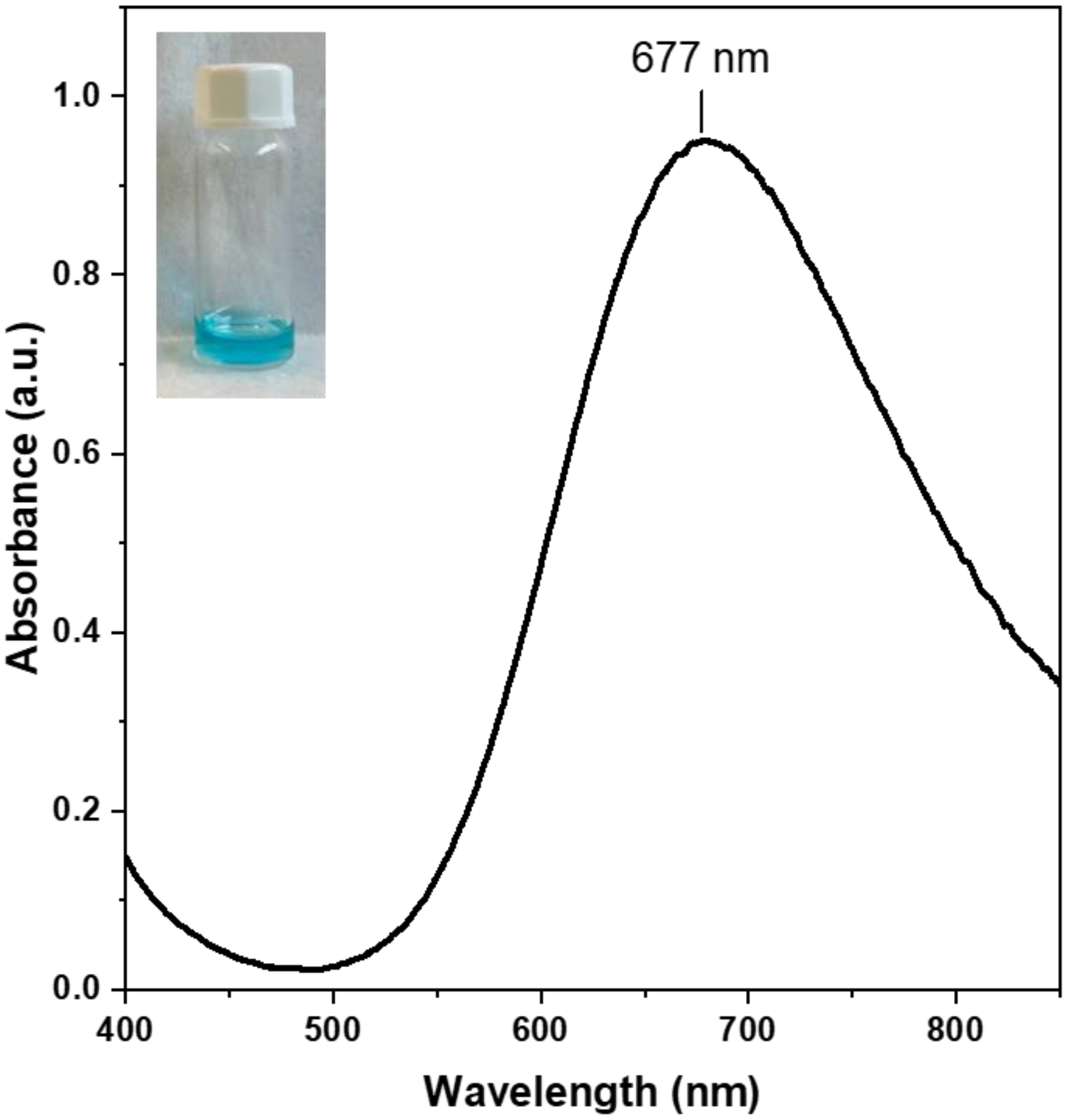


**Figure S17.** UV-Vis spectrum of Cu-BCN-5 dissolved in dichloromethane at a concentration of 3.5 $mg \cdot mL^{-1}$.

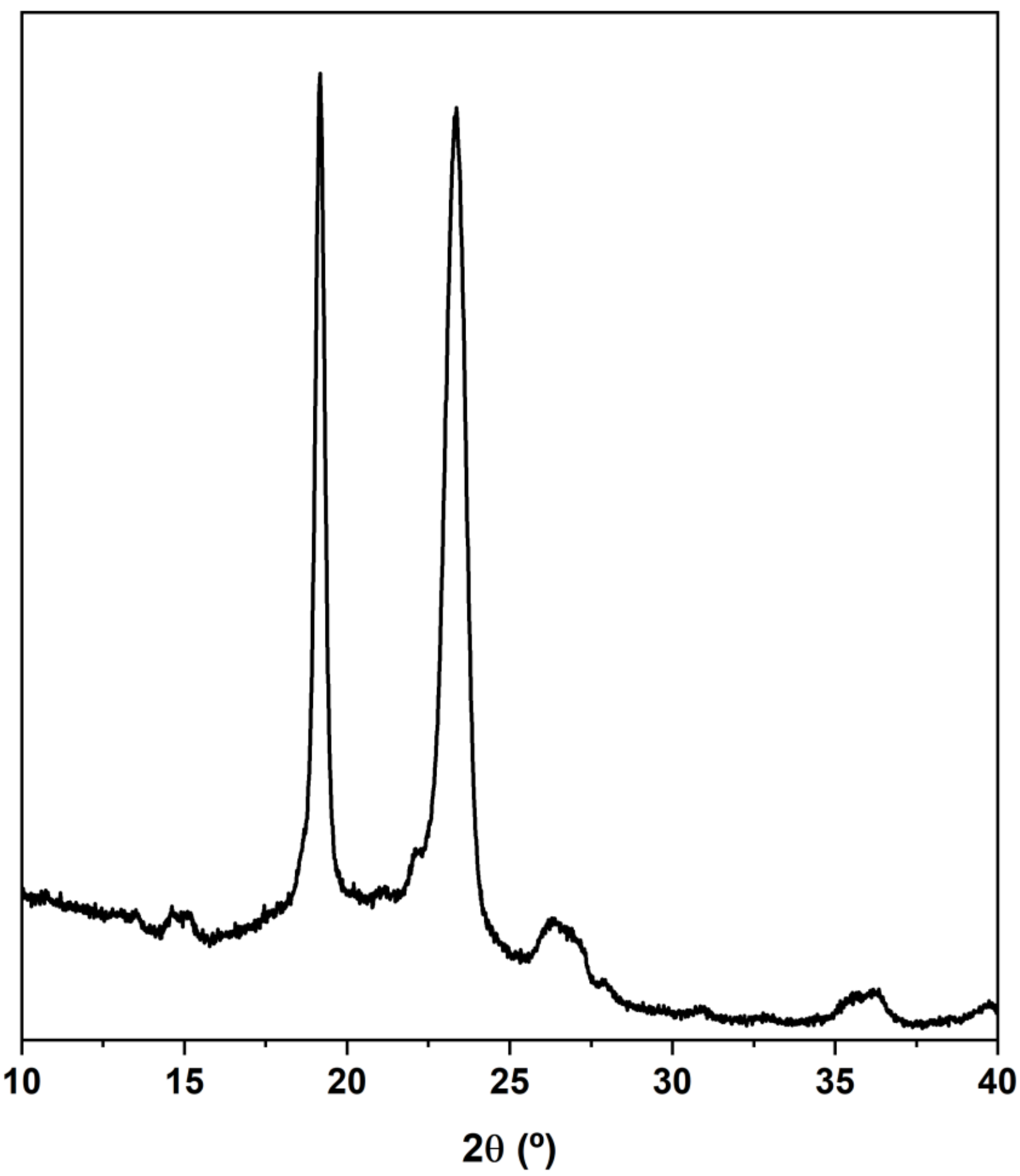


**Figure S18.** PXRD pattern of Cu-BCN-5 at room temperature.

## S3.4. Thermal properties and stability

### S3.4.1. Rh-BCN-5

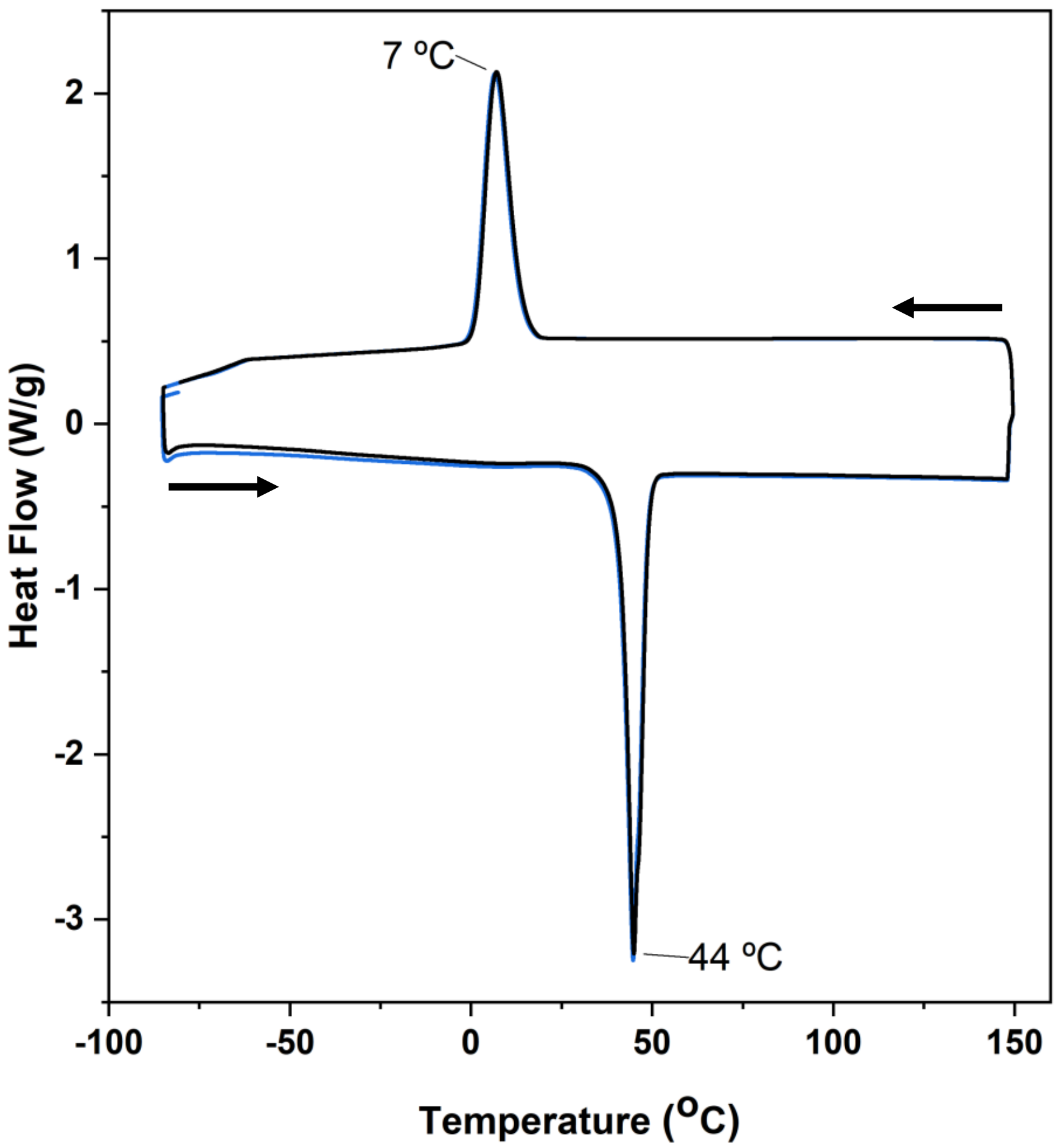


**Figure S19.** DSC curves of Rh-BCN-5, showing two consecutive runs.

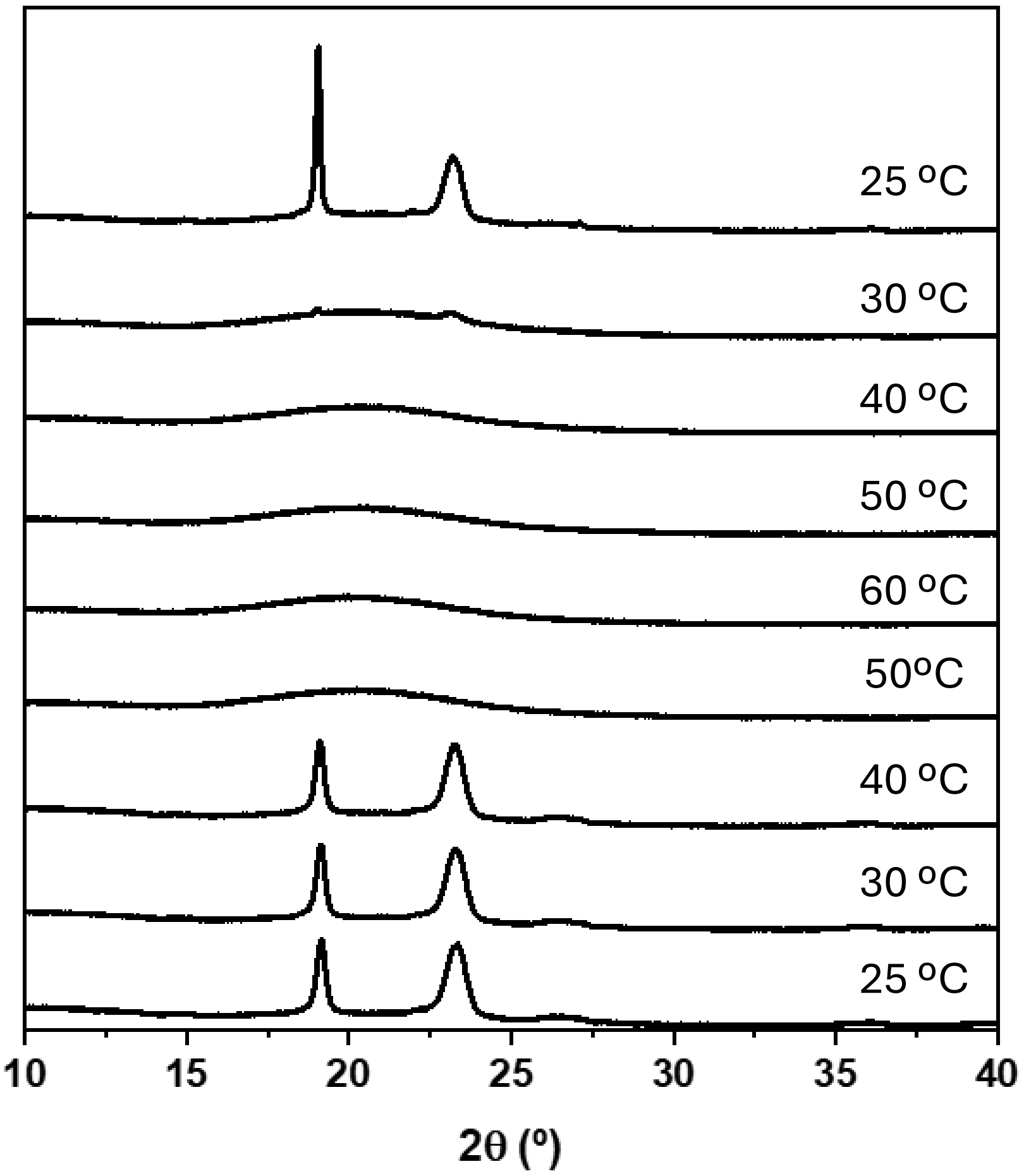


**Figure S20.** Variable-temperature PXRD patterns of Rh-BCN-5.

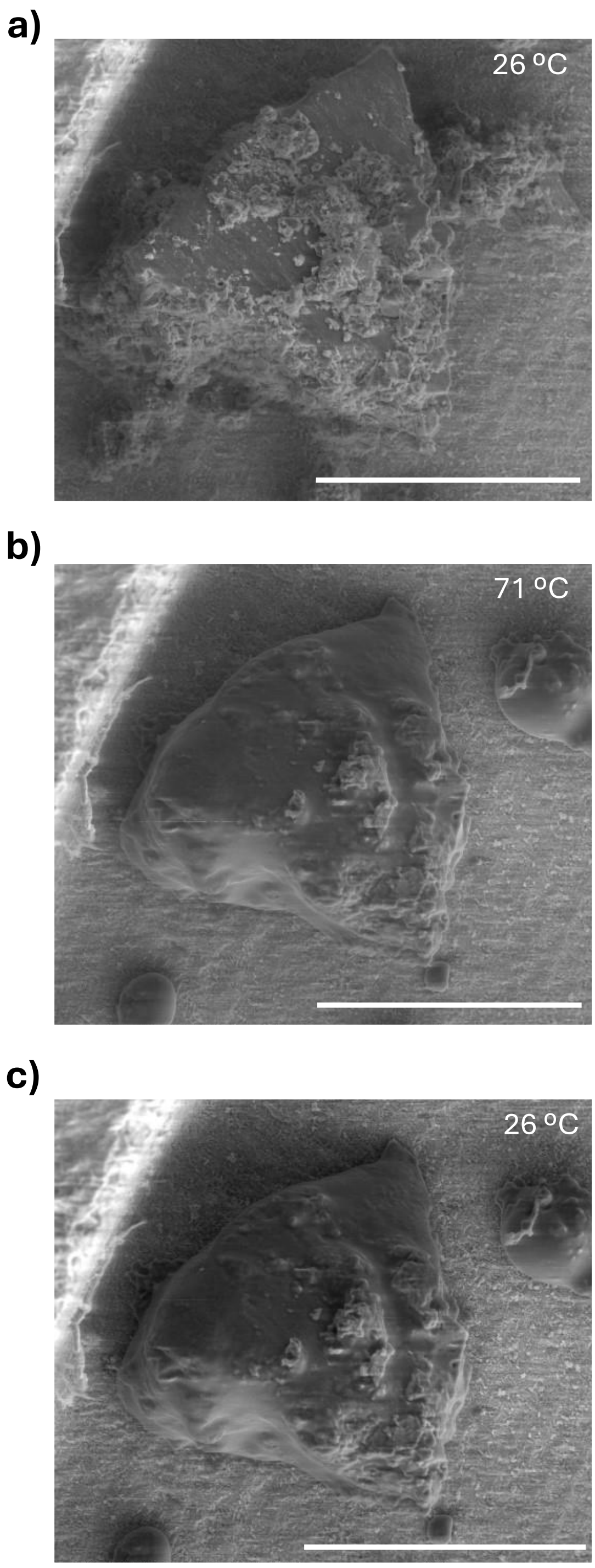


**Figure S21.** Variable-temperature FE-SEM images of semicrystalline Rh-BCN-5: (a) as-prepared sample, (b) after *in situ* heating at 71 °C, and (c) after cooling back to room temperature. Scale bars: 500 µm.

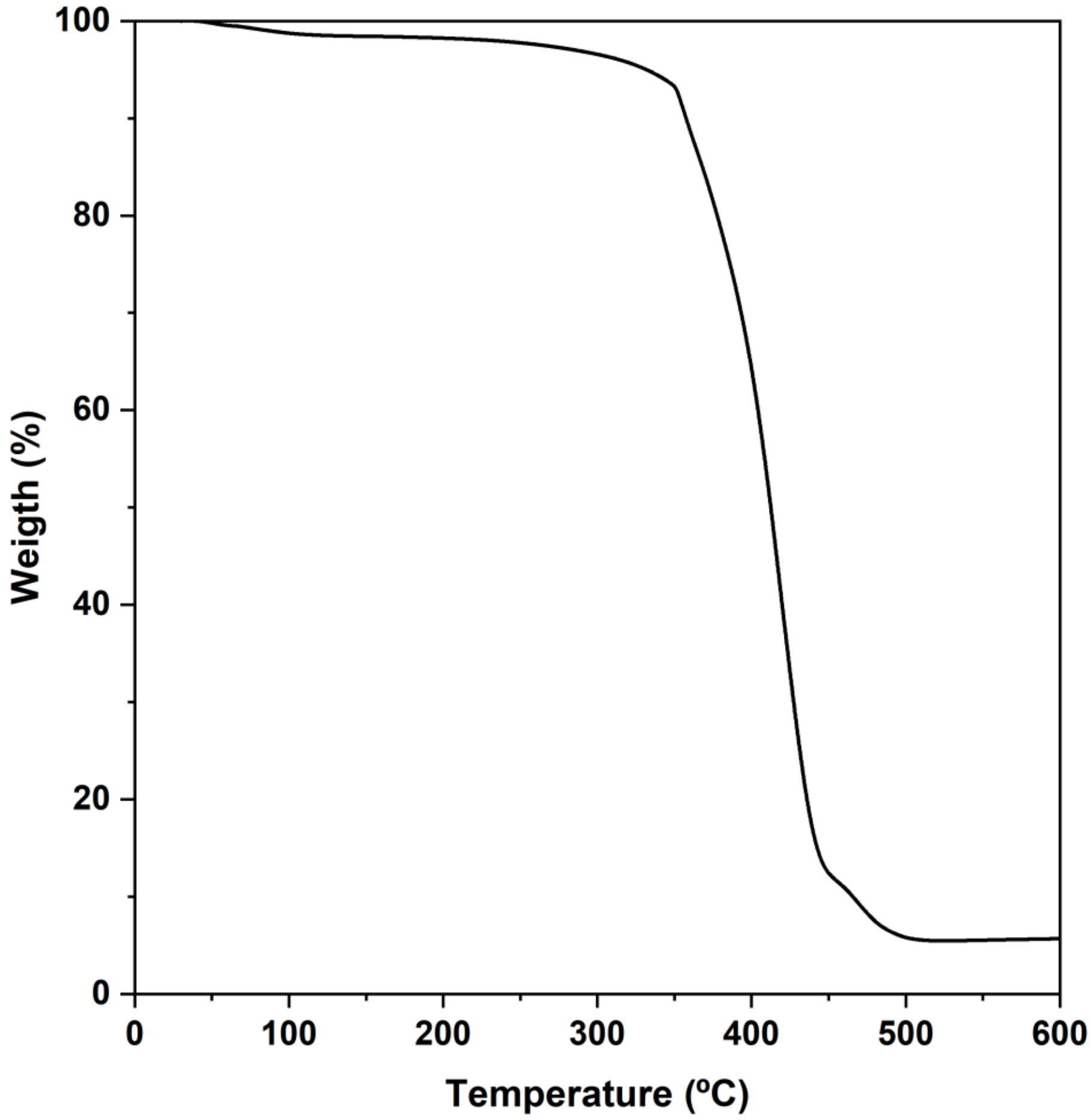


**Figure S22.** TGA curve of Rh-BCN-5 showing a decomposition temperature at ca. 335 °C

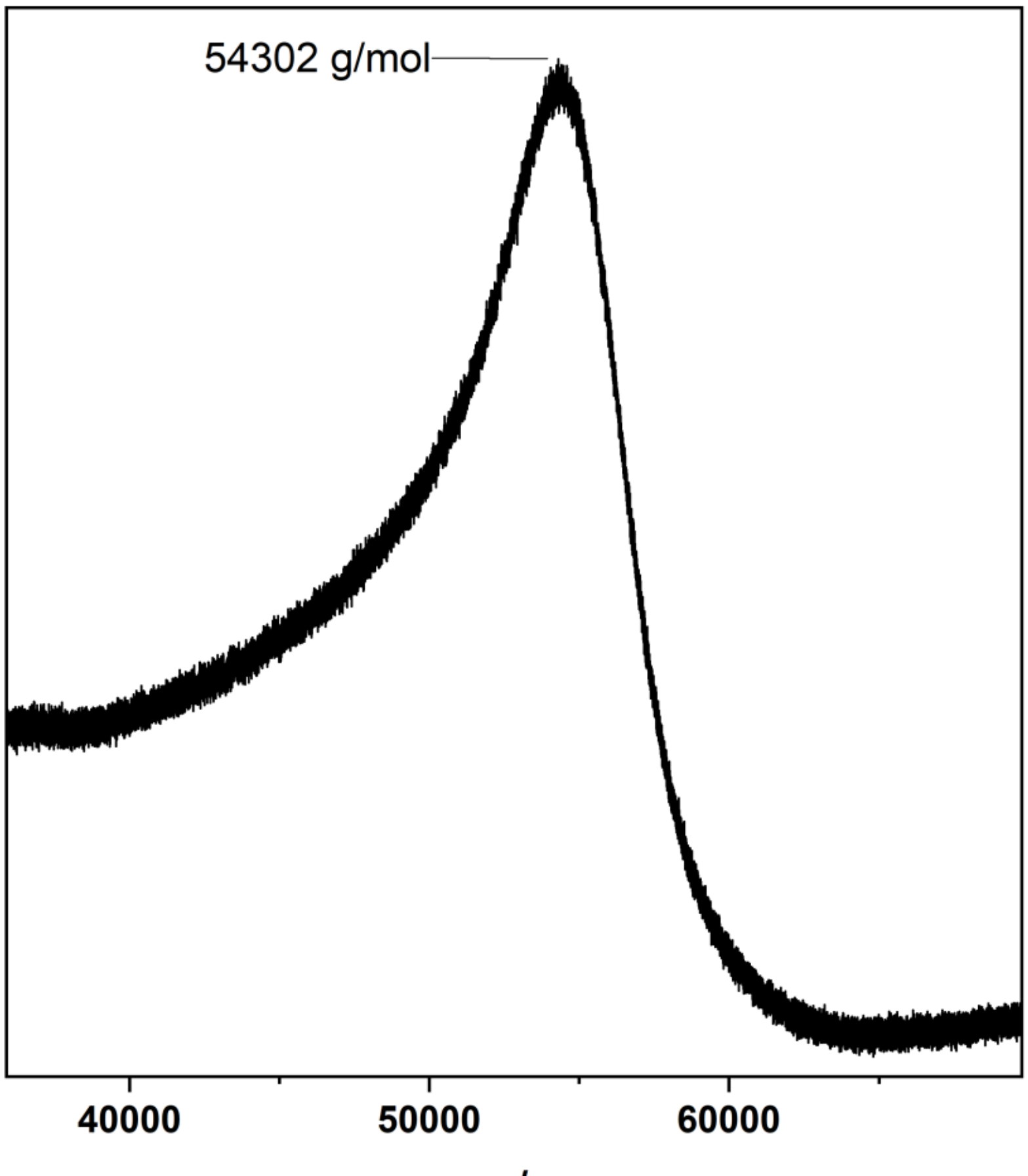


**Figure S23.** MALDI-TOF spectrum of Rh-BCN-5 after melting-cooling cycle. The highlighted mass corresponds to the molecular formula of $[Rh_{24}(PEG\text{-}bdc)_{24} + H^+]^+$ (expected m/z = 52917 ± 4800 g·mol$^{-1}$; found m/z = 54302 g·mol$^{-1}$).

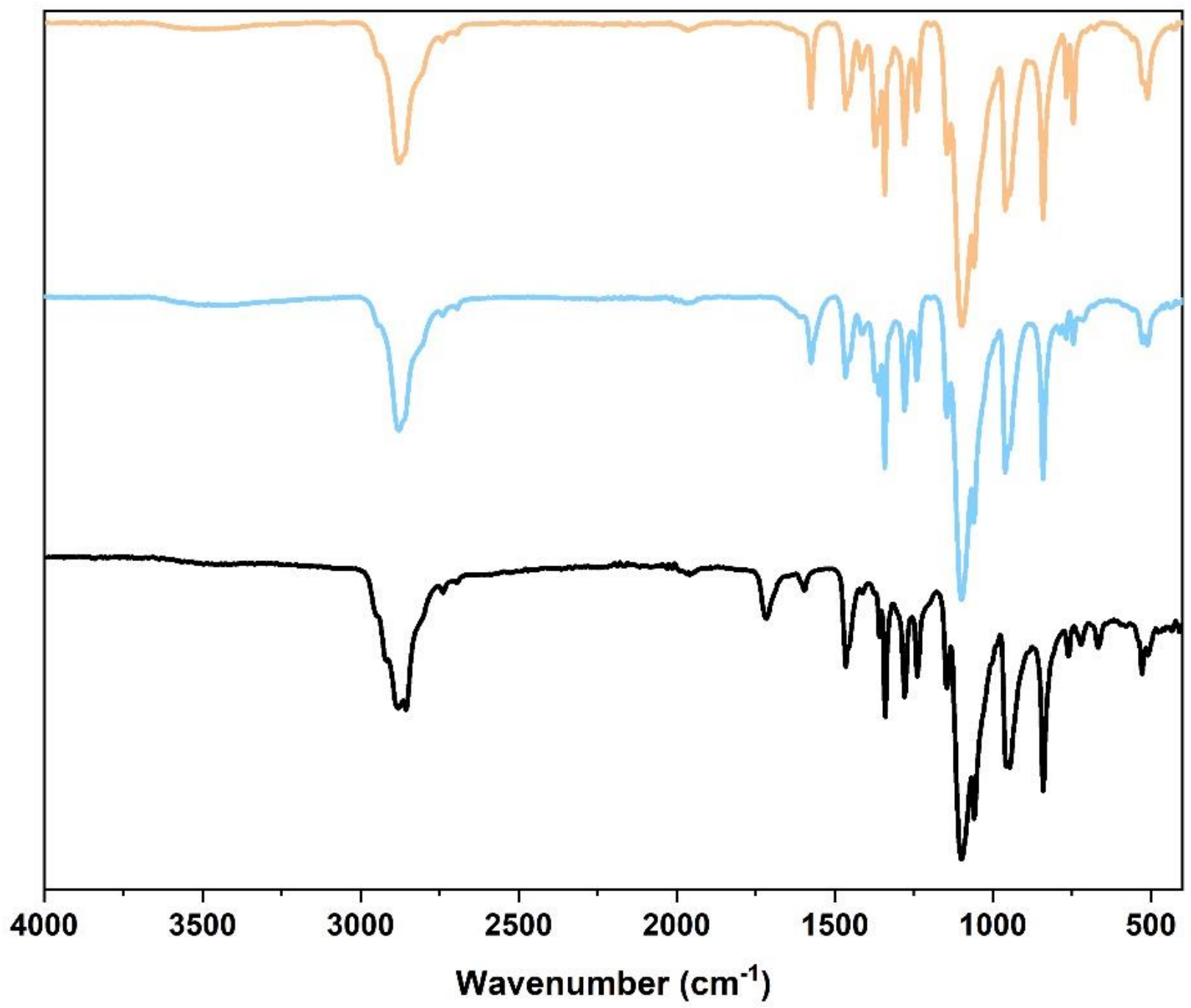


**Figure S24.** FTIR spectra of $H_2L1$ (black, bottom), as-made Rh-BCN-5 (blue, middle) and Rh-BCN-5 after a melting-cooling cycle (orange, top).

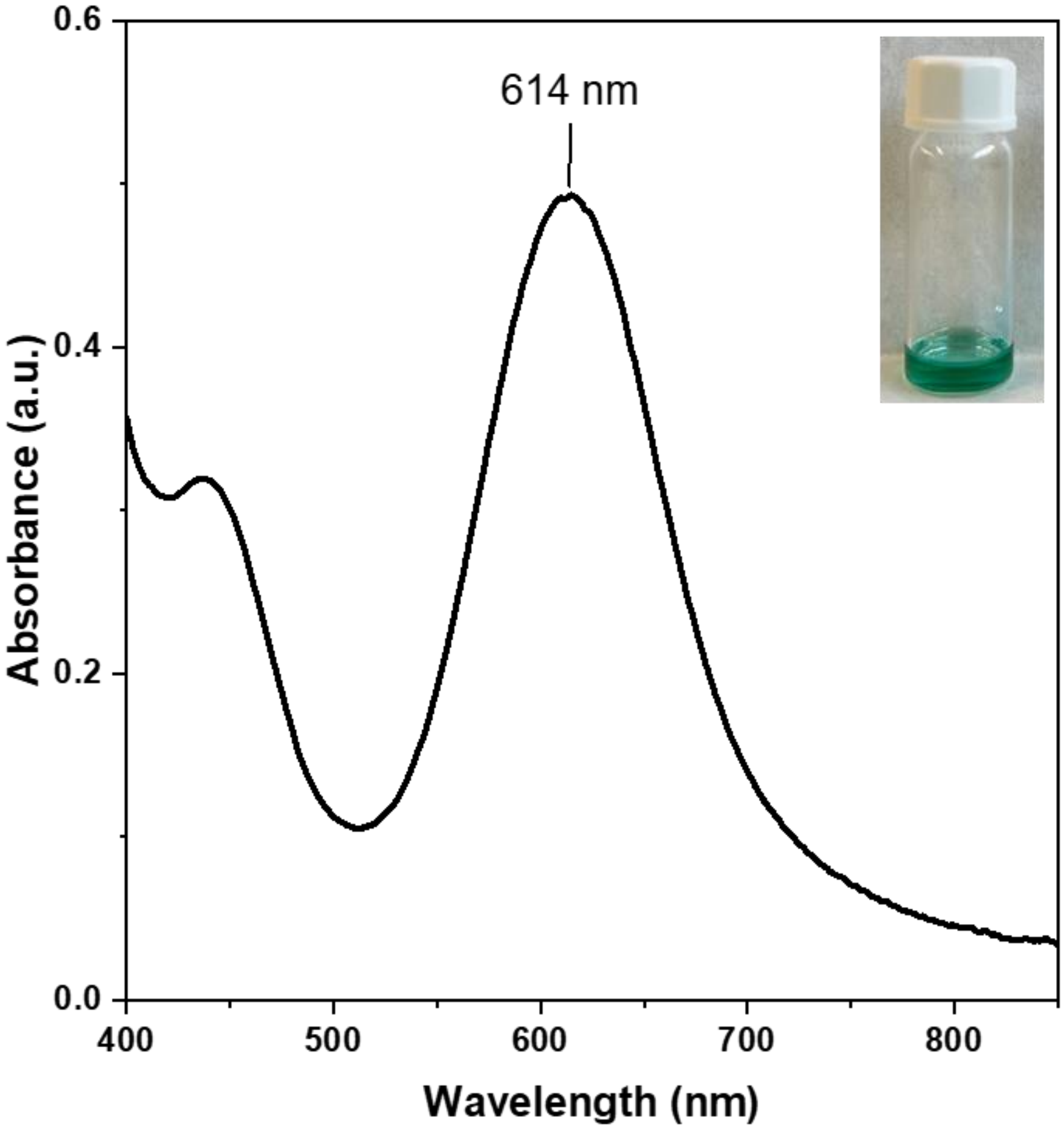


**Figure S25.** UV-Vis spectrum of Rh-BCN-5 after a melting-cooling cycle dissolved in dichloromethane at a concentration of 3.5 mg·mL$^{-1}$.

**S3.4.2. Cu-BCN-5**

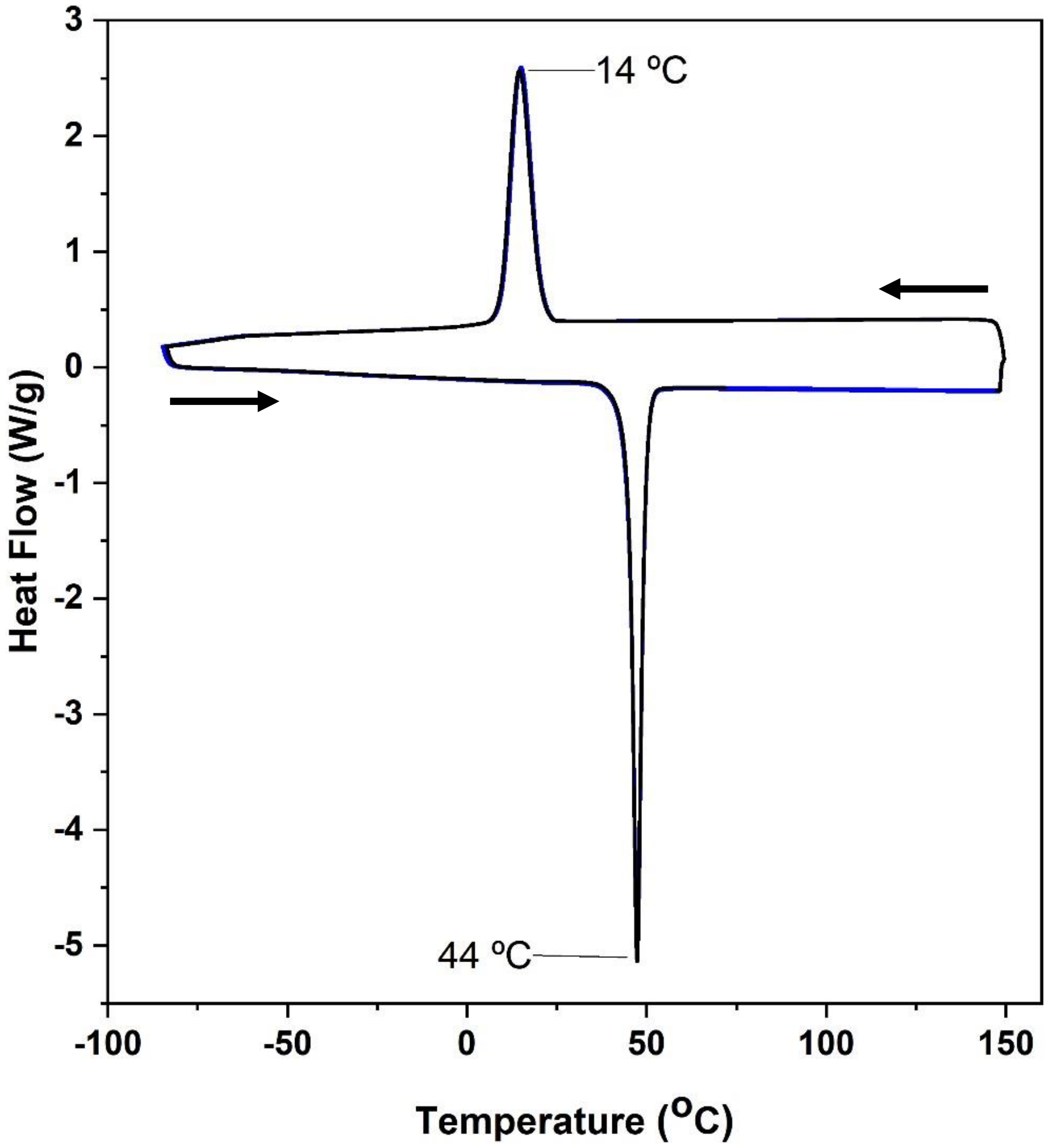


**Figure S26.** DSC curves of Cu-BCN-5, showing two consecutive runs.

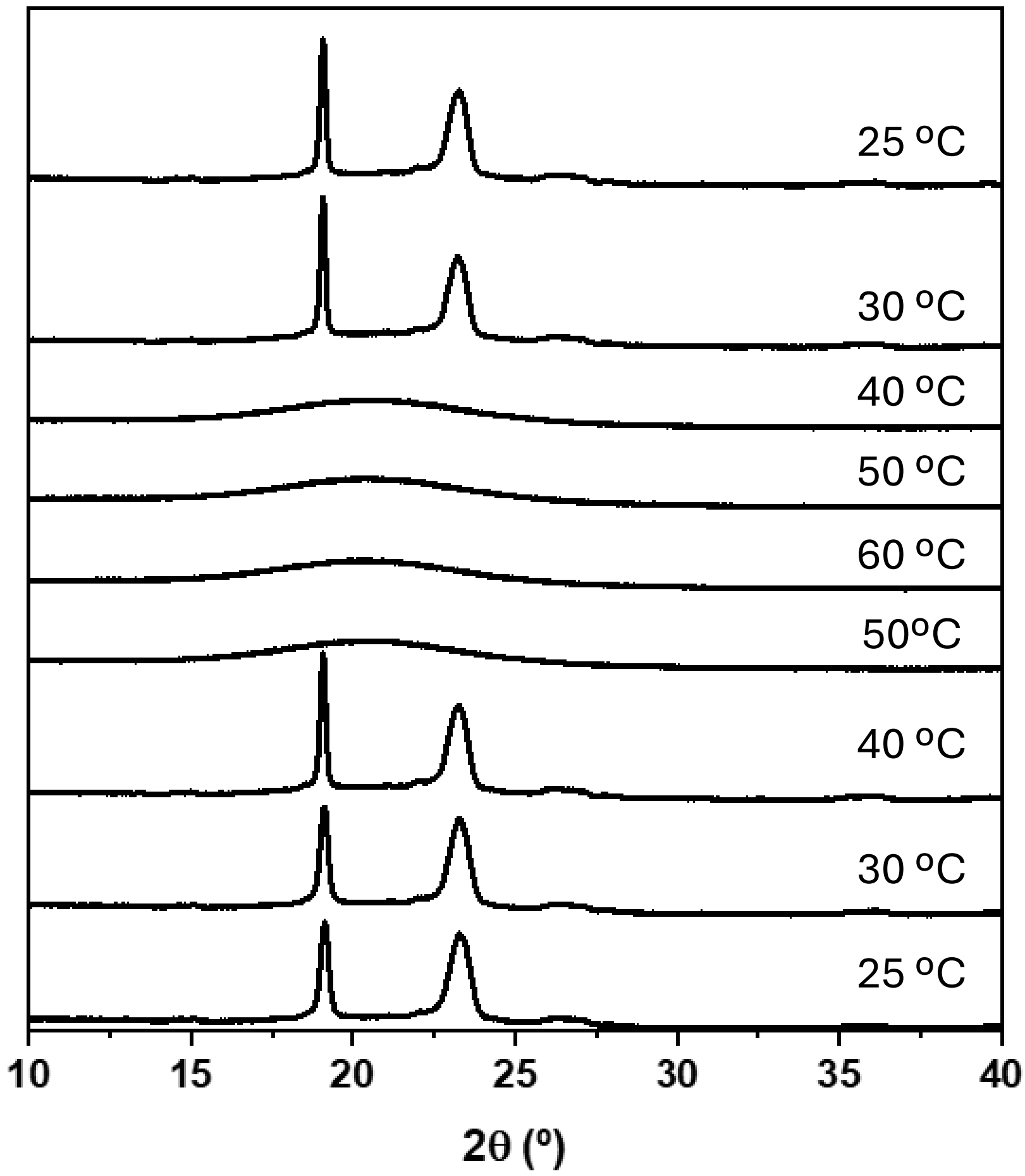


**Figure S27.** Variable-temperature PXRD patterns of Cu-BCN-5.

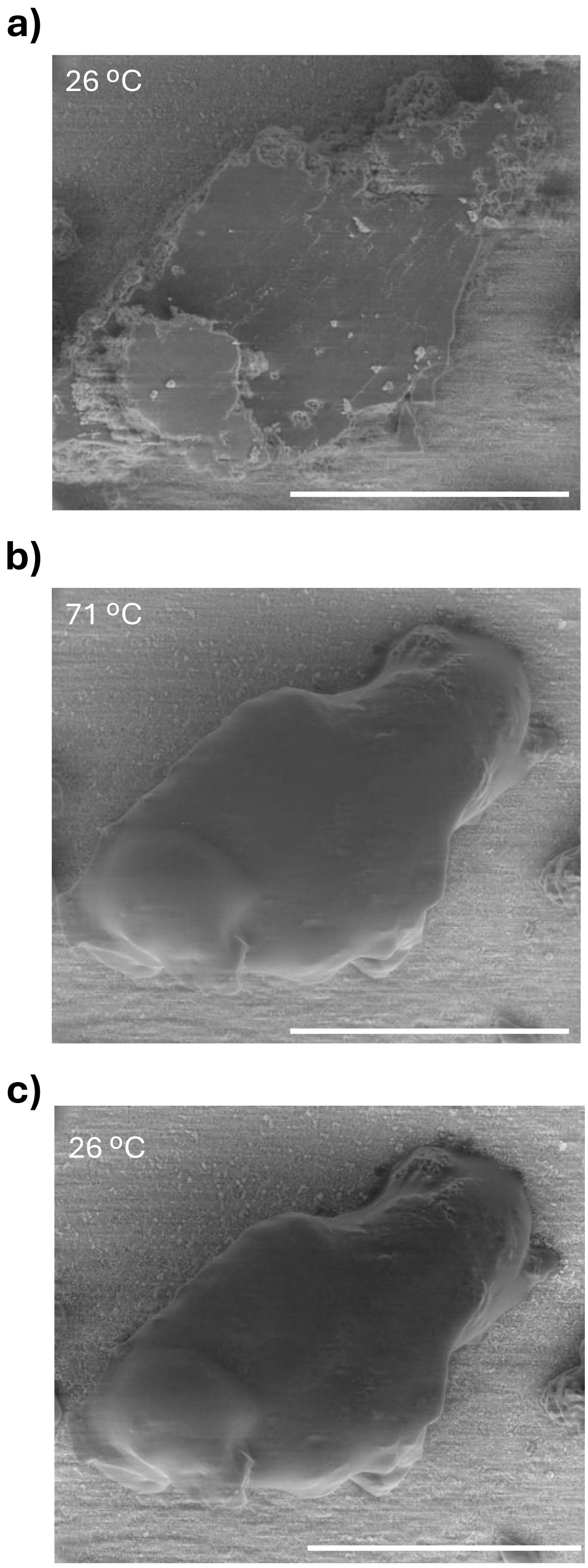


**Figure S28.** Variable-temperature FE-SEM images of semicrystalline Cu-BCN-5: (a) as-prepared sample, (b) after *in situ* heating at 71 °C, and (c) after cooling back to room temperature. Scale bars: 500 µm.

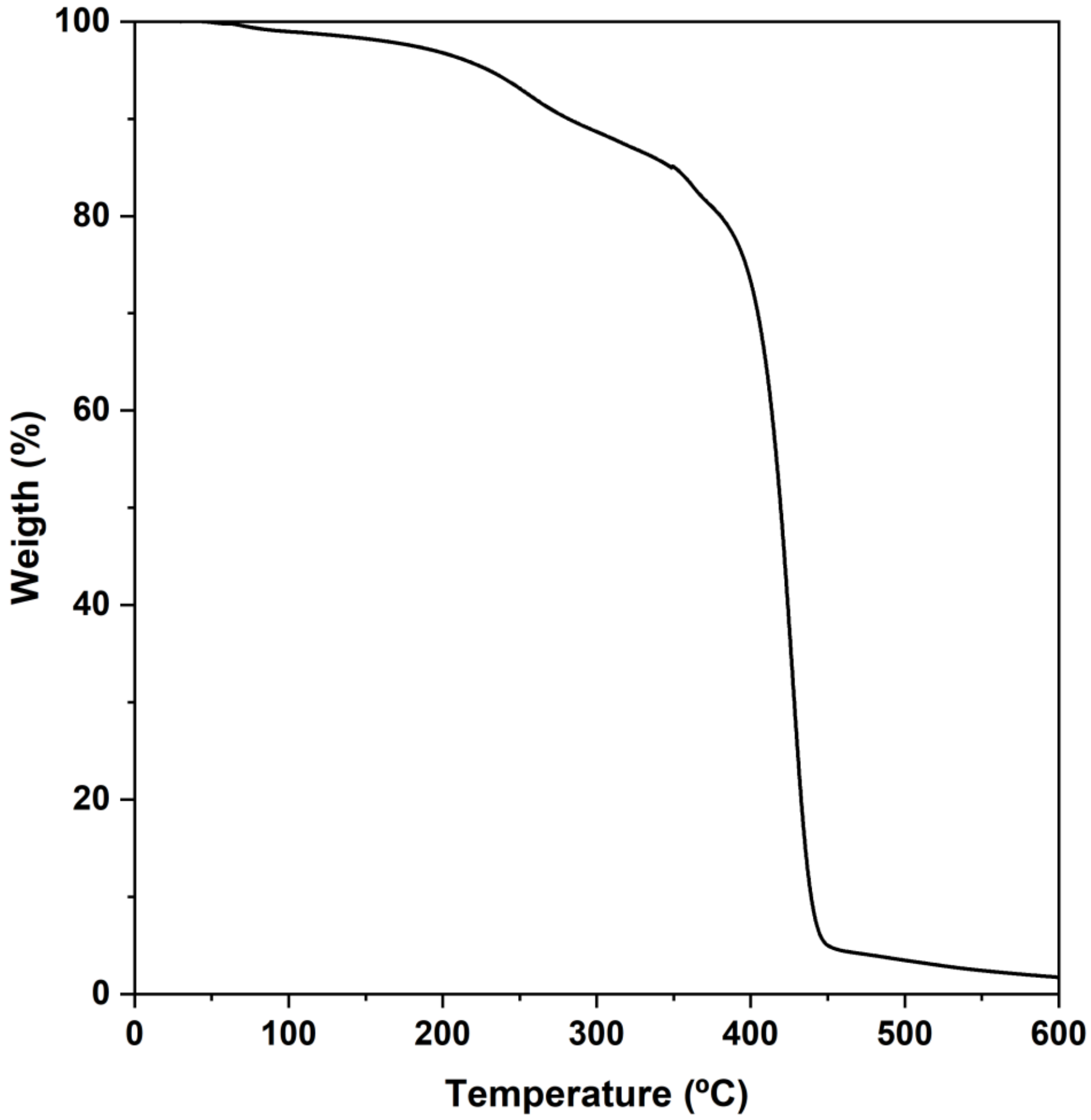


**Figure S29.** TGA curve of Cu-BCN-5 showing a decomposition temperature of ca. 250 °C.

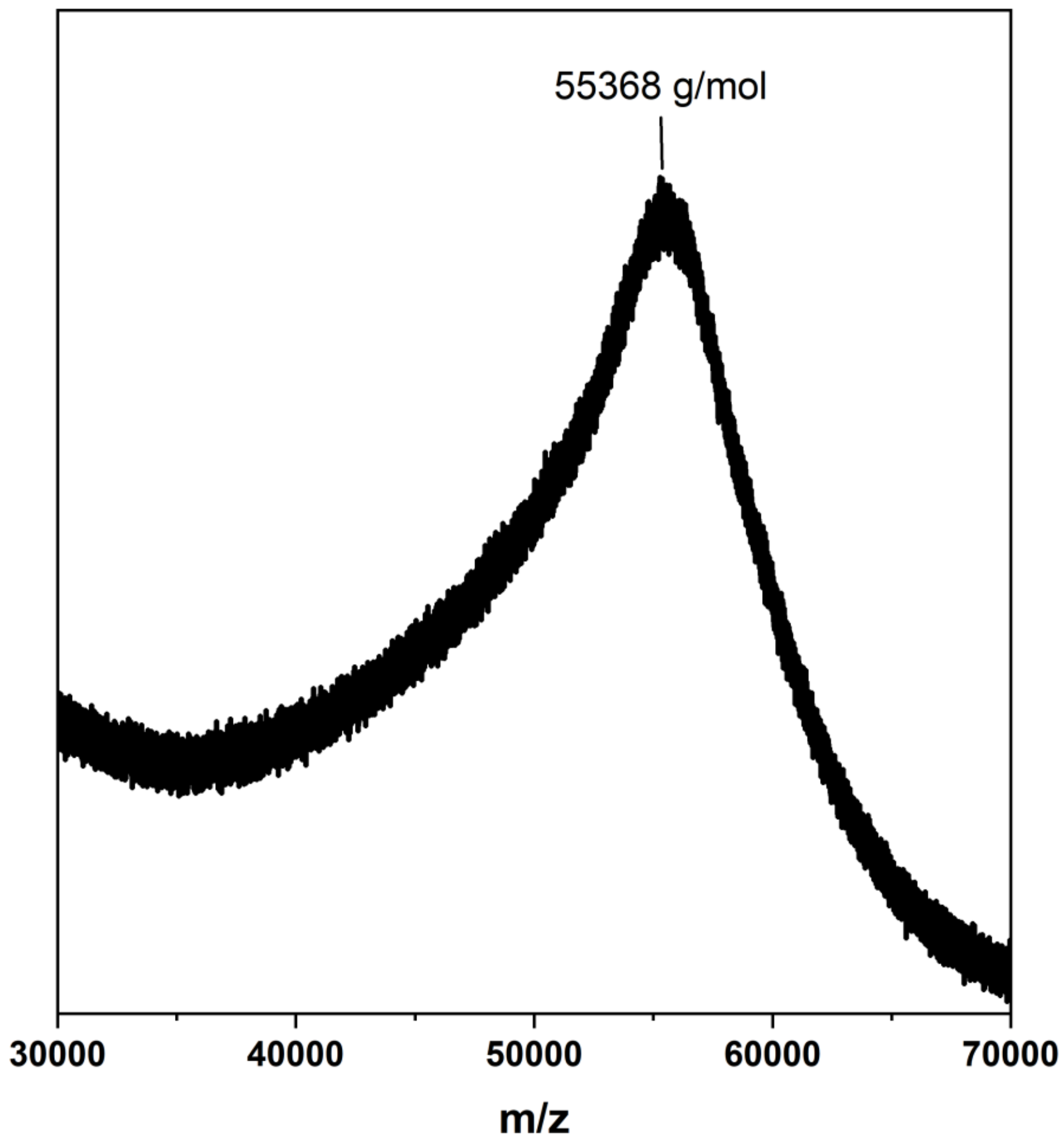


**Figure S30.** MALDI-TOF spectrum of Cu-BCN-5 after a melting-cooling cycle. The highlighted mass corresponds to the molecular formula of $[Cu_{24}(PEG\text{-}bdc)_{24} + H^+]^+$ (expected m/z = 51973 ± 4800 $g{\cdot}mol^{-1}$; found m/z = 55368 $g{\cdot}mol^{-1}$).

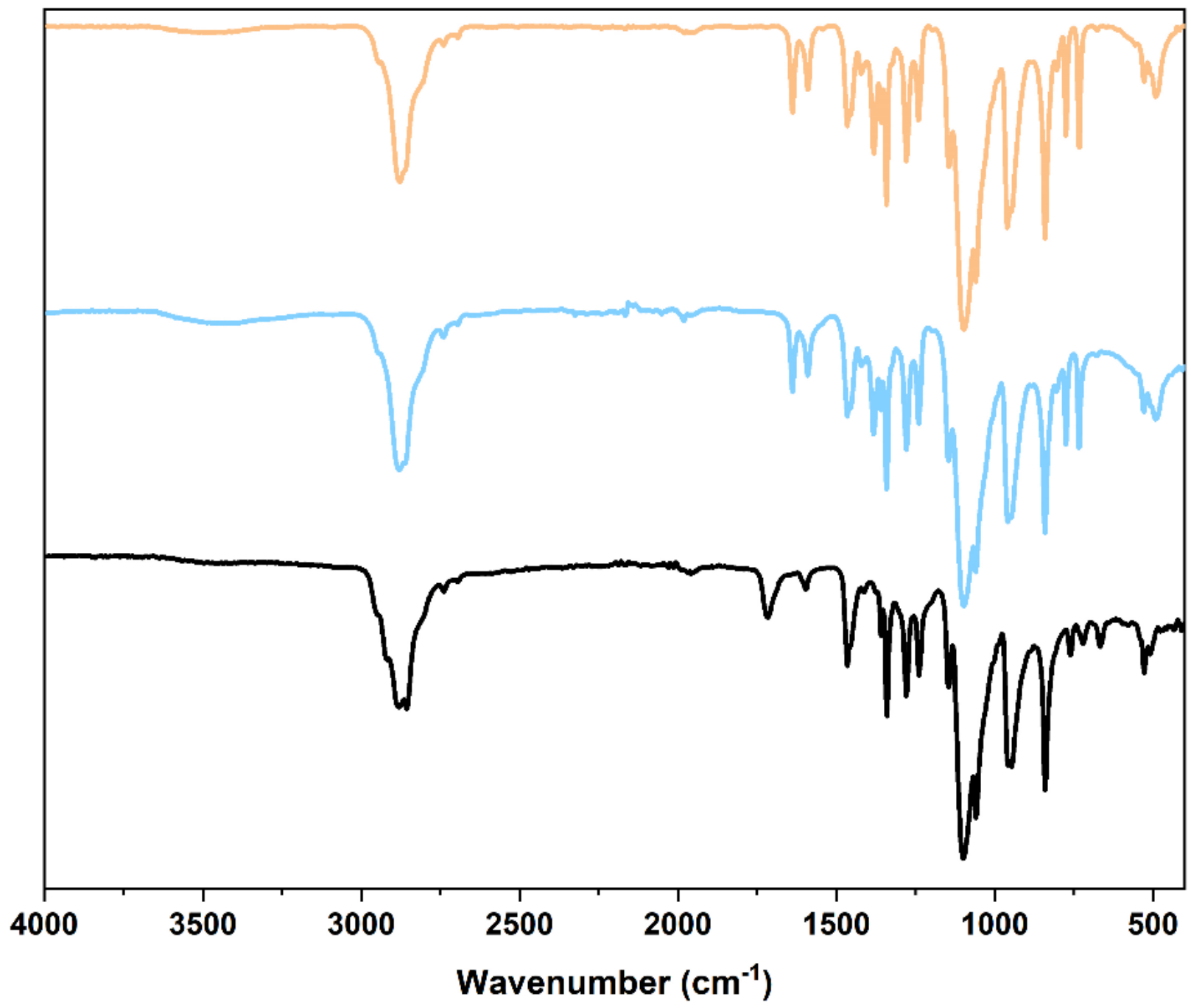


**Figure S31.** FTIR spectra of $H_2L1$ (black, bottom), as-made Cu-BCN-5 (blue, middle) and Cu-BCN-5 after a melting-cooling cycle (orange, top).

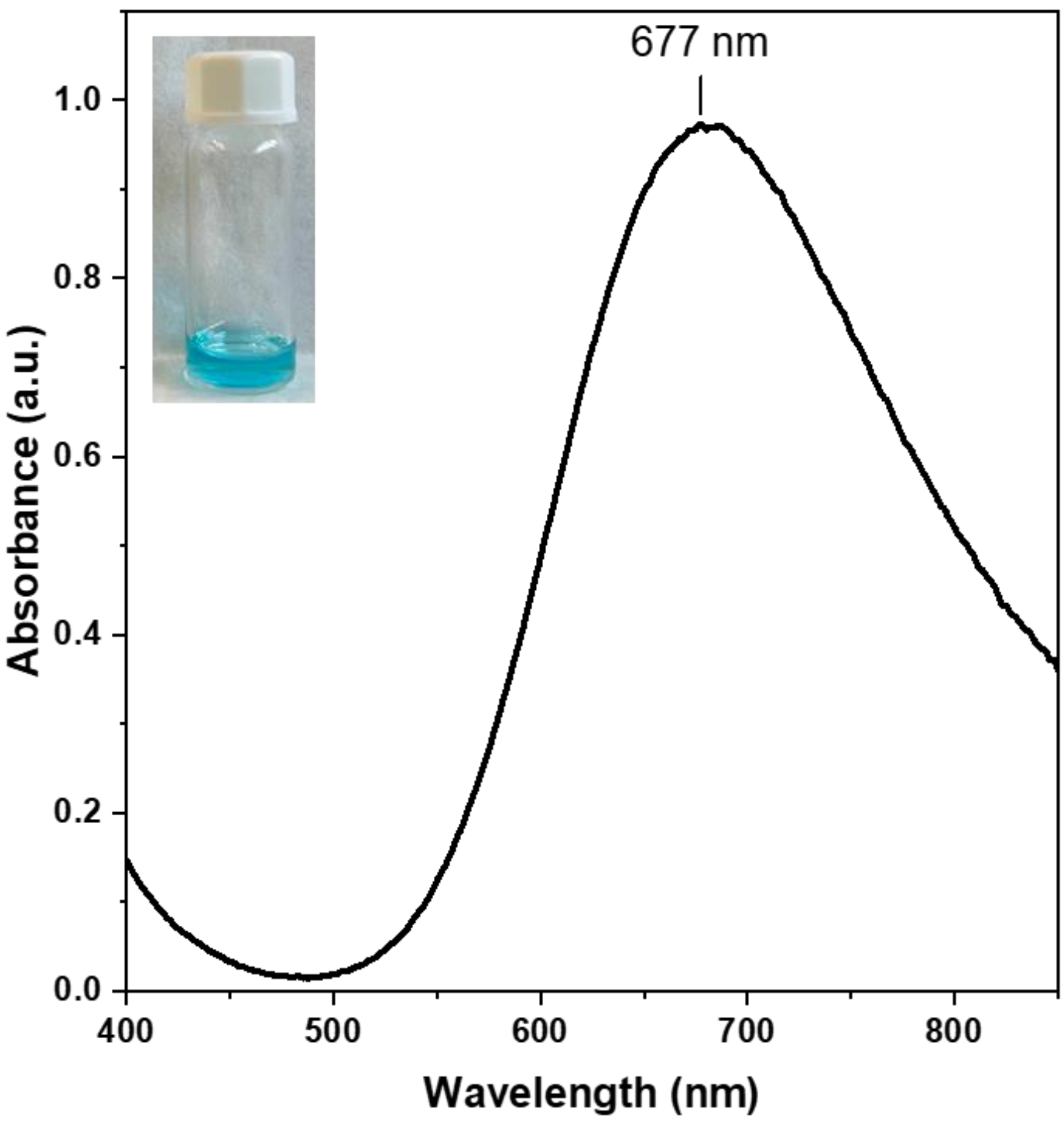


**Figure S32.** UV-Vis spectrum of Cu-BCN-5 after a melting-cooling cycle dissolved in dichloromethane at a concentration of 3.5 mg·mL$^{-1}$.

**S3.5. Mechanical properties**

**S3.5.1. Rh-BCN-5**

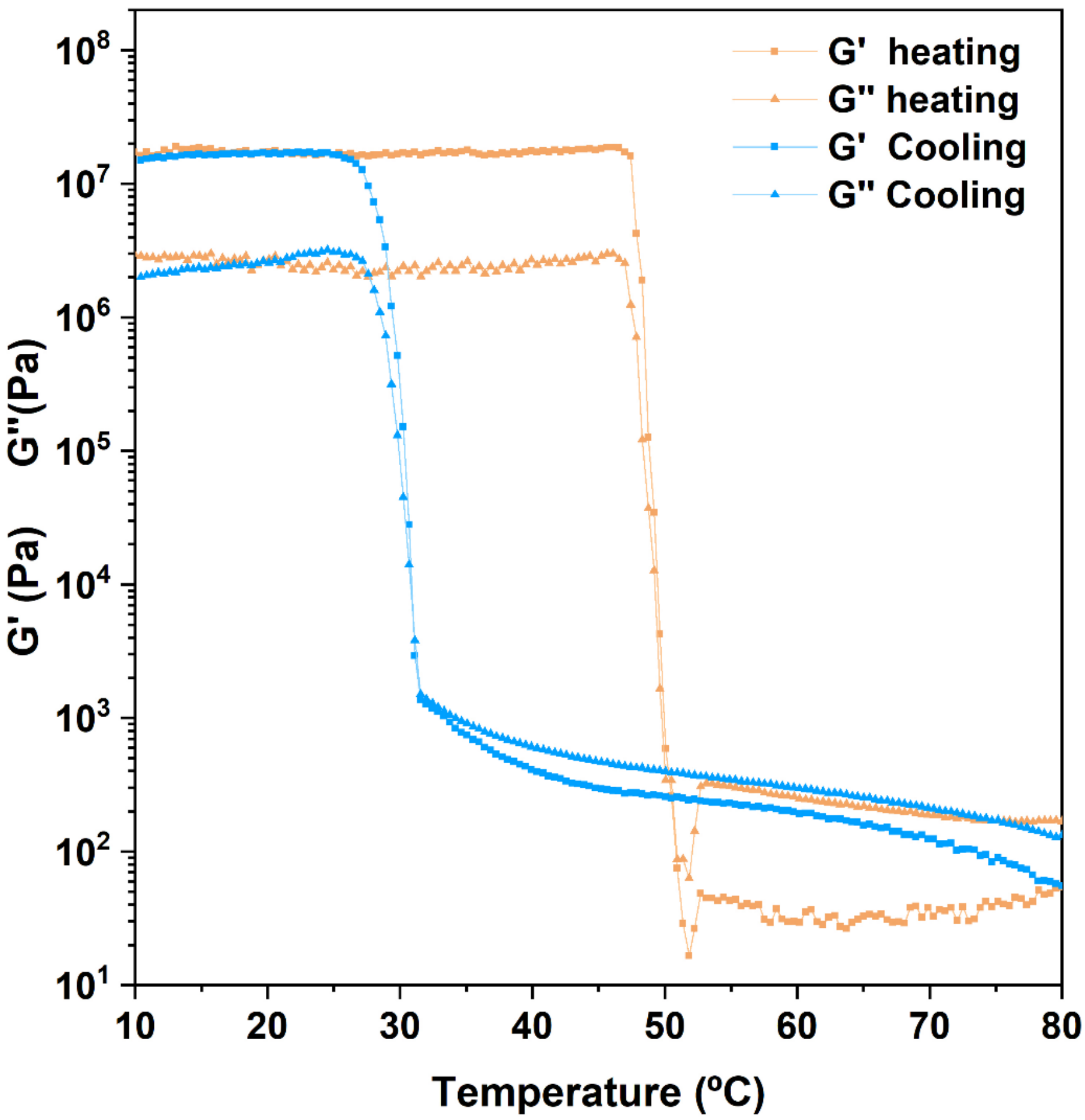


**Figure S33.** Evolution of the storage modulus (G′) and the loss modulus (G″) with temperature for Rh-BCN-5 during a heating and cooling process.

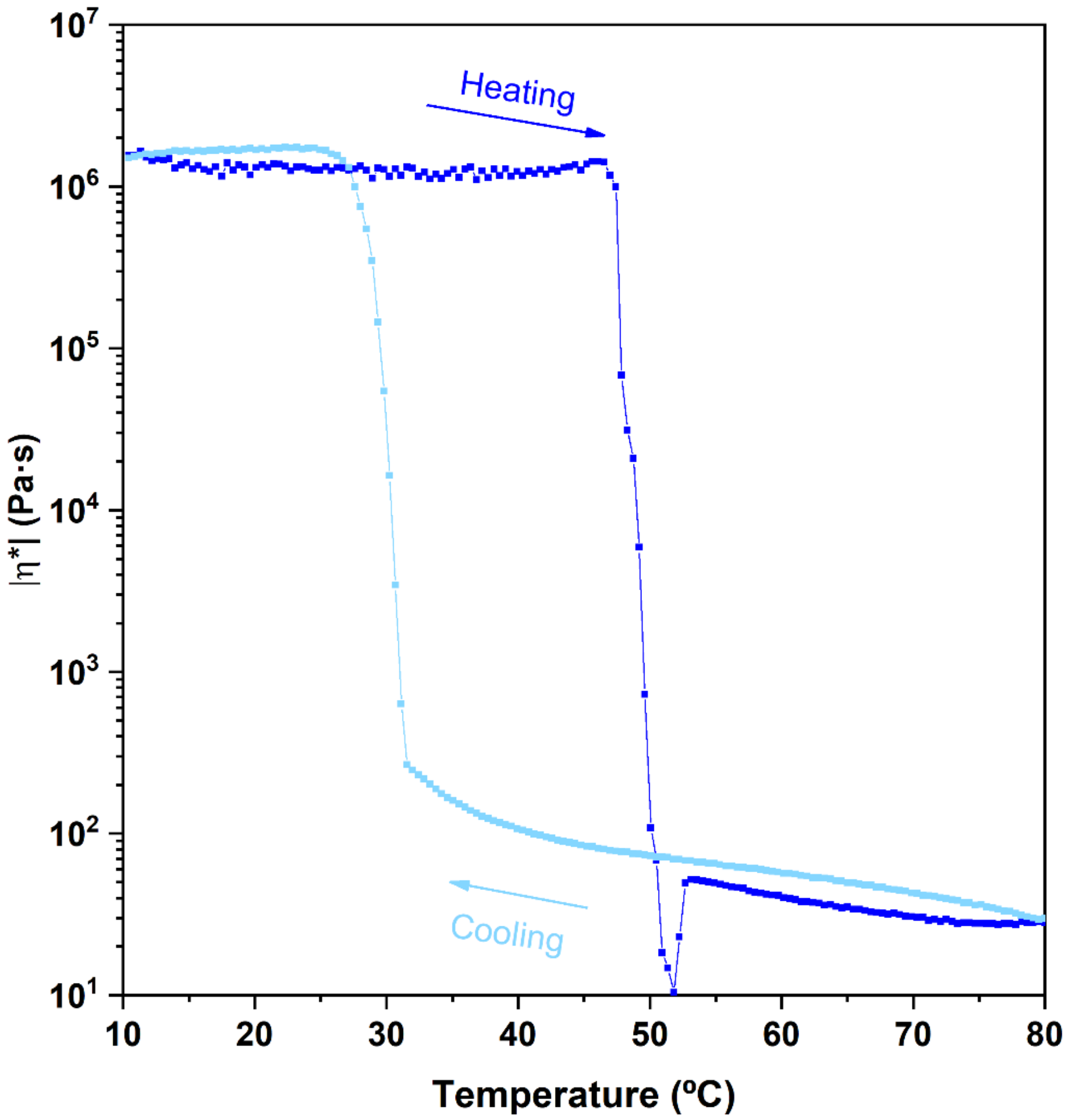


**Figure S34.** Evolution of the complex viscosity (|η*|) with temperature for Rh-BCN-5 during a heating and cooling process.

**S3.5.2. Cu-BCN-5**

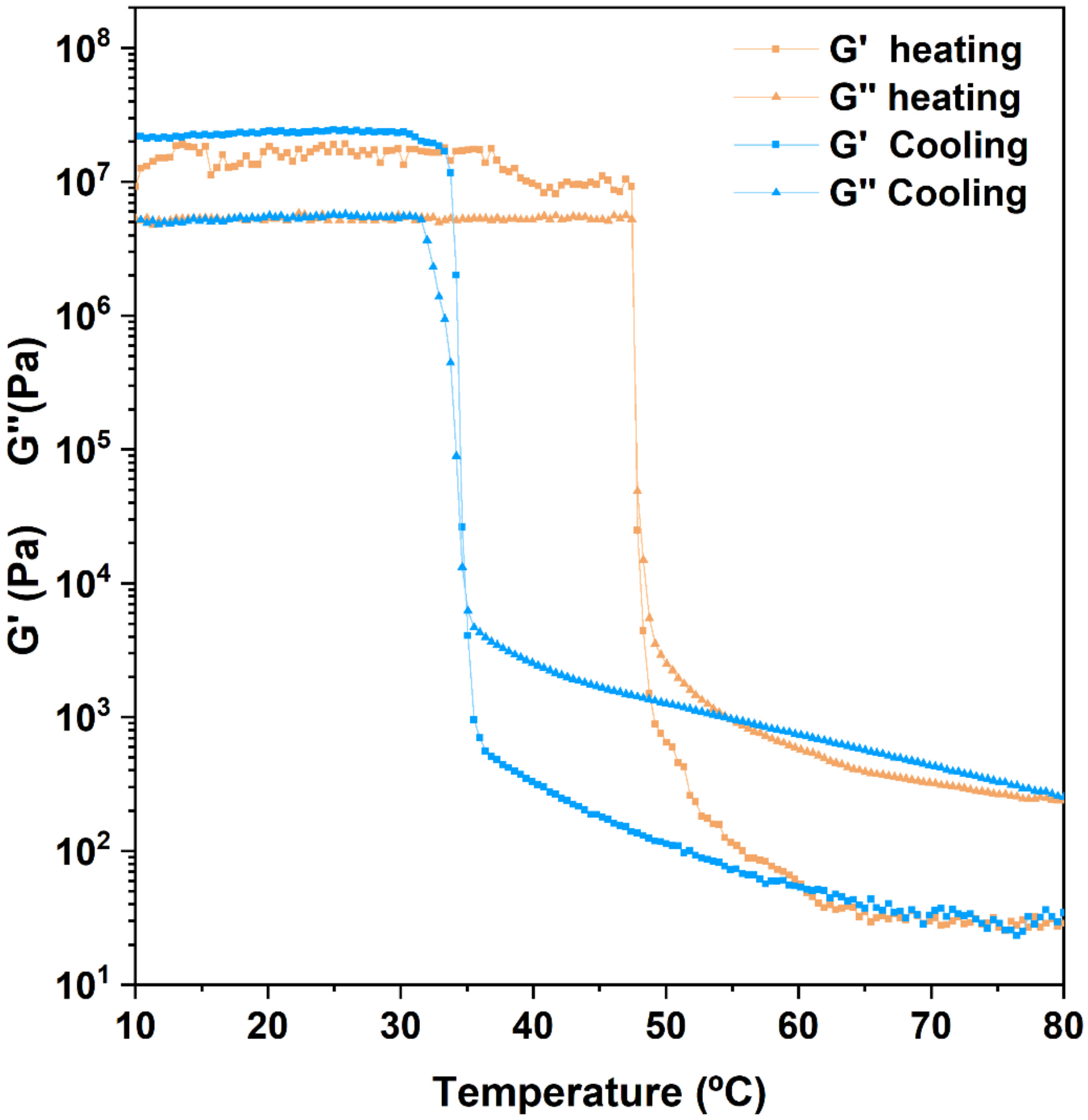


**Figure S35.** Evolution of the storage modulus (G′) and the loss modulus (G″) with temperature for Cu-BCN-5 during a heating and cooling process.

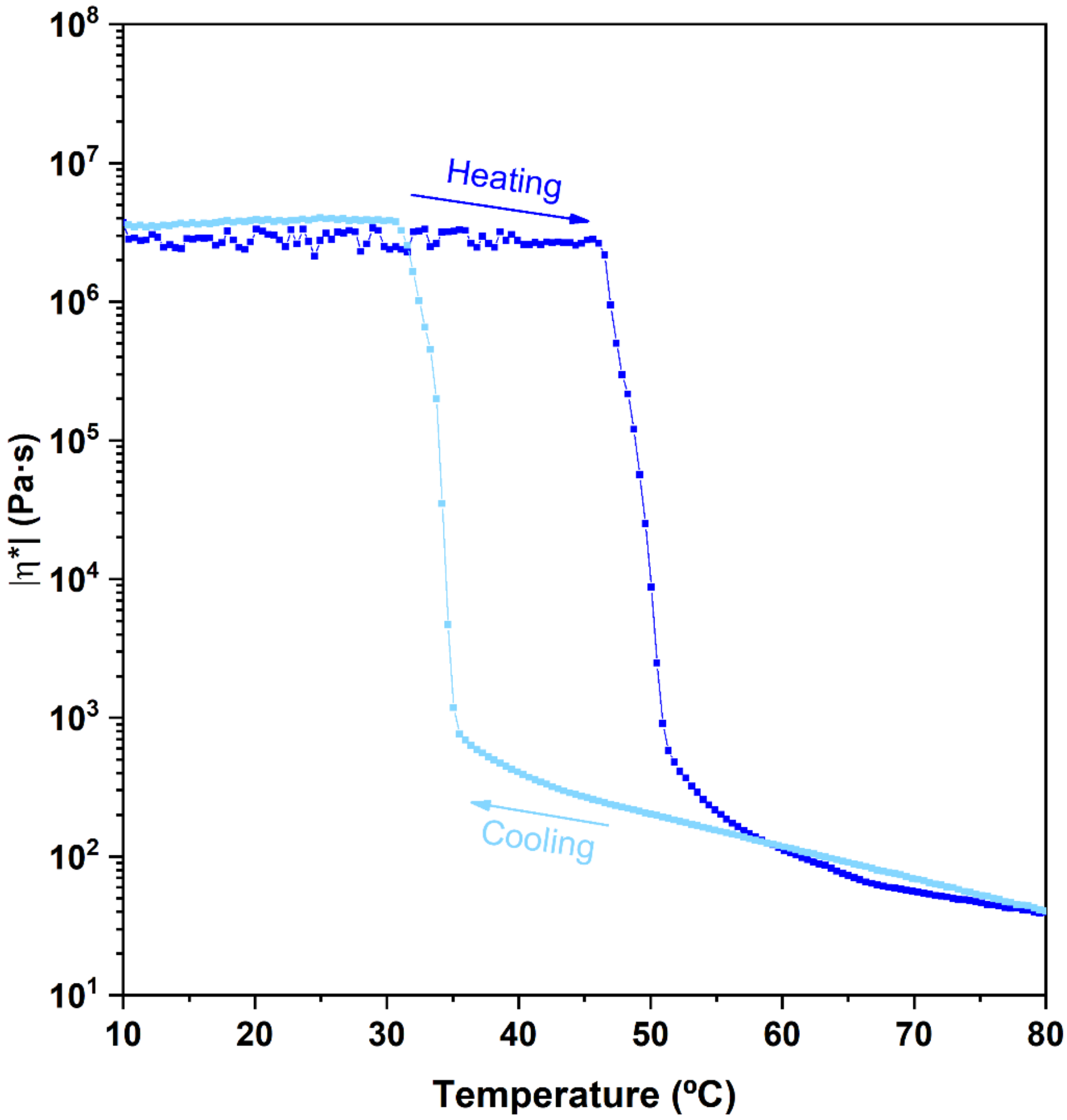


**Figure S36.** Evolution of the complex viscosity (|η*|) with temperature for Cu-BCN-5 during a heating and cooling process.

**S3.6. Porous properties**
**S3.6.1. Rh-BCN-5**

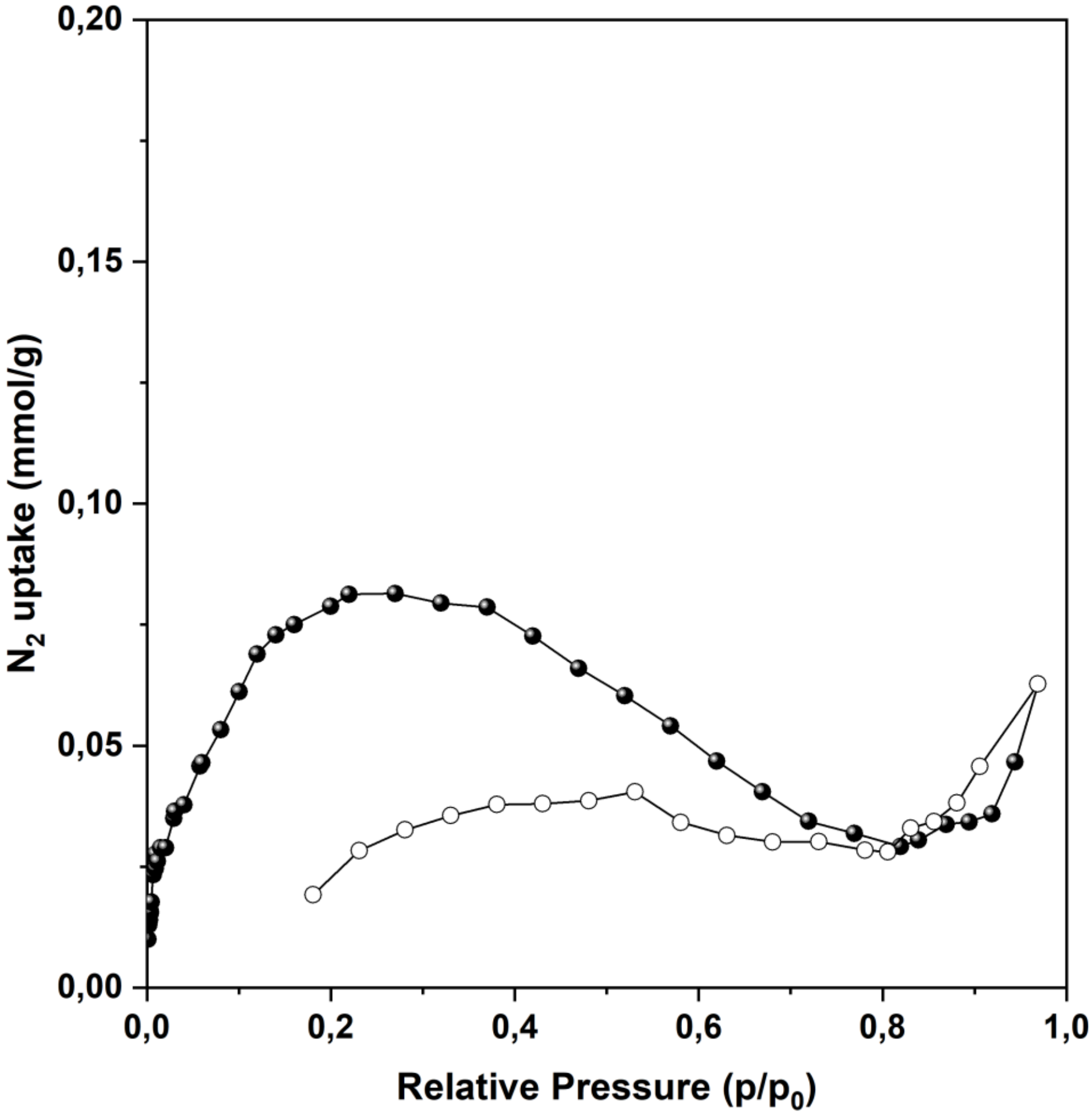


**Figure S37.** $N_2$ adsorption isotherm of Rh-BCN-5 at 77 K.

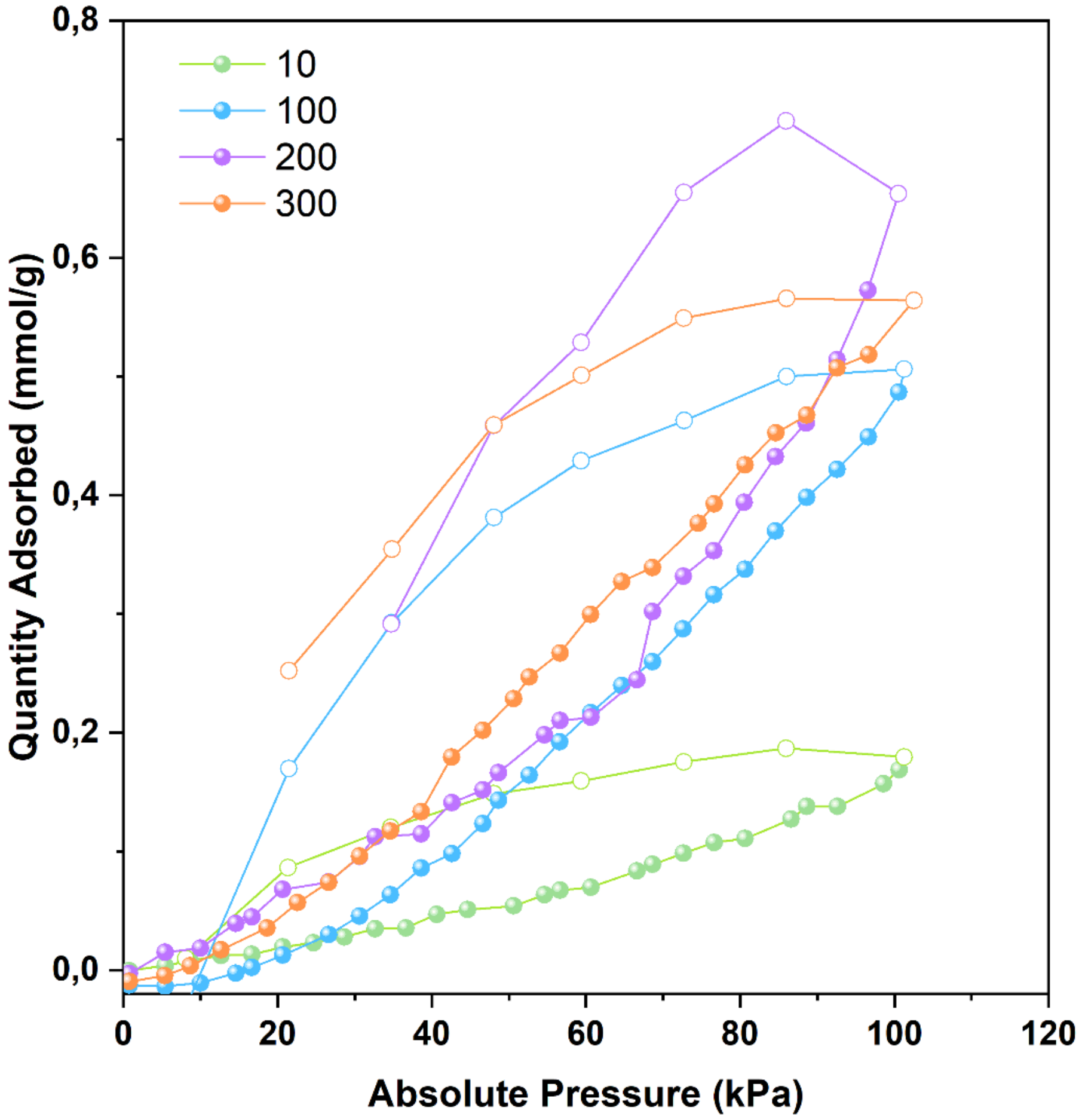


**Figure S38.** $CO_2$ adsorption isotherms of Rh-BCN-5 at 298 K measured at different equilibration times at each pressure step (10 s, 100 s, 200 s and 300 s).

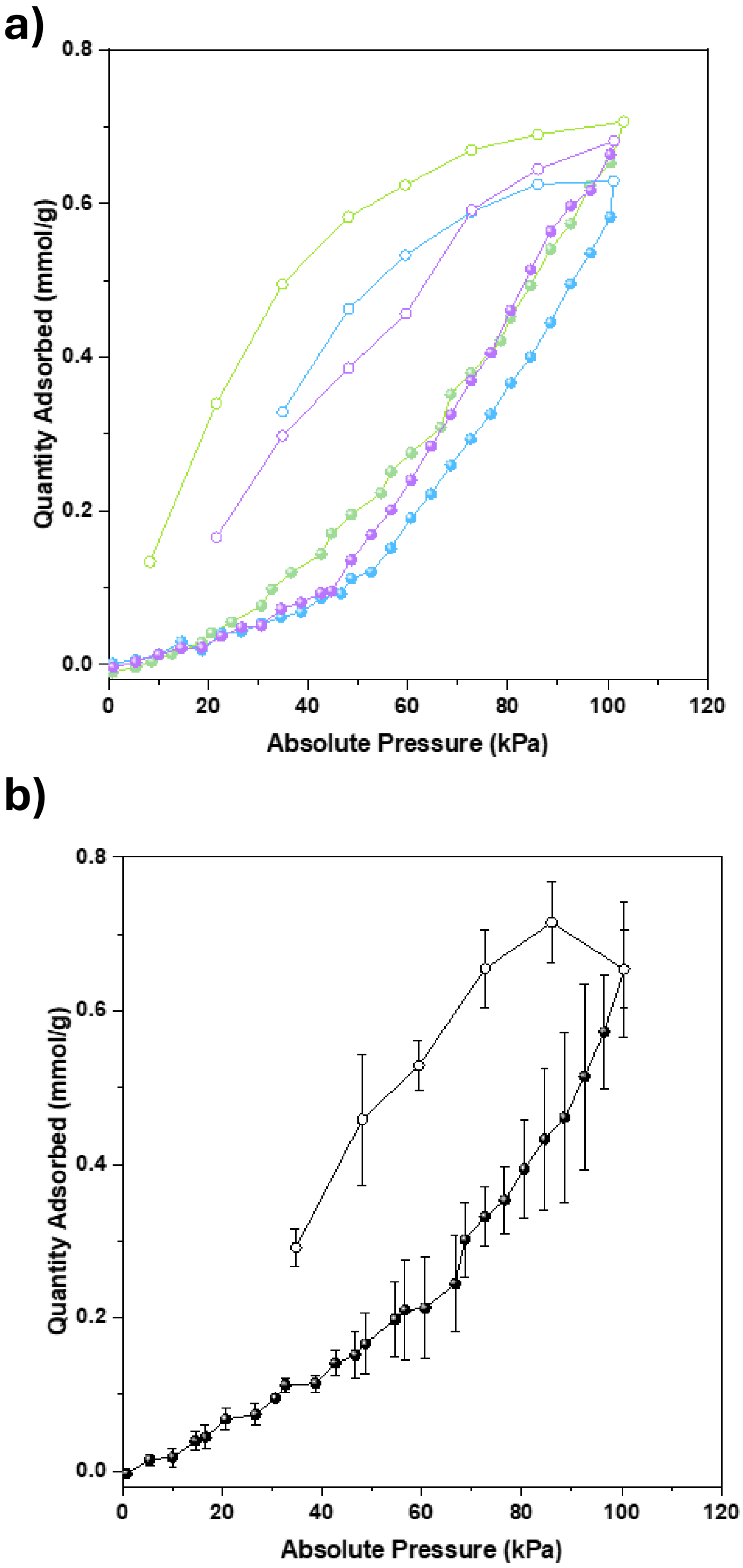


**Figure S39. a)** $CO_2$ adsorption isotherms of three independent Rh-BCN-5 samples measured at 298 K. **b)** $CO_2$ adsorption isotherm showing the average uptake with standard deviation from three independent Rh-BCN-5 samples at 298 K. The equilibration time at each pressure step was 200 s.

a)

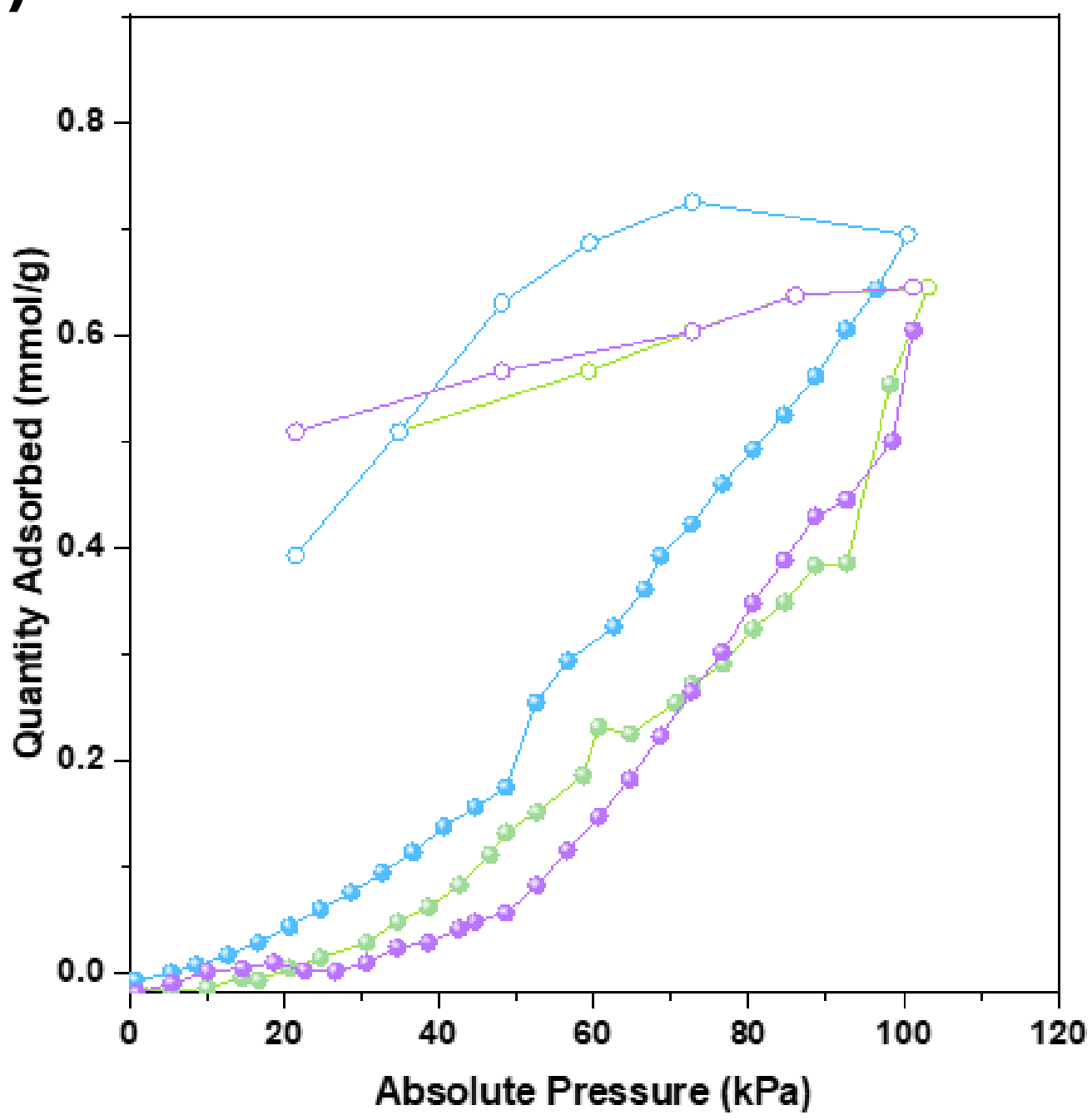


b)

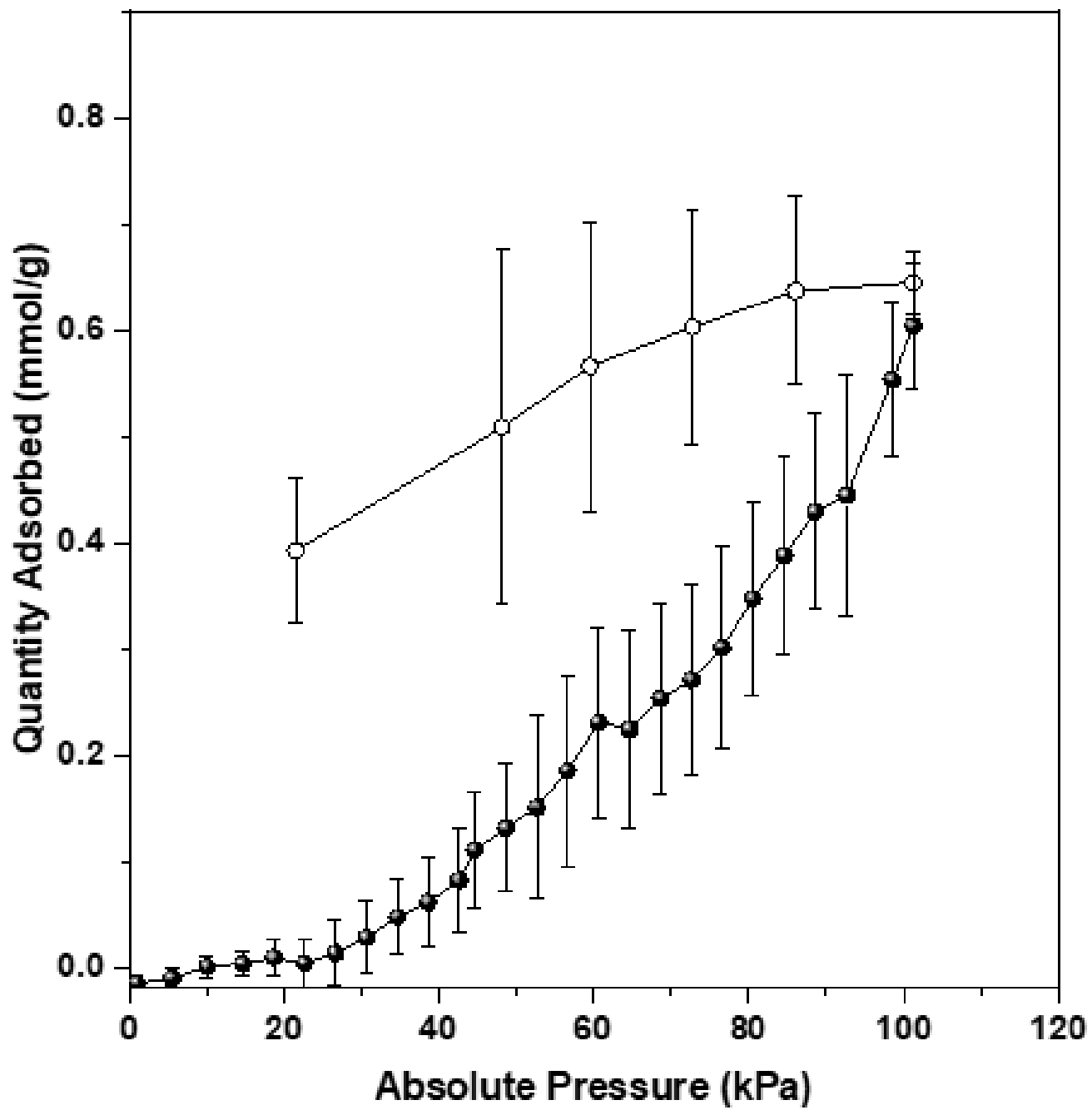


**Figure S40. a)** $CO_2$ adsorption isotherms of three independent Rh-BCN-5 samples after a melting-cooling cycle, measured at 298 K. **b)** $CO_2$ adsorption isotherm showing the average uptake with standard deviation from three independent Rh-BCN-5 samples after a melting-cooling cycle, measured at 298 K. The equilibration time at each pressure step was 200 s.

a)

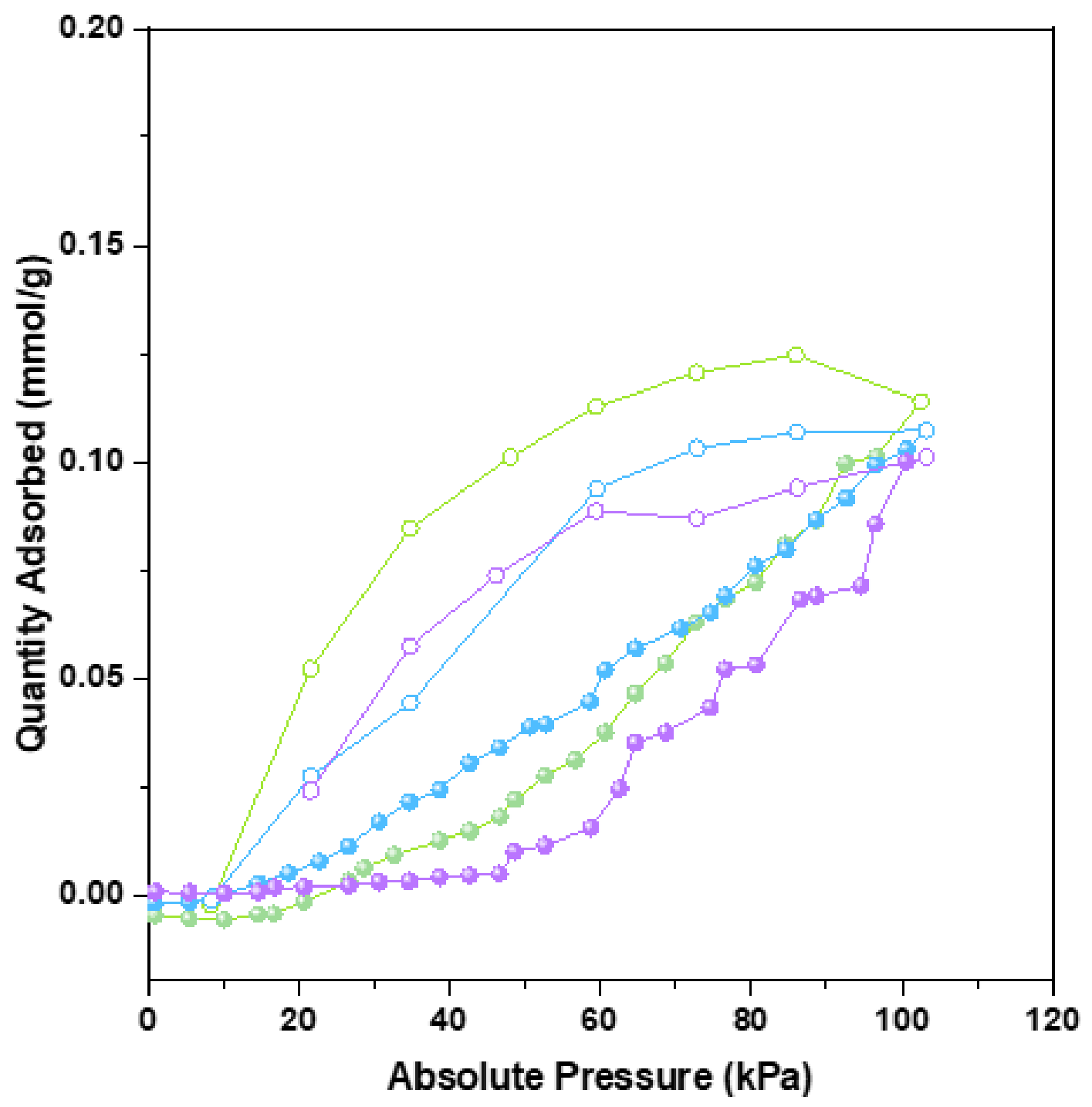


b)

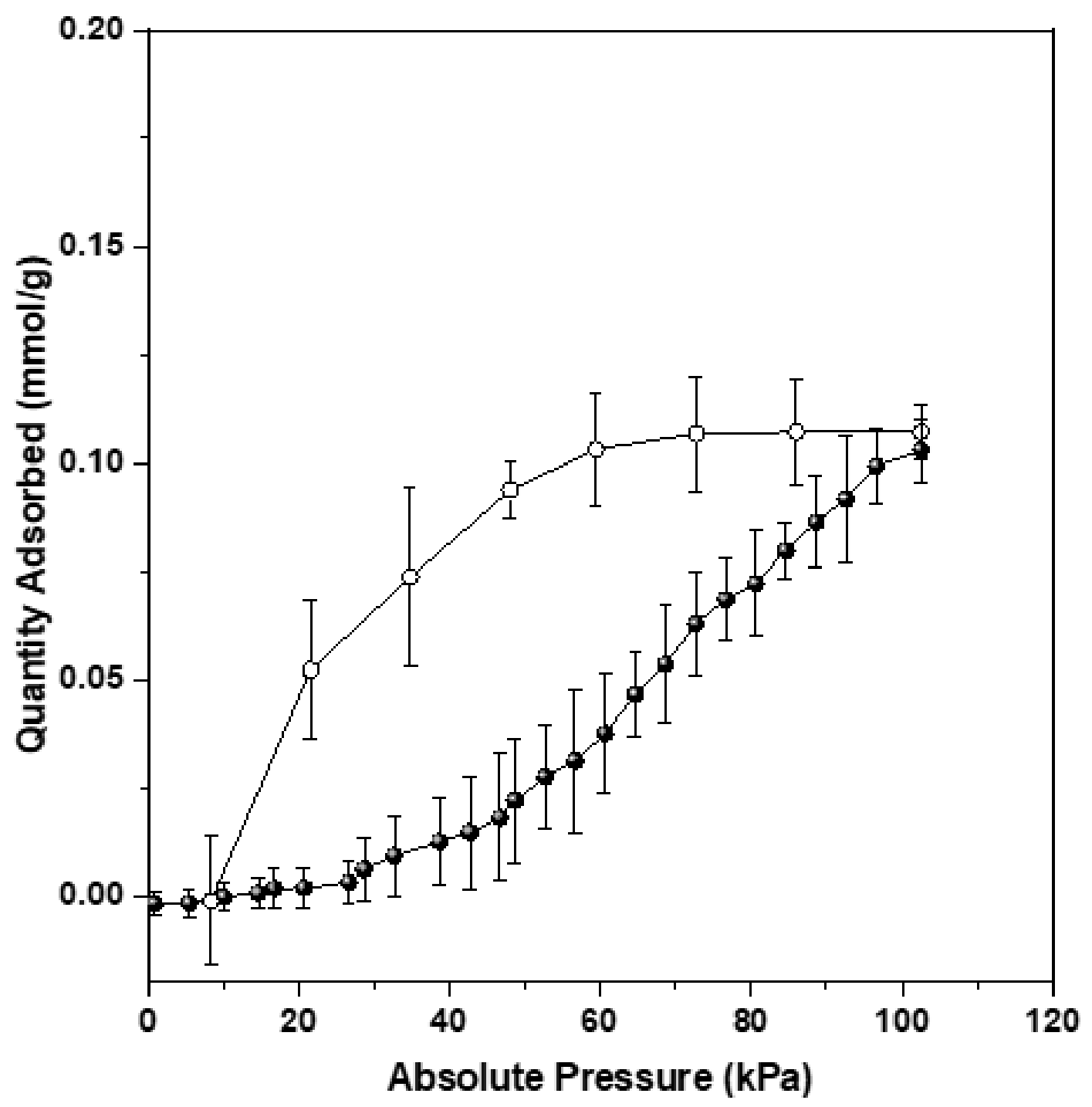


**Figure S41. a)** $CO_2$ adsorption isotherms of three independent liquid Rh-BCN-5 samples at 318 K. **b)** $CO_2$ adsorption isotherm showing the average uptake with standard deviation from three independent liquid samples of Rh-BCN-5 measured at 318 K. The equilibration time at each pressure step was 200 s.

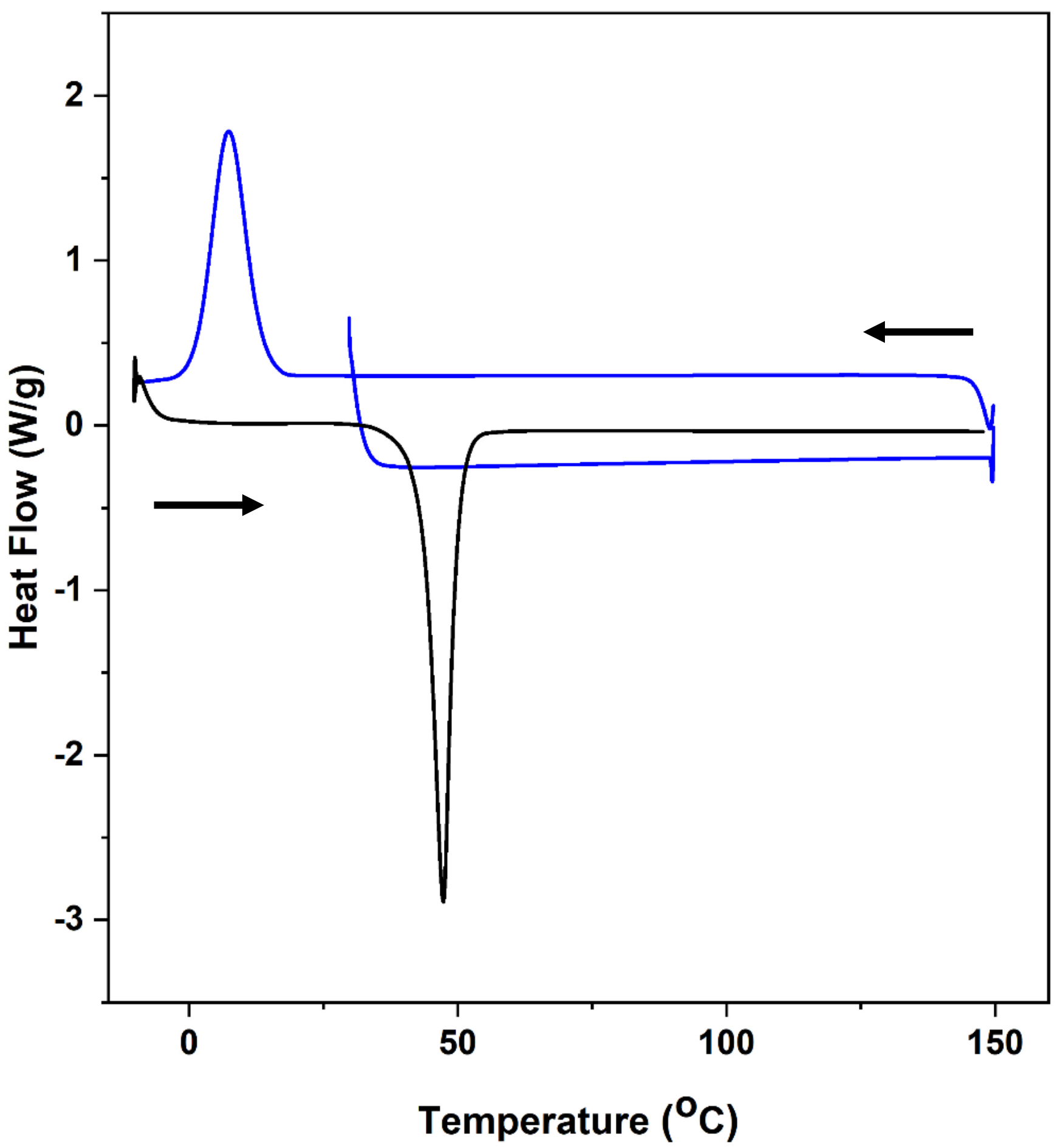


**Figure S42.** DSC curve of Rh-BCN-5 after heating at 65 °C for 15 min and subsequently incubating it at 45 °C overnight. In the first heating run (blue), no melting signal is observed, indicating that the sample remains in the liquid state. A recrystallization signals is observed from cooling, and in the subsequent heating run (black) the melting transition is restored.

**S3.6.2. Cu-BCN-5**

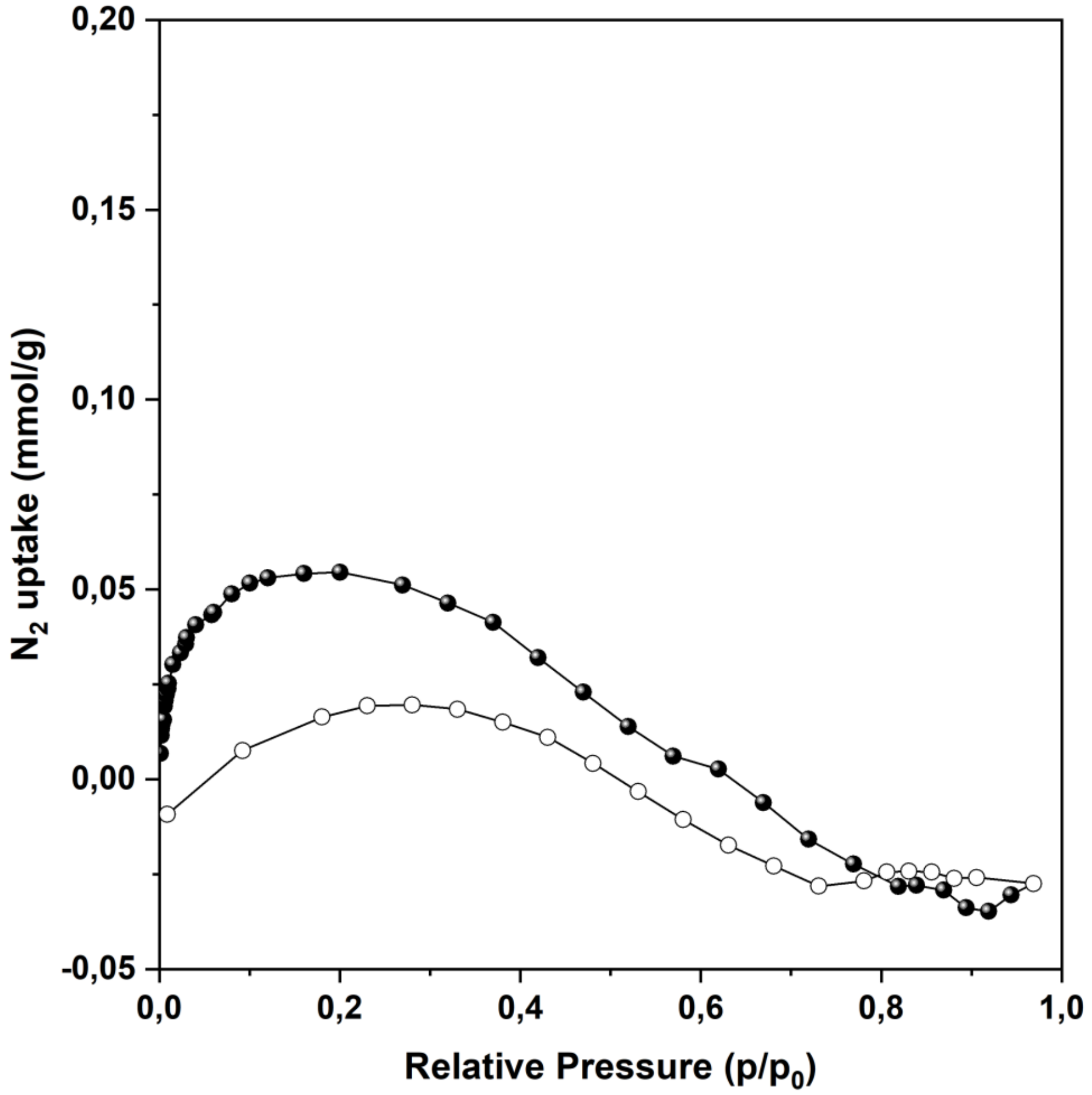


**Figure S43.** $N_2$ adsorption isotherm of Cu-BCN-5 at 77 K.

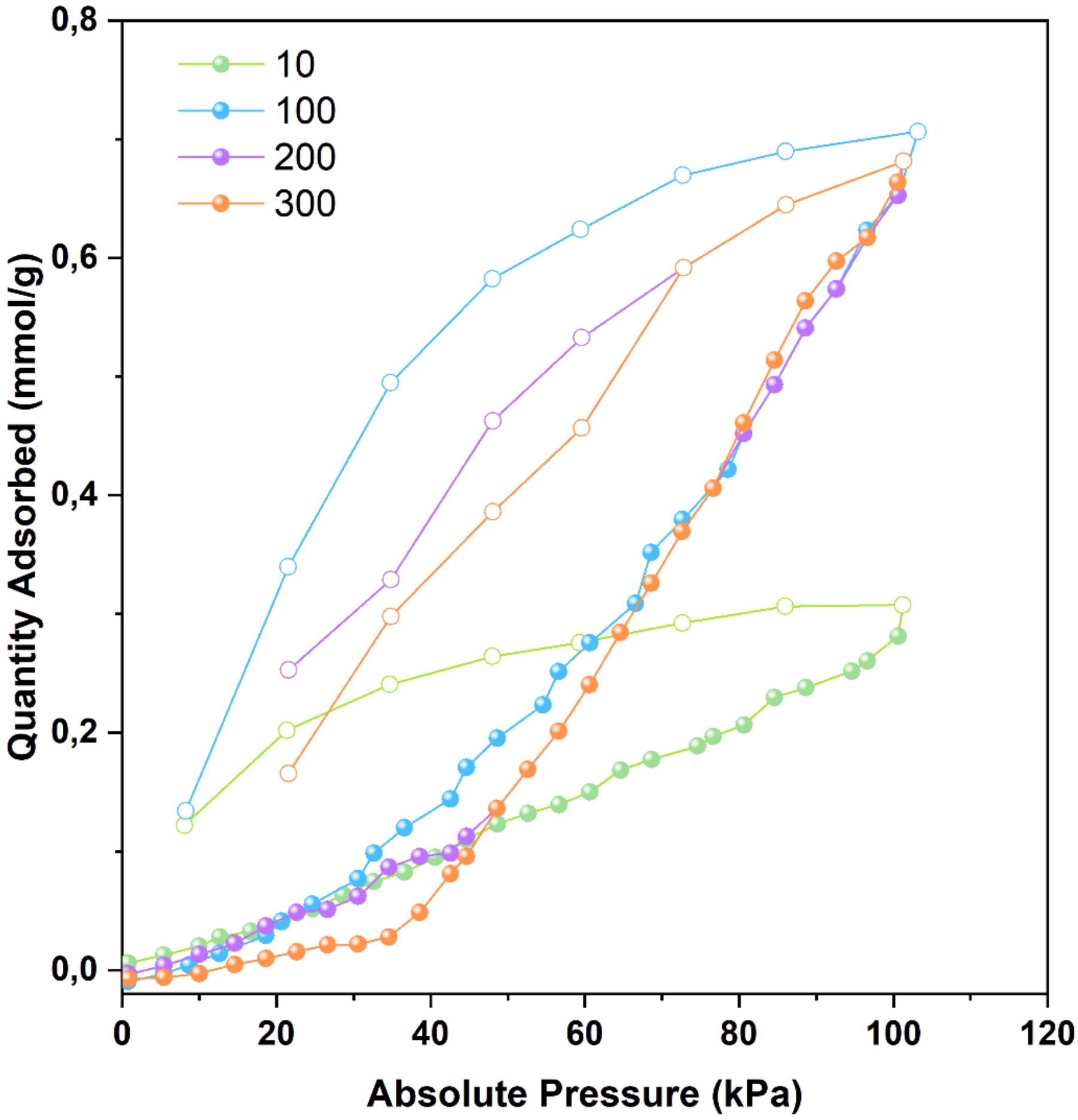


**Figure S44.** $CO_2$ adsorption isotherms of Cu-BCN-5 at 298 K measured at different equilibration times at each pressure step (10 s, 100 s, 200 s and 300 s).

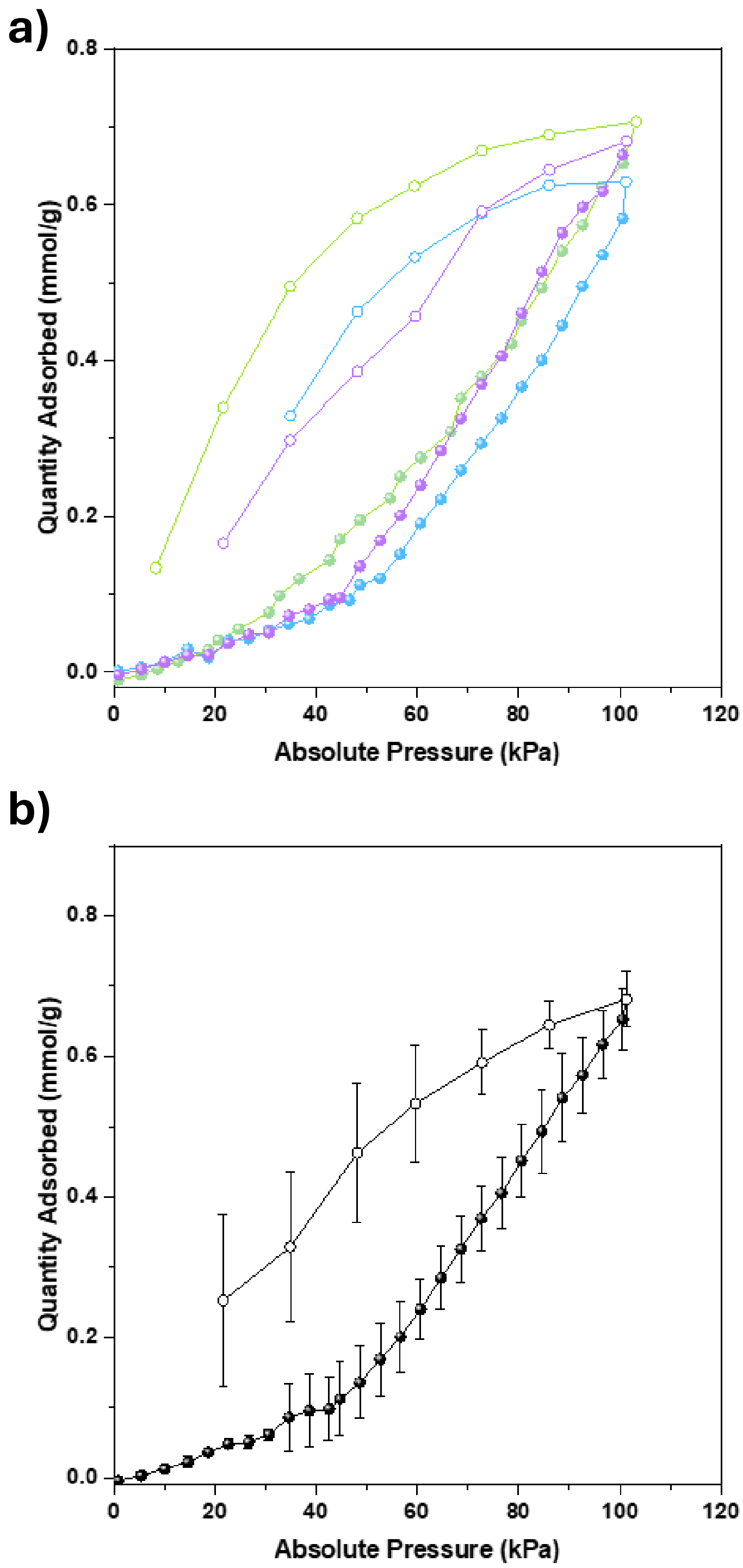


**Figure S45. a)** $CO_2$ adsorption isotherms of three independent Cu-BCN-5 samples measured at 298 K. **b)** $CO_2$ adsorption isotherm showing the average uptake with standard deviation from three independent Cu-BCN-5 samples at 298 K. The equilibration time at each pressure step was 200 s.

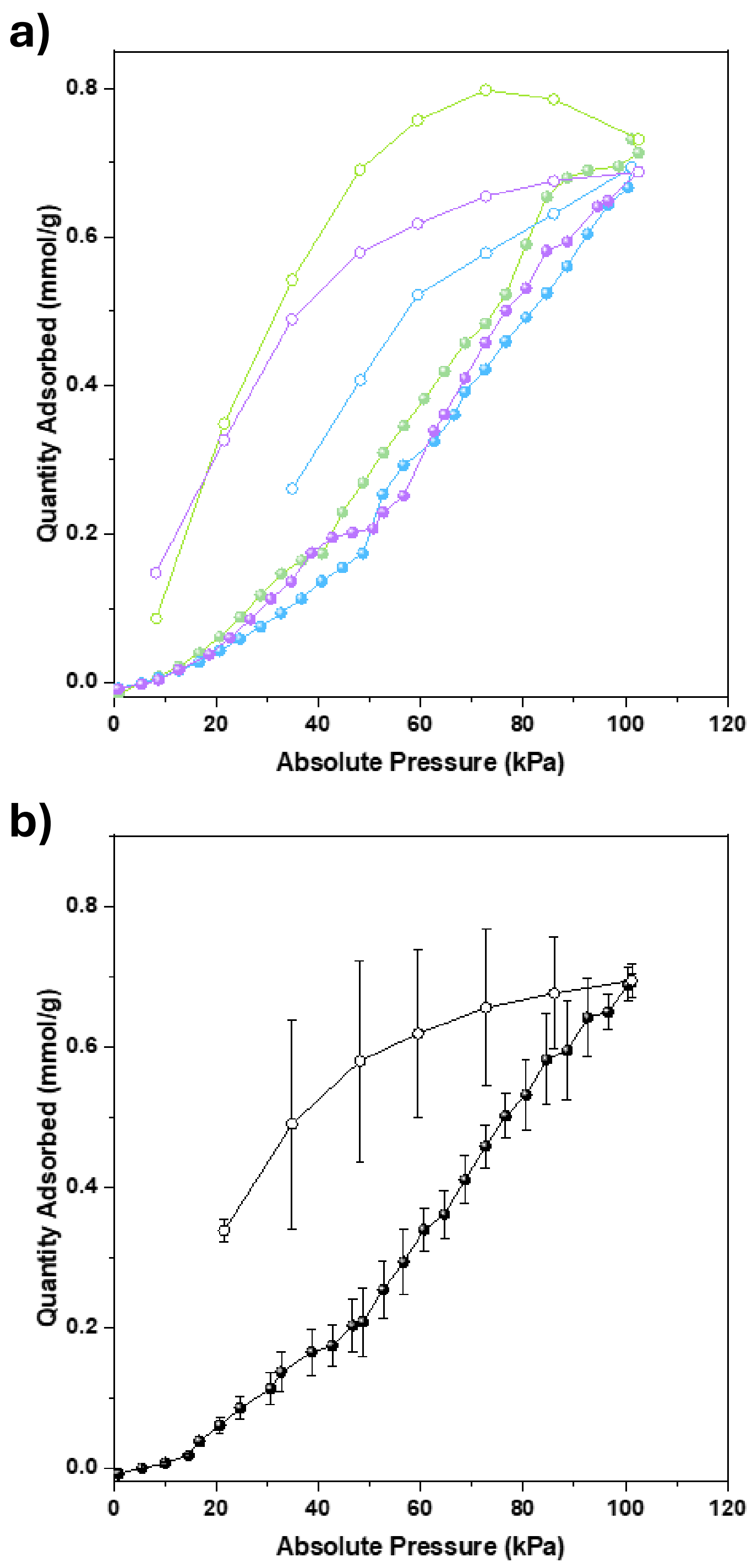


**Figure S46. a)** $CO_2$ adsorption isotherms of three independent Cu-BCN-5 samples after a melting–cooling cycle, measured at 298 K. **b)** $CO_2$ adsorption isotherm showing the average uptake with standard deviation from three independent Cu-BCN-5 samples after a melting-cooling cycle, measured at 298 K. The equilibration time at each pressure step was 200 s.

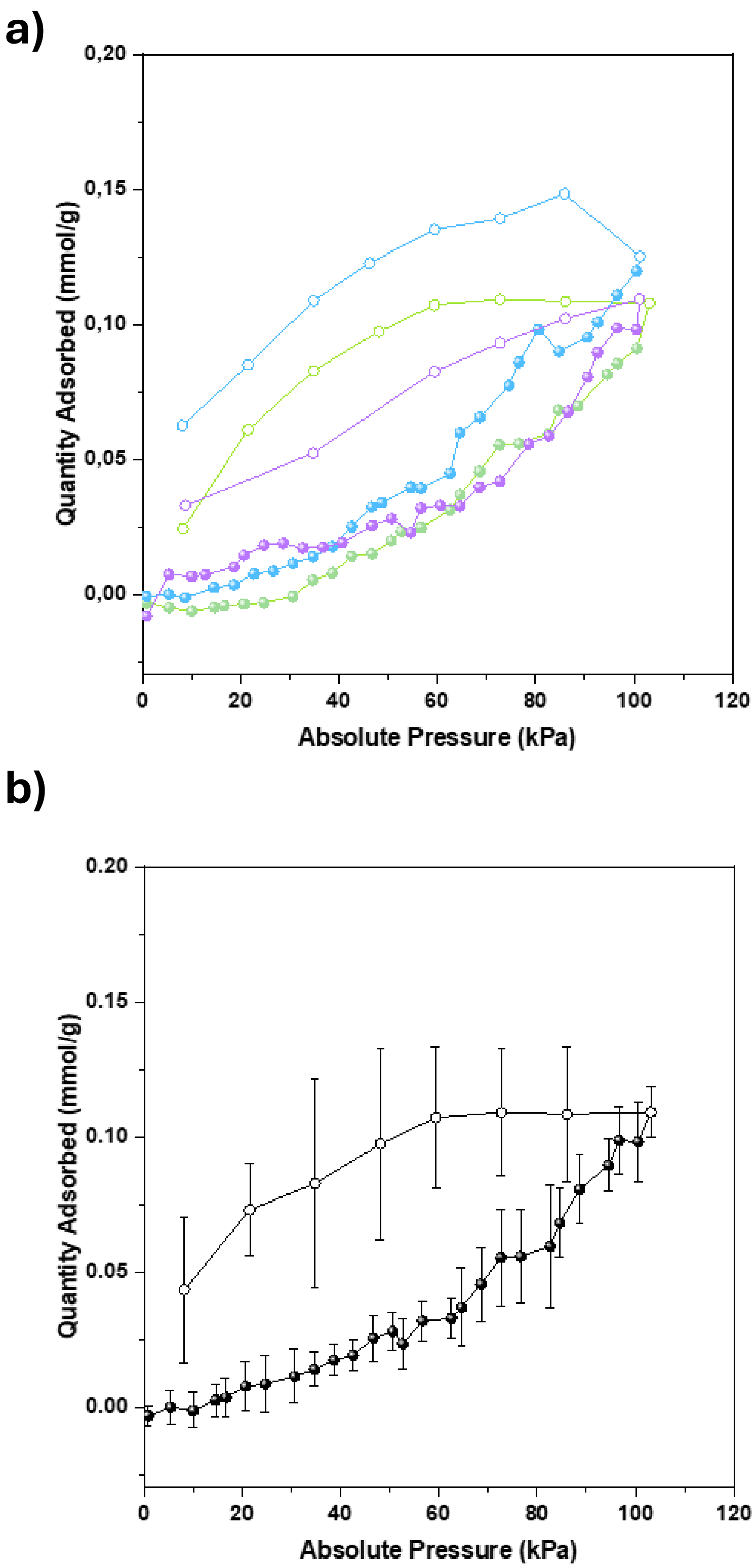


**Figure 47. a)** $CO_2$ adsorption isotherms of three independent liquid Cu-BCN-5 samples at 318 K. **b)** $CO_2$ adsorption isotherm showing the average uptake with standard deviation from three independent liquid samples of Cu-BCN-5 measured at 318 K. The equilibration time at each pressure step was 200 s

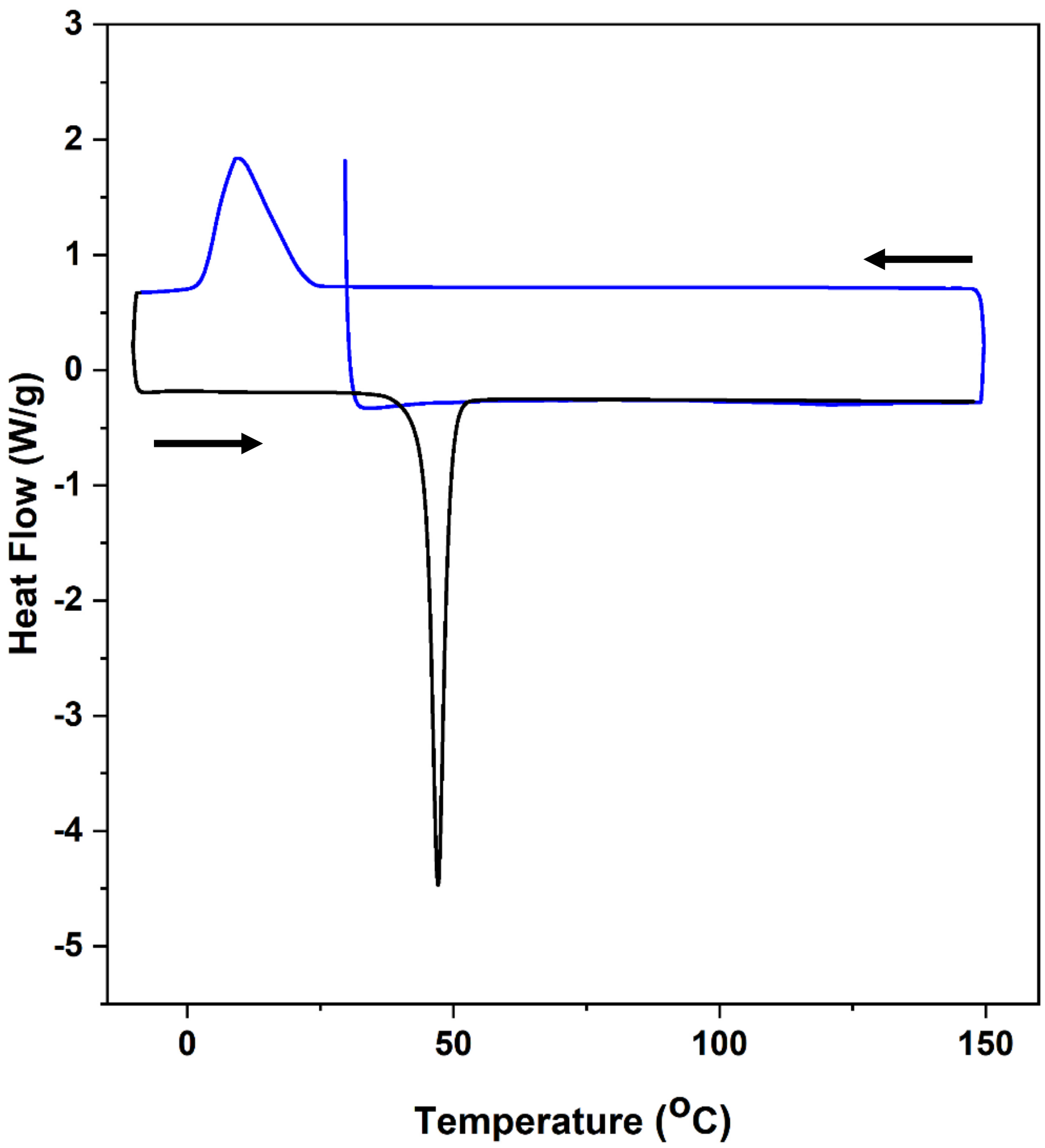


**Figure S48.** DSC curve of Cu-BCN-5 after heating at 65 °C for 15 min and subsequently incubating it at 45 °C overnight. In the first heating run (blue), no melting signal is observed, indicating that the sample remains in the liquid state. A recrystallization signals is observed from cooling, and in the subsequent heating run (black) the melting transition is restored.

**Table S1.** Melting temperature and $CO_2$ uptake at 1 bar of some representative meltable metal-organic materials measured in neat liquid state.

| Material | Type of porous liquid | $CO_2$ uptake at 1 bar ($mmol \cdot g^{-1}$) | Adsorption temperature (K) | Reference |
|---|---|---|---|---|
| PPIL-0.1% | II | 0.14 | 298 | [1] |
| PPL-MSA-5%-Vis | II | 0.44 | 298 | [2] |
| PIL-2-0.5% | II | 0.119 | 298 | [3] |
| PL-2wt% | II | 0,05 | 298 | [4] |
| P5-PL-8% | II | 0.13 | 298 | [5] |
| PL-10wt% | II | 0.125 | 298 | [6] |
| PL2-(SMF-Br) | II | 0.96 | 273 | [7] |
| PIL-diz | II | 0.133 | 273 | [8] |
| CdS/NH2-UiO66 PL | III | 1.3 | 298 | [9] |
| PL-MOF-azo-5% | III | 0,71 | 298 | [10] |
| ZIF-67-PLs-10 | III | 9.542 | 298 | [11] |
| PL1 | III | 1.36 | 273 | [12] |
| $IL1_{0.25}IL2_{0,75}$ | III | 0,75 | 303 | [13] |
| HKUST in silicone oil 25 wt% | III | 0.97 | 298 | [14] |
| MOP-1A | I | 0,044 | 303 | [15] |
| HCS-liquid | I | 0.101 | 298 | [16] |
| UiO-66-2.5%-$NH_2$-20%-PL | I | 0.05 | 298 | [17] |
| OH-MOP-$A_{40}$ | I | 0.032 | 303 | [18] |
| Rh-BCN-5 | I | 0.107 | 318 | This work |
| Cu-BCN-5 | I | 0.109 | 318 | This work |

**Table S2**. Melting temperature and $CO_2$ uptake at 1 bar of some representative meltable metal-organic materials.

| Material | Melting temperature (ºC) | $CO_2$ uptake at 1 bar (mmol·g$^{-1}$) | Adsorption temperature (K) | Reference |
|---|---|---|---|---|
| Rh-BCN-5 | 44 | 0.62 | 298 | This work |
| Cu-BCN-5 | 44 | 0.68 | 298 | This work |
| BCN-93 | 47 | 0.2 | 298 | [19] |
| Im-PL-cage | 48 | 0.25 | 298 | [20] |
| PEGL-Cage | 23 | 0.38 | 275 | [21] |
| MOP-1A | 25 | 0.005 | 273 | [15] |
| 18-C-6-PL | 50 | 0.05 | 298 | [22] |
| $a_g$ RCC3-S | 100-200 | 0.44 | 273 | [23] |
| $a_g$-ZIF-76-mblm | 451 | 0.49 | 298 | [24] |
| $a_g$-ZIF-62 | 432 | 0.75 | 273 | [25] |
| ZIF-62 glass | 434 | 0.49 | 293 | [26] |
| $a_g$-ZIF-UC-7 | 318 | 0.85 | 273 | [27] |
| $a_g$-TIF-4(33%) | 460 | 0.509 | 298 | [28] |
| $a_g$-AFI-[Zn(Im)$_{1.68}$(bIm)$_{0.32}$] | 432 | 1.12 | 273 | [29] |
| $a_g$-ZIF-4 | 579 | 1 | 273 | [30] |
| $a_g$-ZIF-zni | 585 | 1.21 | 273 | [30] |

**S3.6.3. Blank samples**

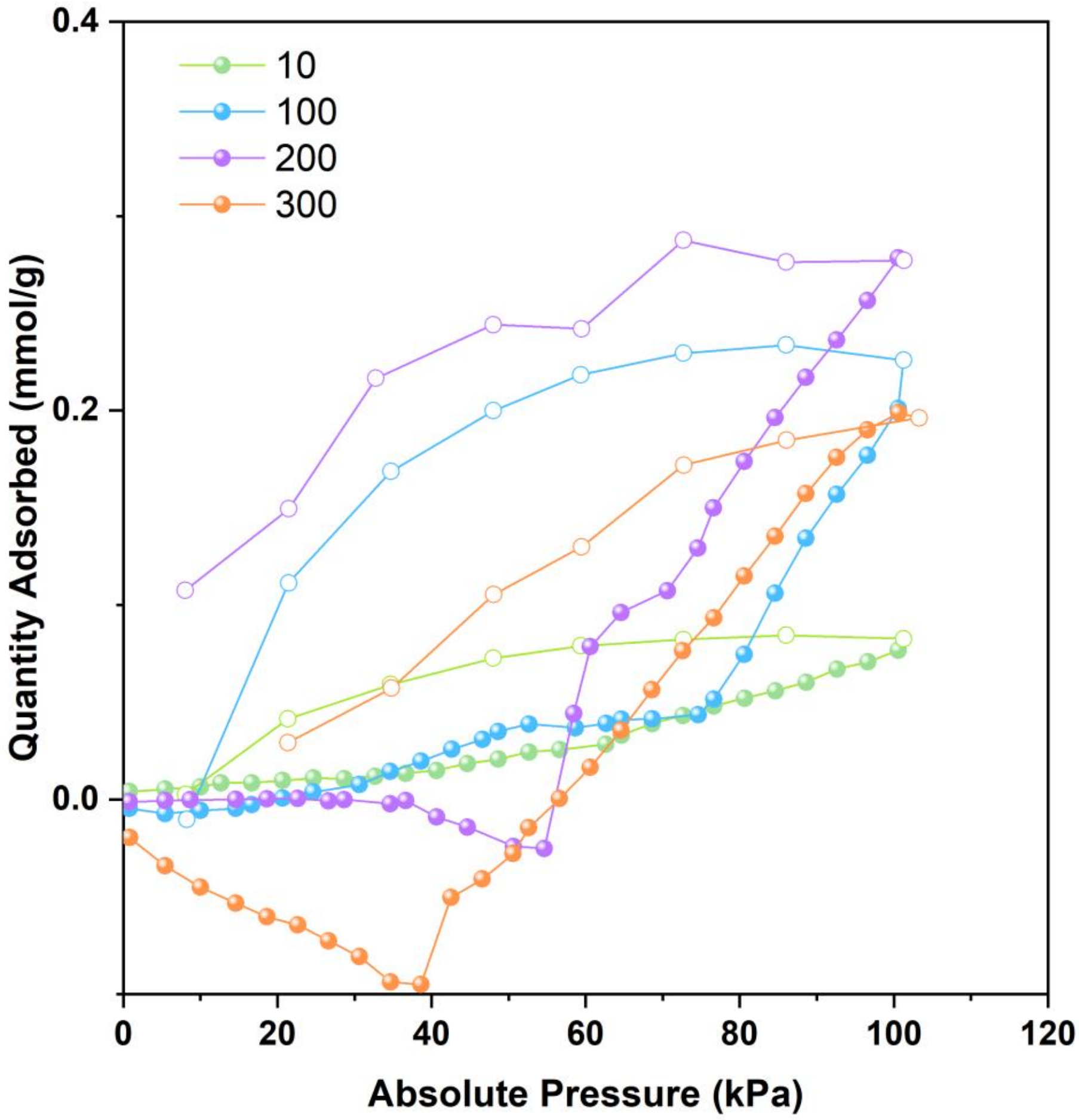


**Figure S49.** $CO_2$ adsorption isotherms of $Me_2L1$ at 298 K measured at different equilibration times per pressure step (10 s, 100 s, 200 s and 300 s).

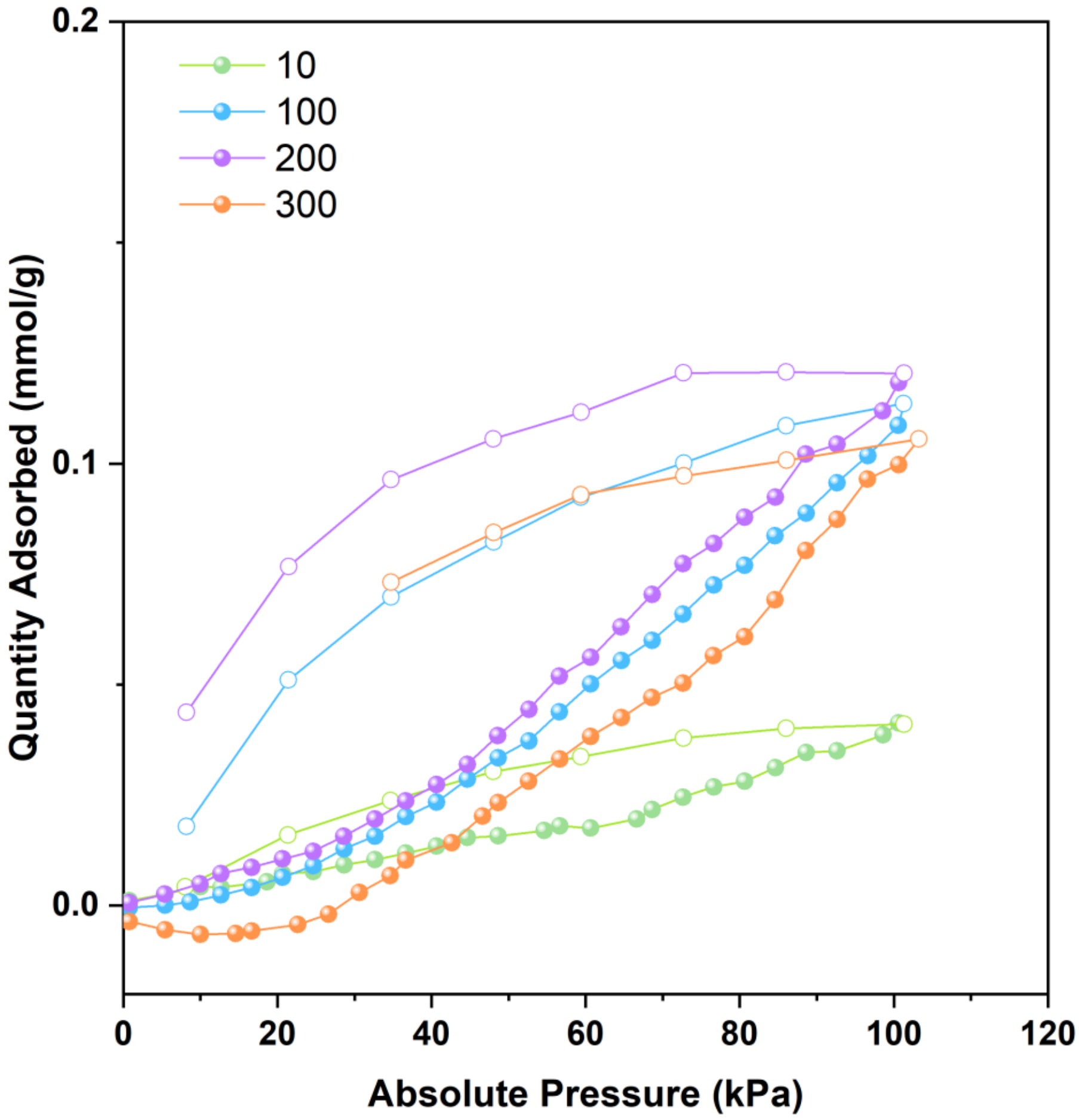


**Figure S50.** $CO_2$ adsorption isotherms of the physical mixture containing semicrystalline $Me_2L1$ and rhodium(II) acetate at 298 K measured at different equilibration times per pressure step (10 s, 100 s, 200 s and 300 s).

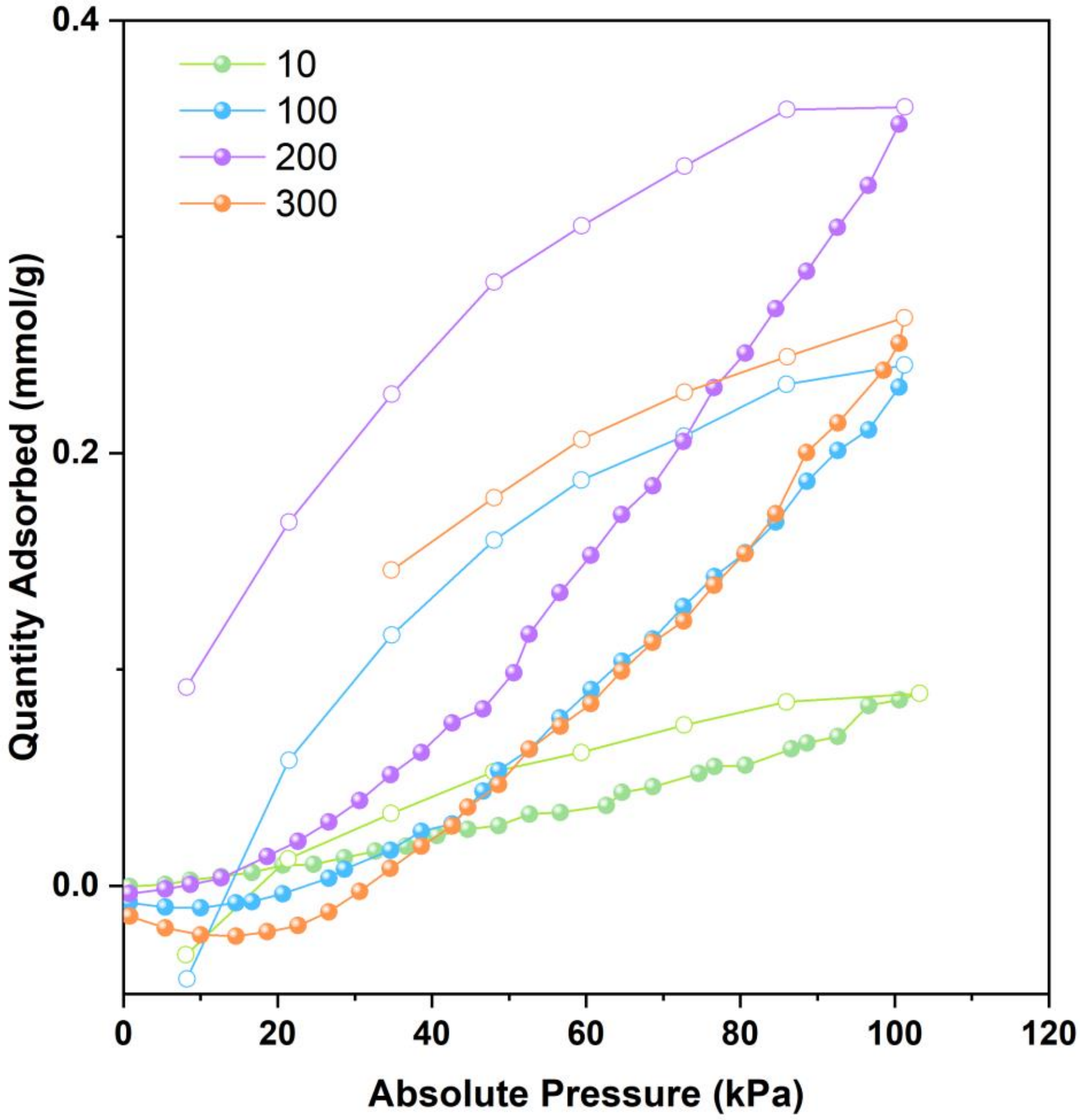


**Figure S51.** $CO_2$ adsorption isotherms of the physical mixture containing semicrystalline $Me_2L1$ and copper(II) acetate at 298 K measured at different equilibration times per pressure step (10 s, 100 s, 200 s and 300 s).

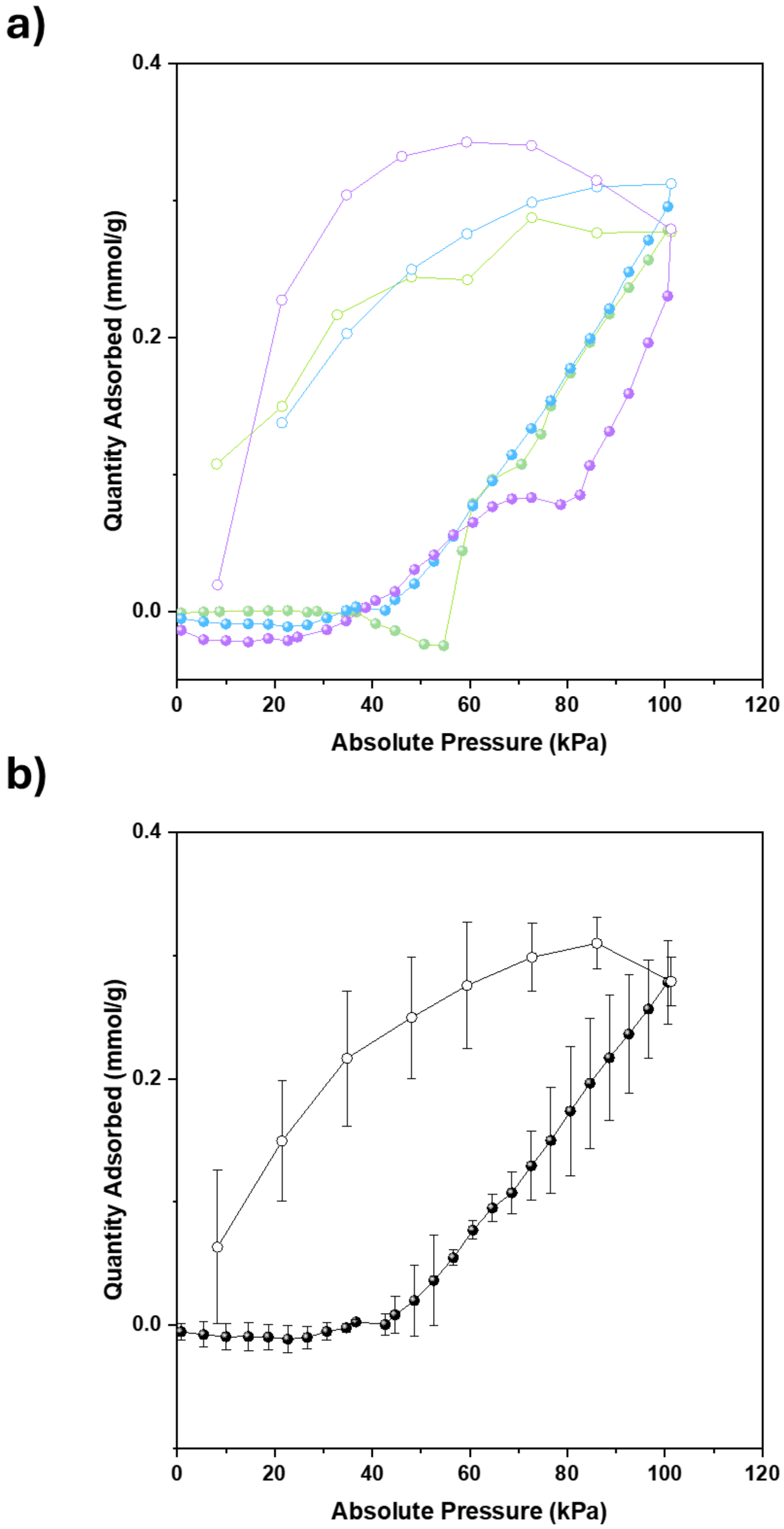


**Figure S52. a)** $CO_2$ adsorption isotherms of three independent $Me_2L1$ samples measured at 298 K. **b)** $CO_2$ adsorption isotherm showing the average uptake with standard deviation from three independent $Me_2L1$ samples at 298 K. The equilibration time at each pressure step was 200 s.

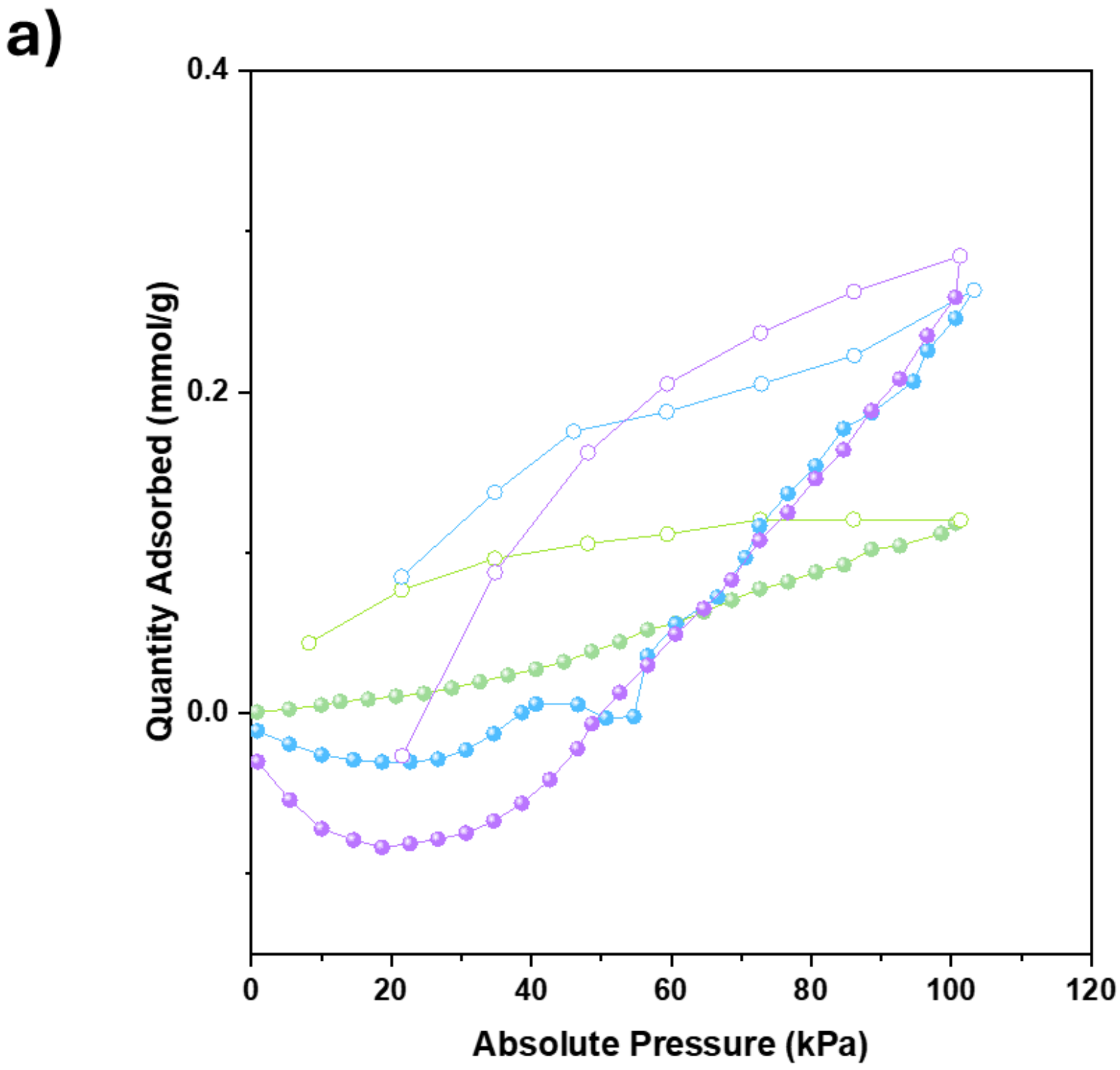


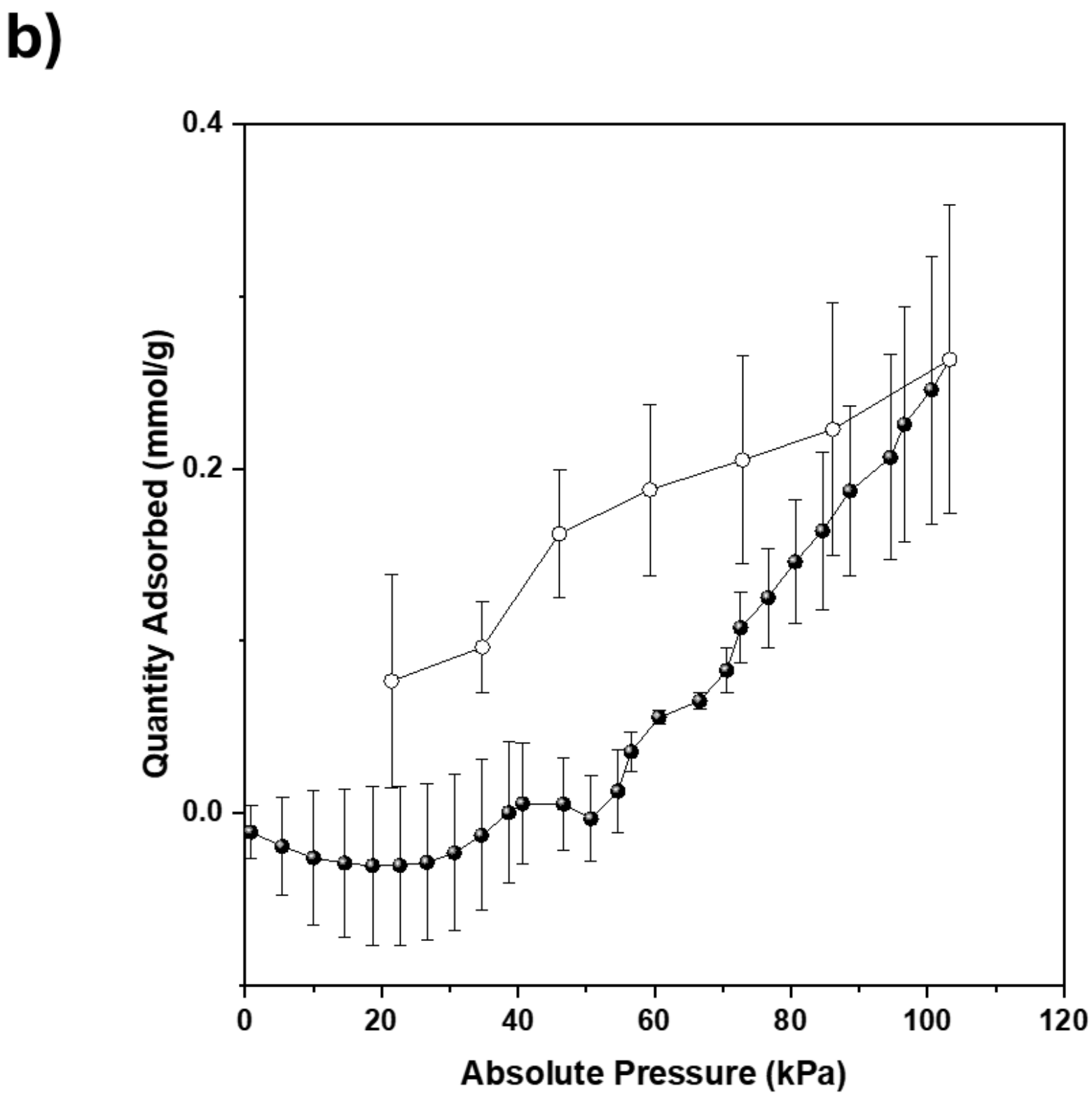


**Figure S53. a)** $CO_2$ adsorption isotherms of three independent samples composed of the physical mixture containing semicrystalline $Me_2L1$ and rhodium(II) acetate measured at 298 K. **b)** $CO_2$ adsorption isotherm showing the average uptake with standard deviation from three independent samples composed of the physical mixture containing semicrystalline $Me_2L1$ and rhodium(II) acetate at 298 K. The equilibration time at each pressure step was 200 s.

a)

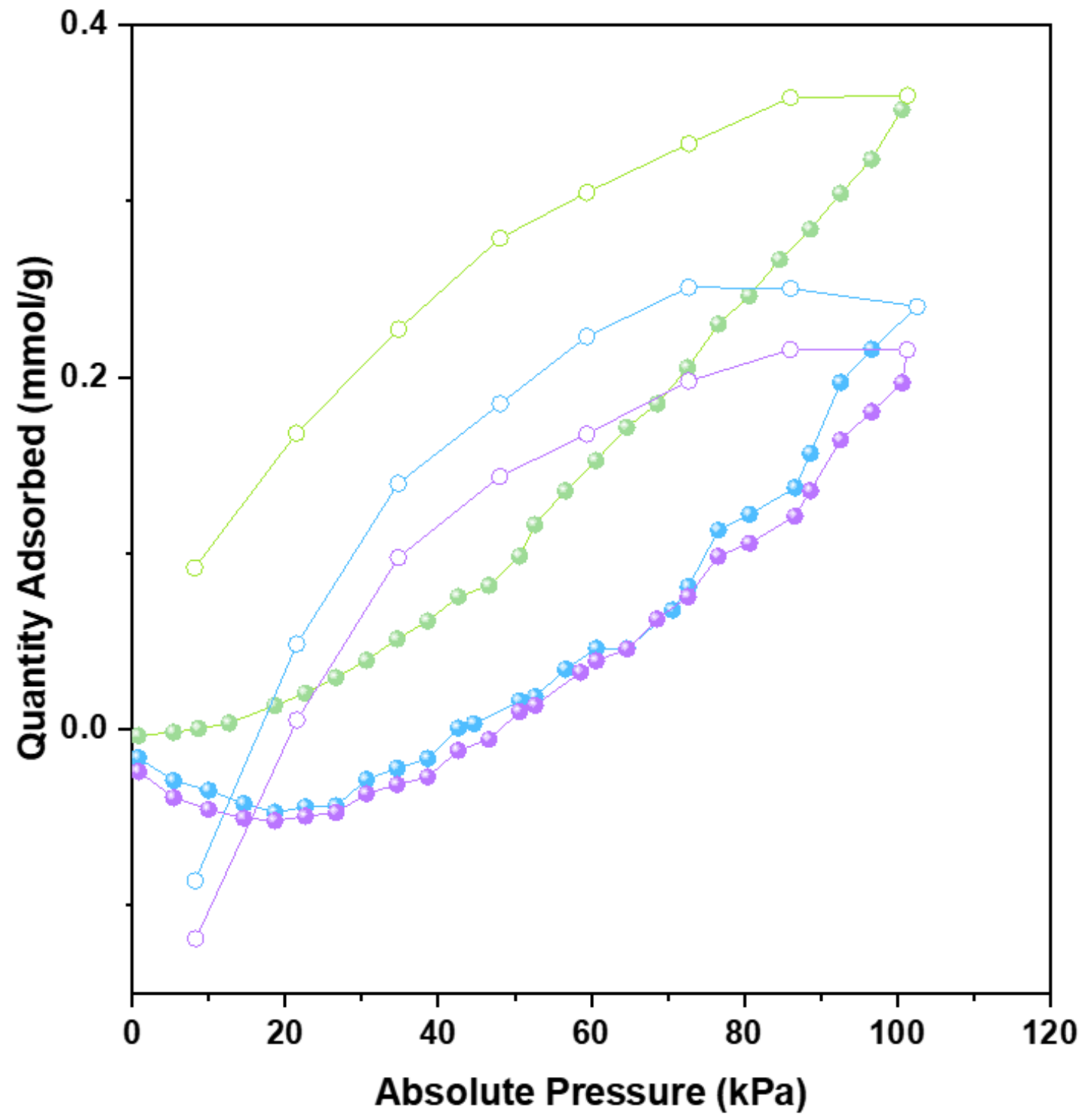


b)

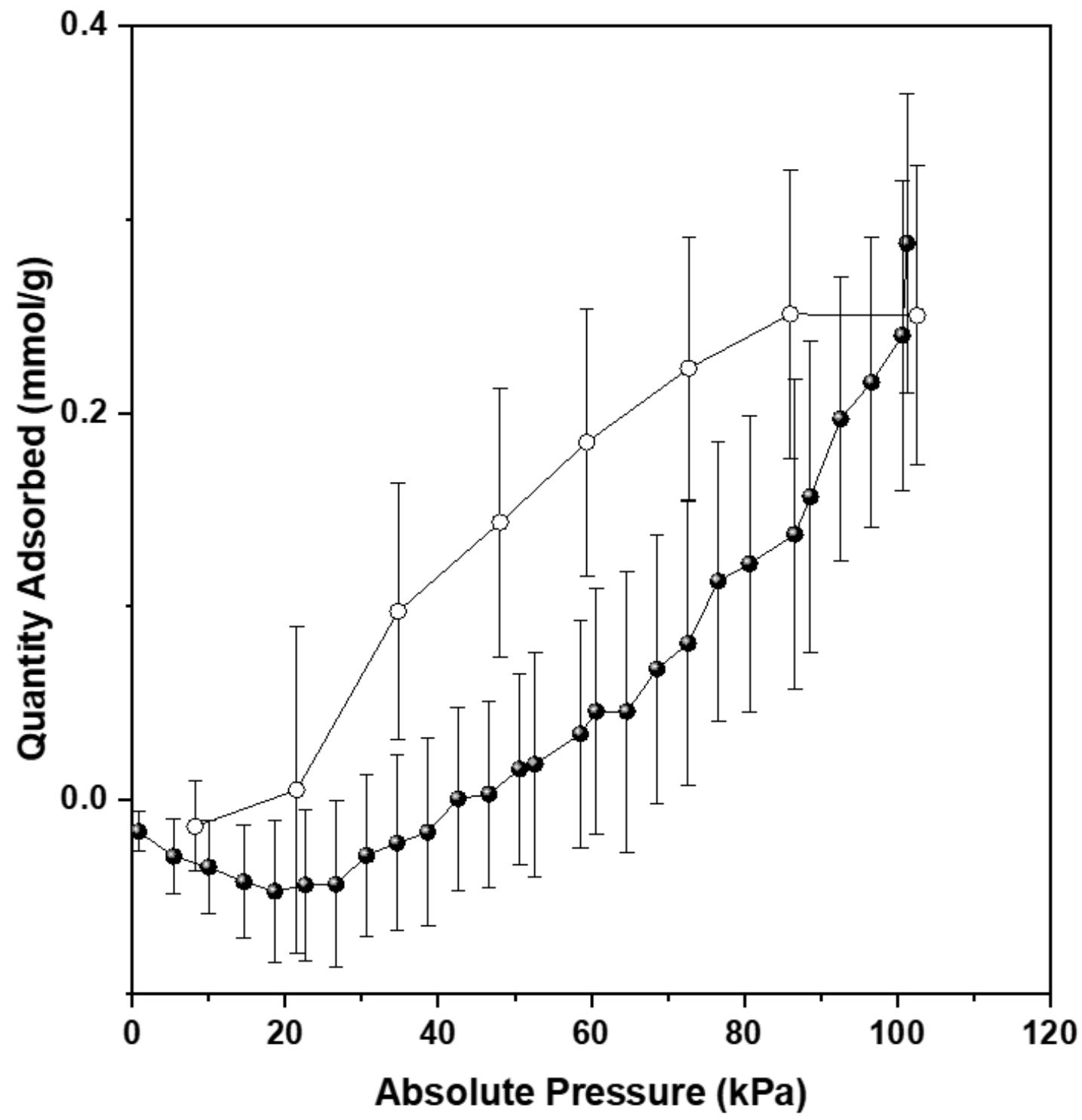


**Figure S54. a)** $CO_2$ adsorption isotherms of three independent samples composed of the physical mixture containing semicrystalline $Me_2L1$ and cooper(II) acetate measured at 298. **b)** $CO_2$ adsorption isotherm showing the average uptake with standard deviation from three independent samples composed of the physical mixture containing semicrystalline $Me_2L1$ and cooper (II) acetate at 298 K. The equilibration time at each pressure step was 200 s.

**a)**

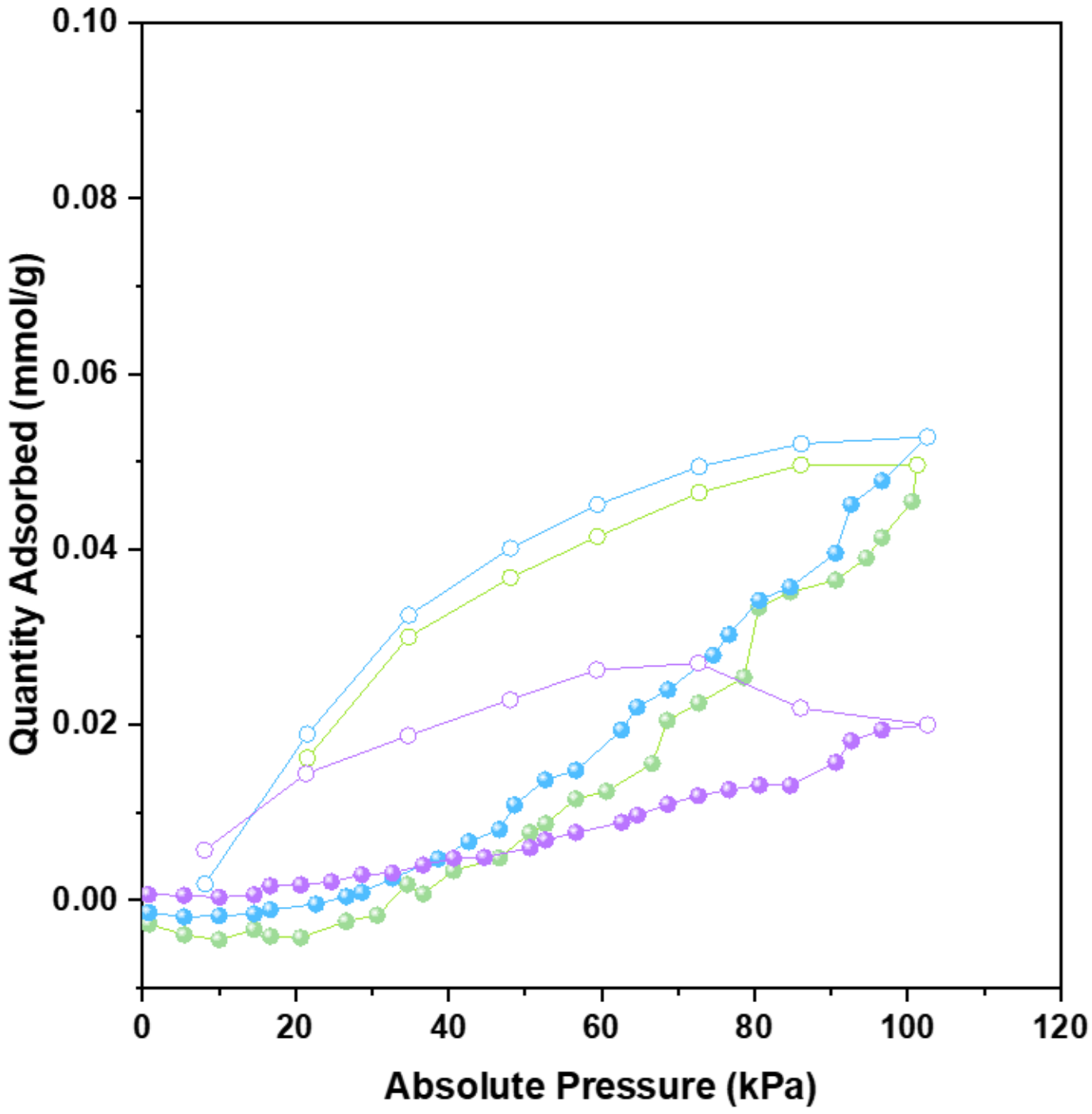


**b)**

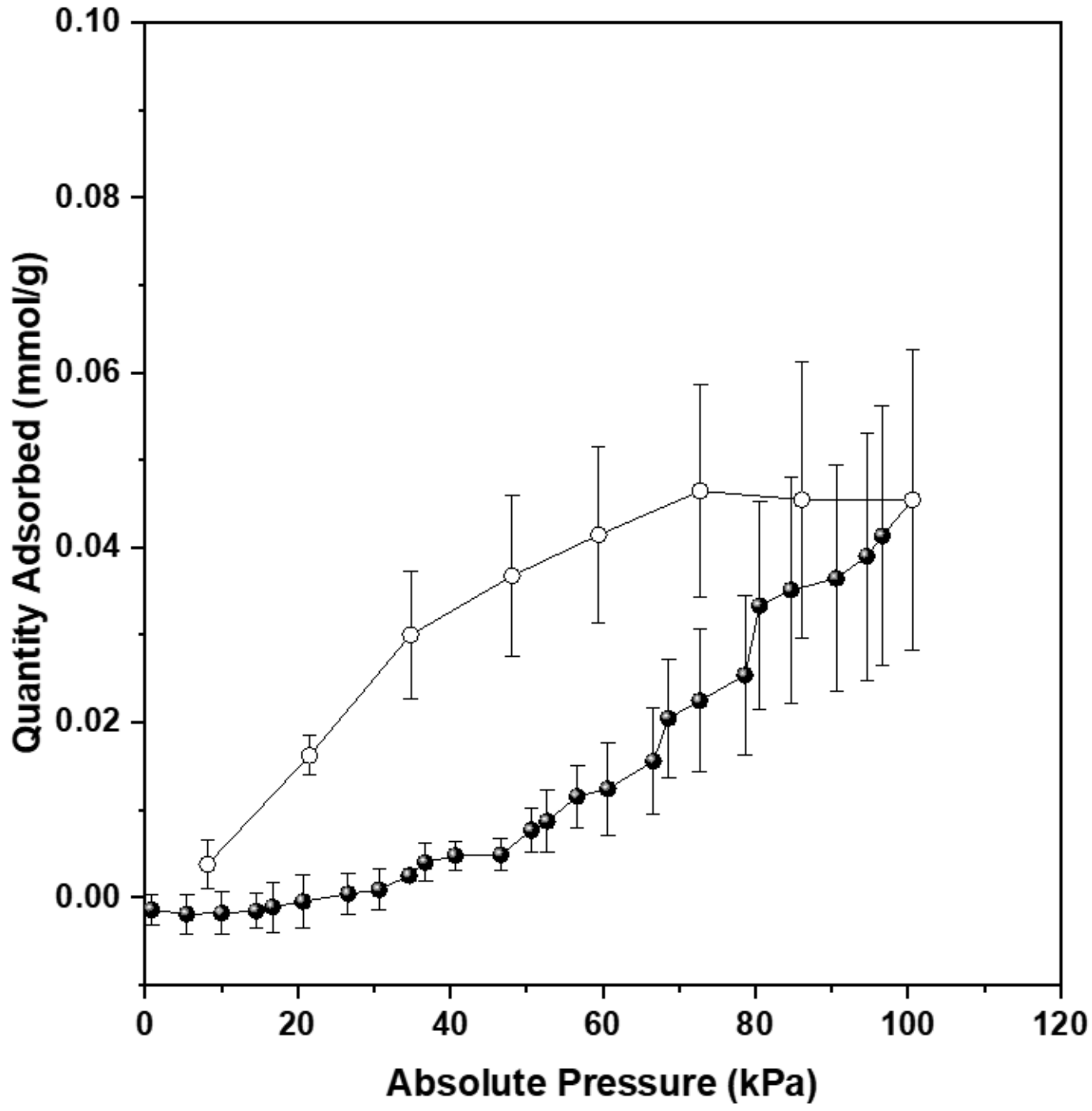


**Figure 55. a)** $CO_2$ adsorption isotherms of three independent samples of $Me_2L1$ measured at 318 K. **b)** $CO_2$ adsorption isotherm showing the average uptake with standard deviation from three independent samples composed of $Me_2L1$ at 318 K. The equilibration time at each pressure step was 200 s.

a)

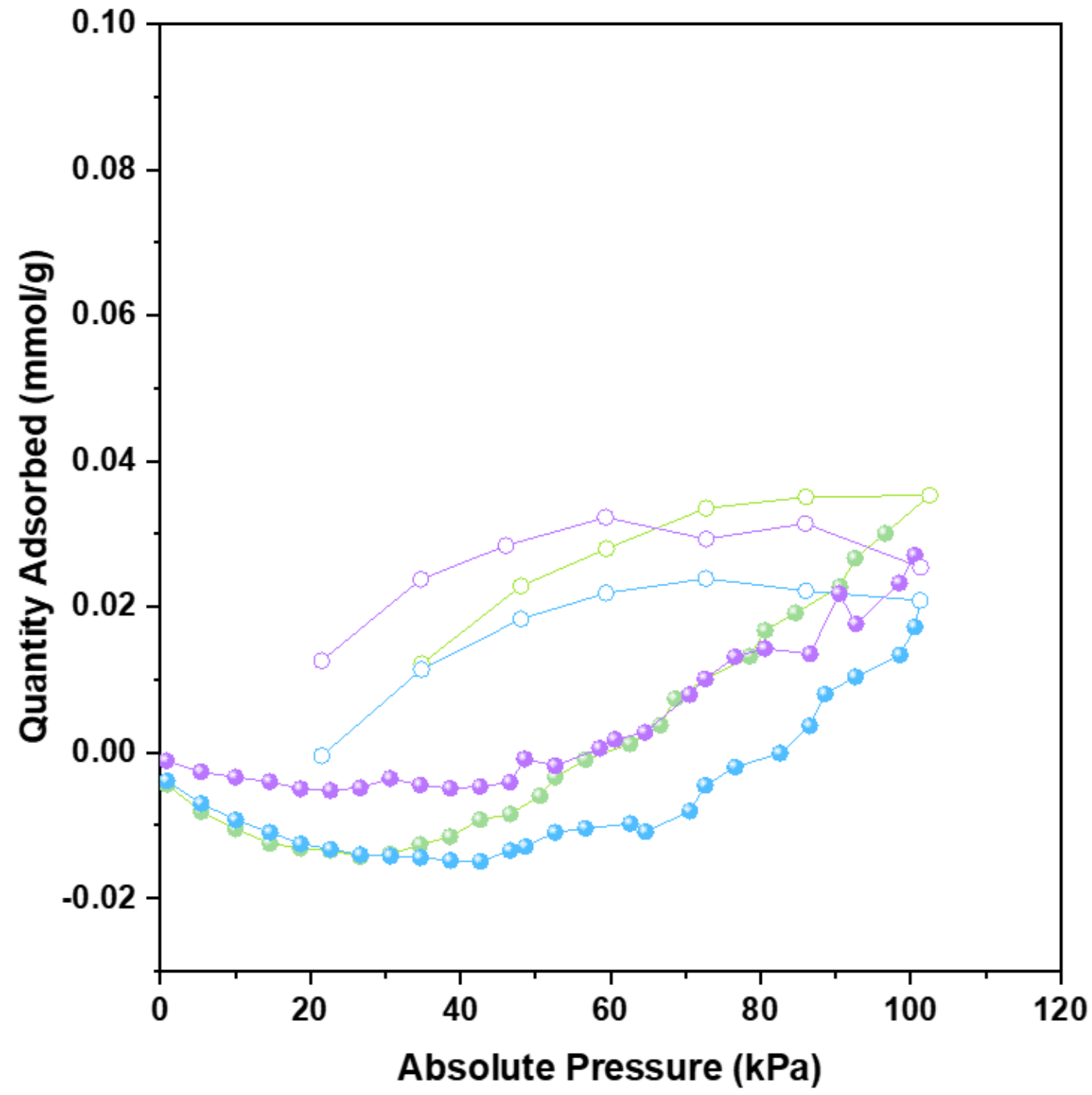


b)

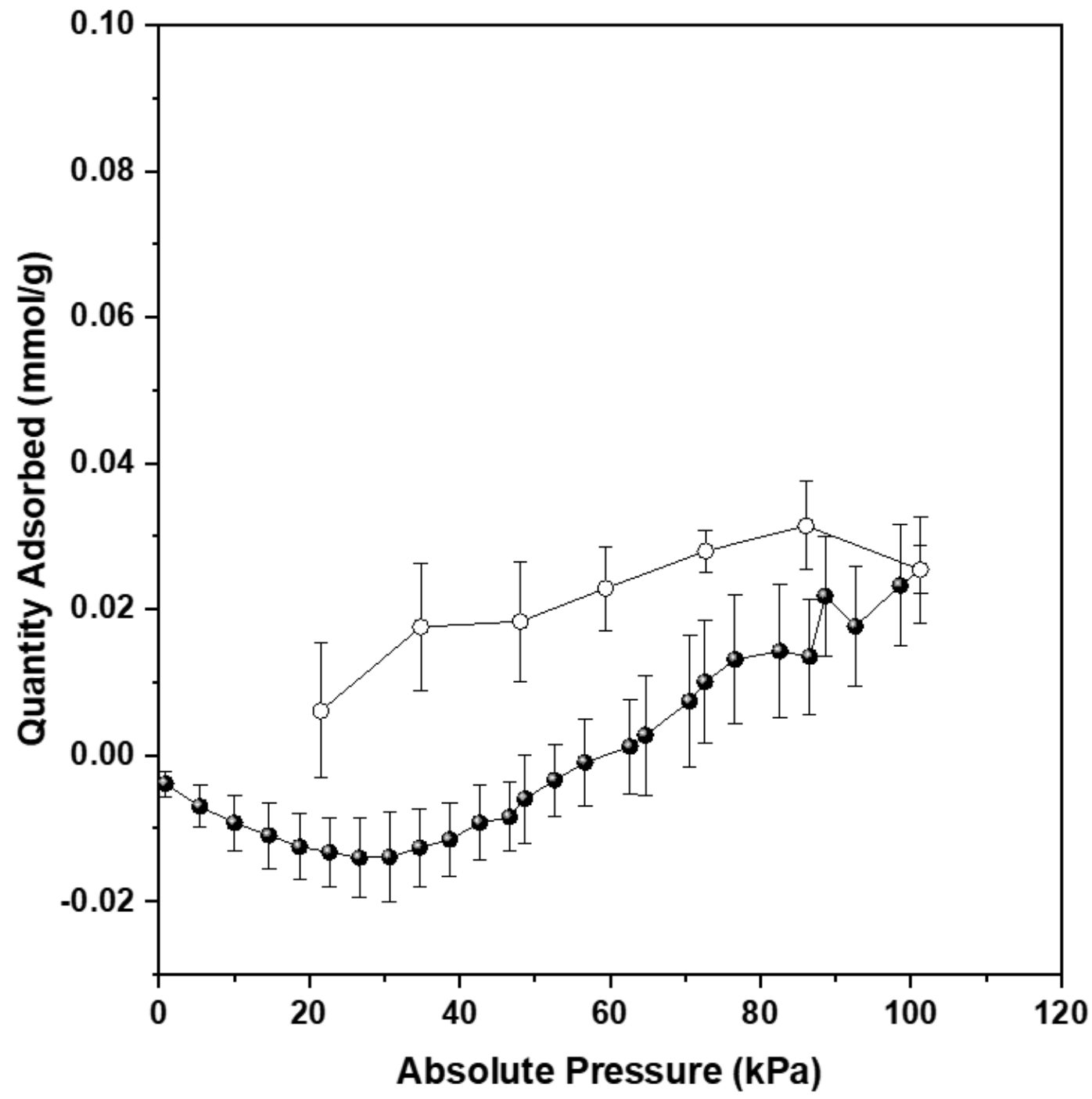


**Figure S56. a)** $CO_2$ adsorption isotherms of three independent samples composed of the physical mixture containing semicrystalline $Me_2L1$ and rhodium (II) acetate measured at 318 K. **b)** $CO_2$ adsorption isotherm showing the average uptake with standard deviation from three independent samples composed of the physical mixture containing semicrystalline $Me_2L1$ and rhodium (II) acetate at 318 K. The equilibration time at each pressure step was 200 s.

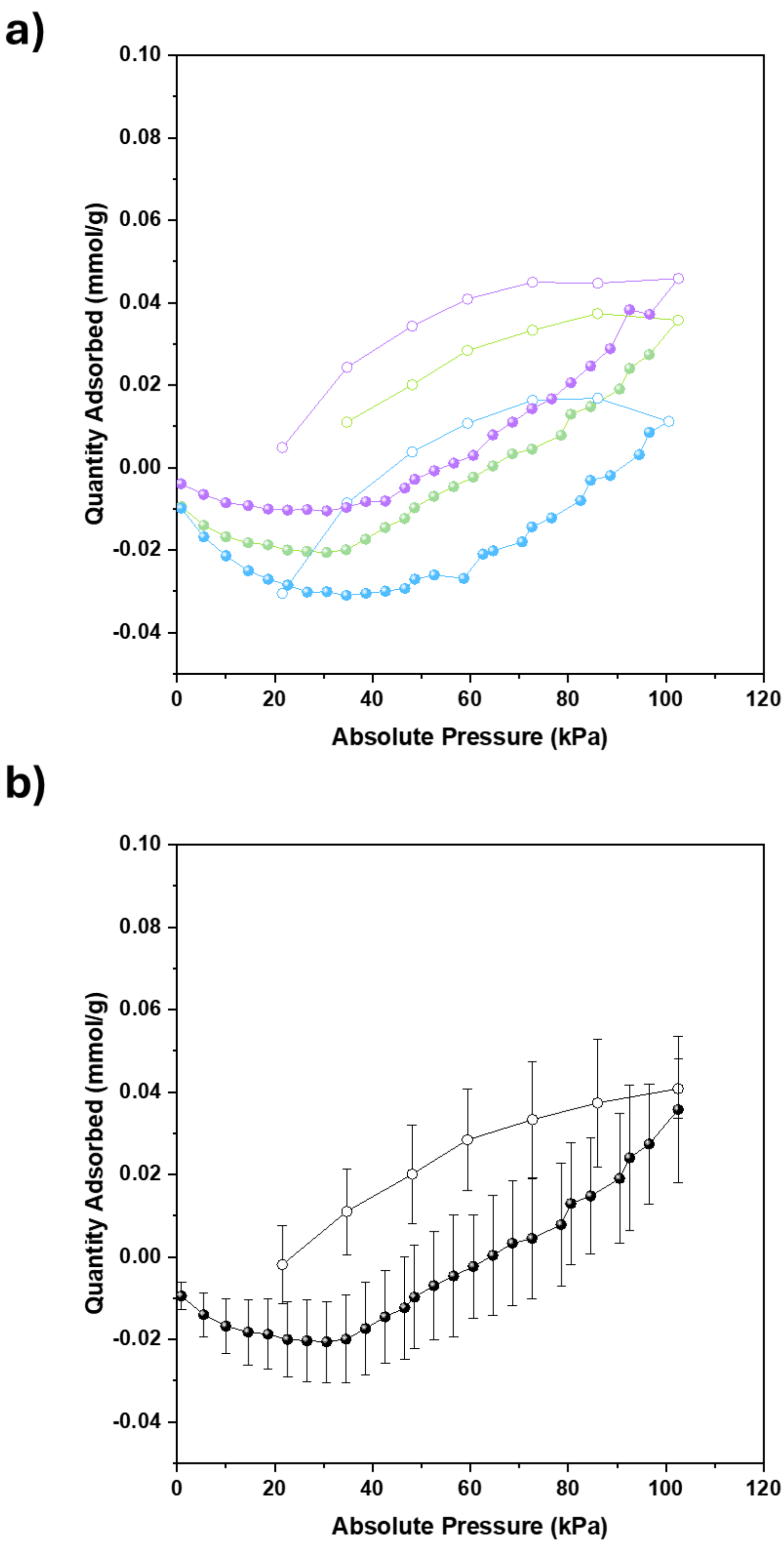


**Figure S57. a)** $CO_2$ adsorption isotherms of three independent samples composed of the physical mixture containing semicrystalline $Me_2L1$ and cooper(II) acetate measured at 318 K. **b)** $CO_2$ adsorption isotherm showing the average uptake with standard deviation from three independent samples composed of the physical mixture containing semicrystalline $Me_2L1$ and cooper (II) acetate at 318 K. The equilibration time at each pressure step was 200 s.

### S3.7. Scale-up synthesis of Cu-BCN-5

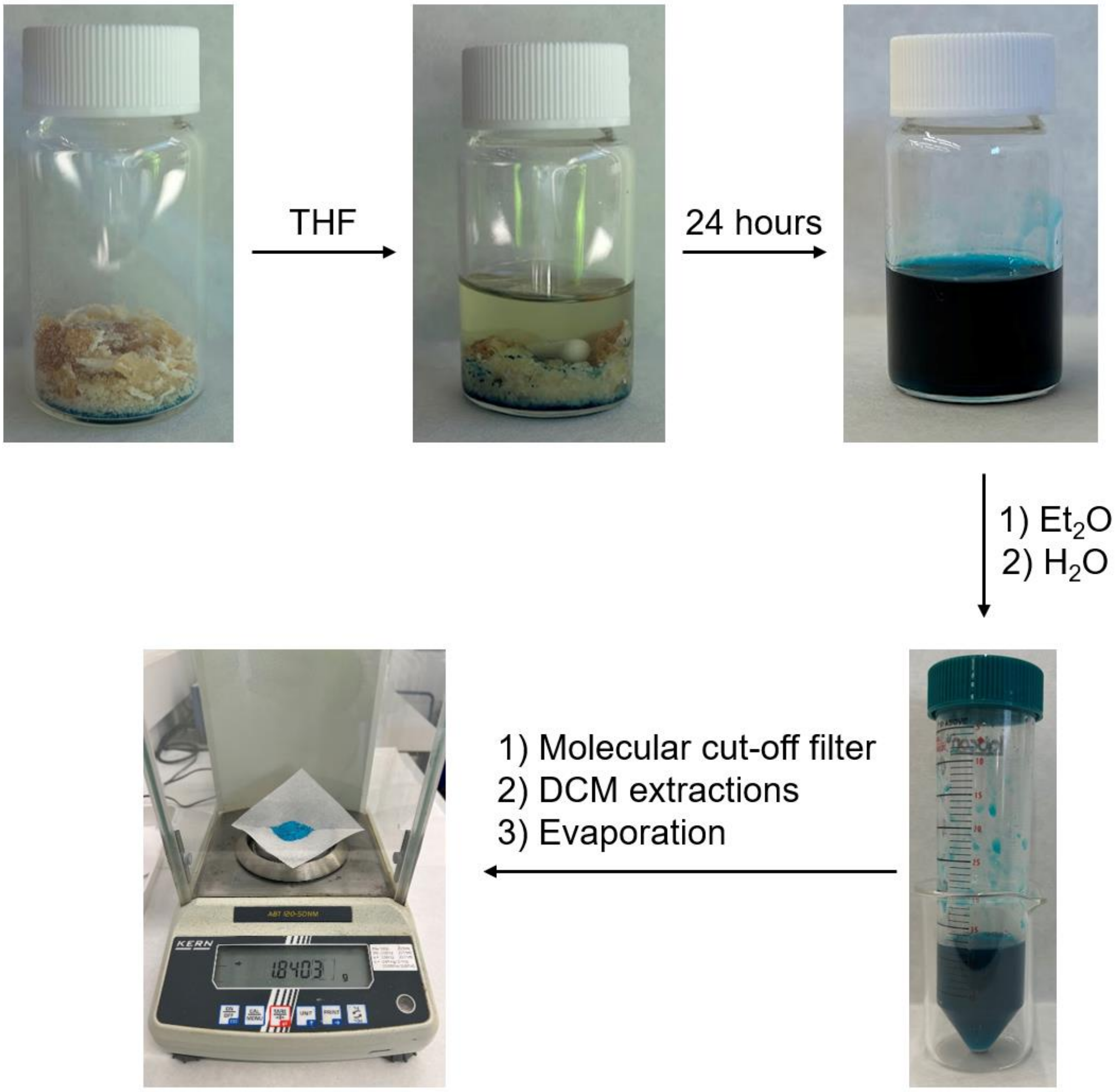


**Figure S58.** Photographs illustrating the scale-up synthesis to fabricate 1.8403 g of Cu-BCN-5.

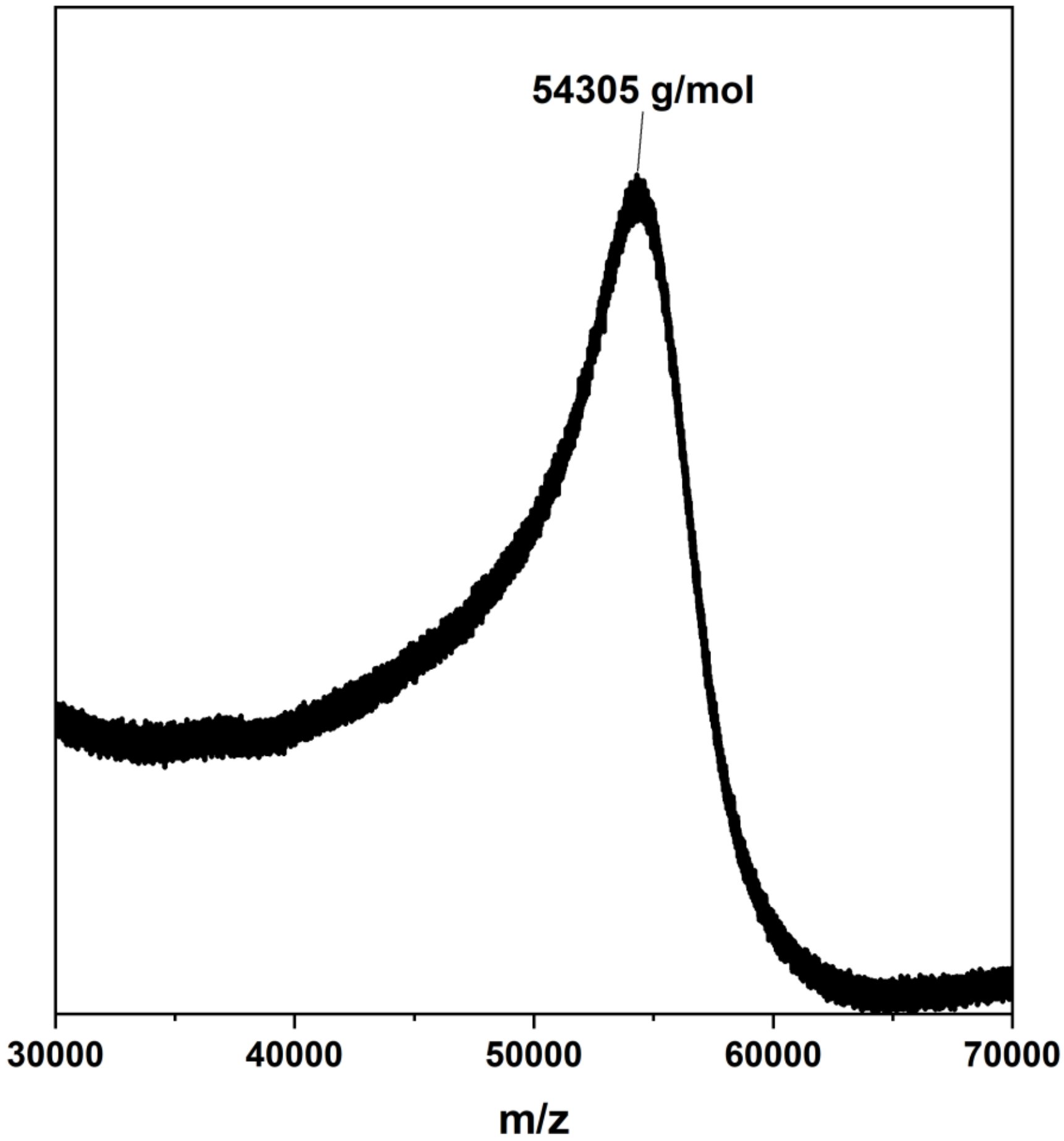


**Figure S59.** MALDI-TOF spectrum of scale-up Cu-BCN-5. The highlighted mass corresponds to the molecular formula of $[Cu_{24}(PEG\text{-}bdc)_{24} + H^+]^+$ (expected m/z = 51973 ± 4800 g·mol$^{-1}$; found m/z = 54305 g·mol$^{-1}$).

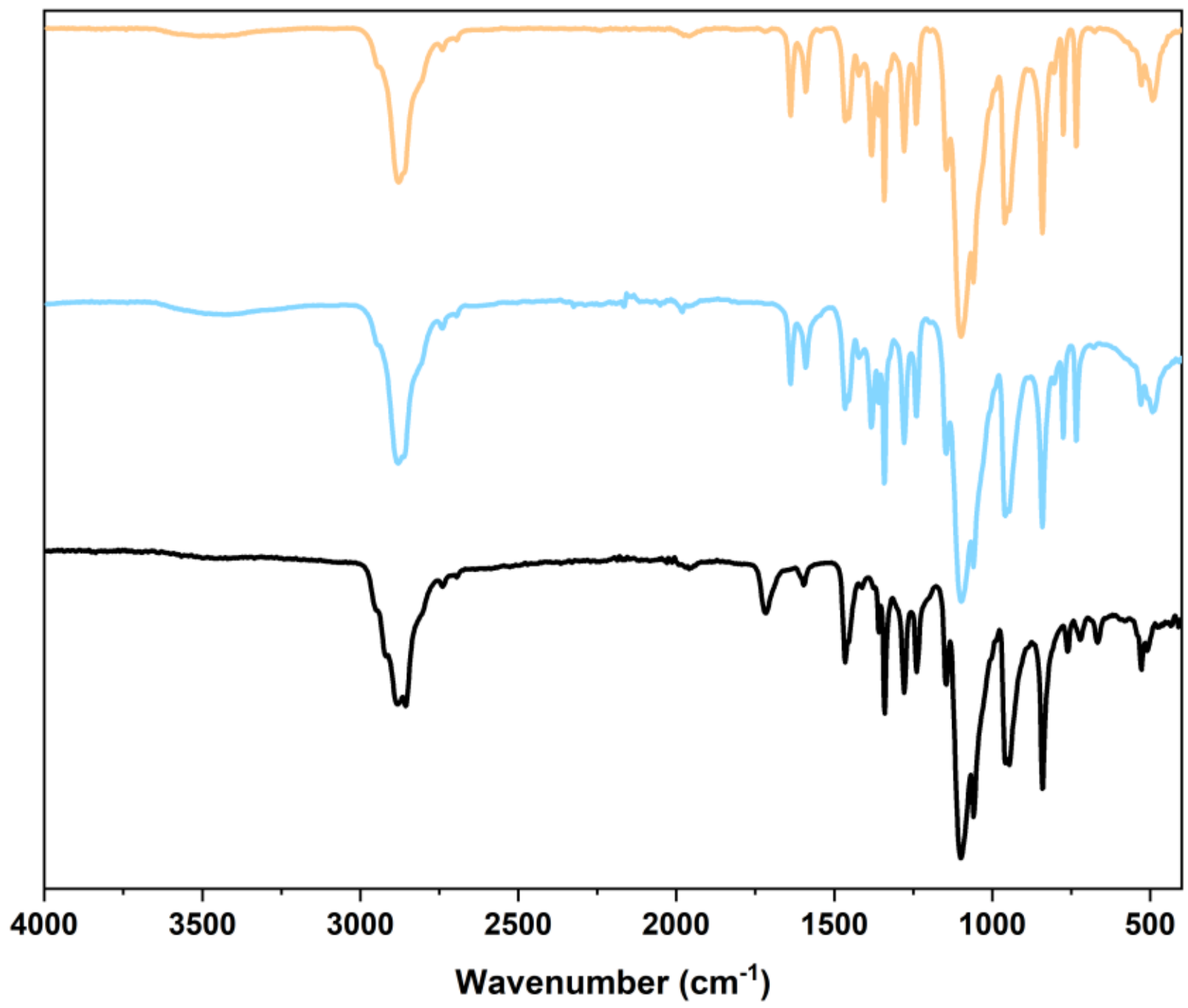


**Figure S60.** FTIR spectra of $H_2L1$ (black, bottom), as-made Cu-BCN-5 (blue, middle) and scale-up Cu-BCN (orange, top).

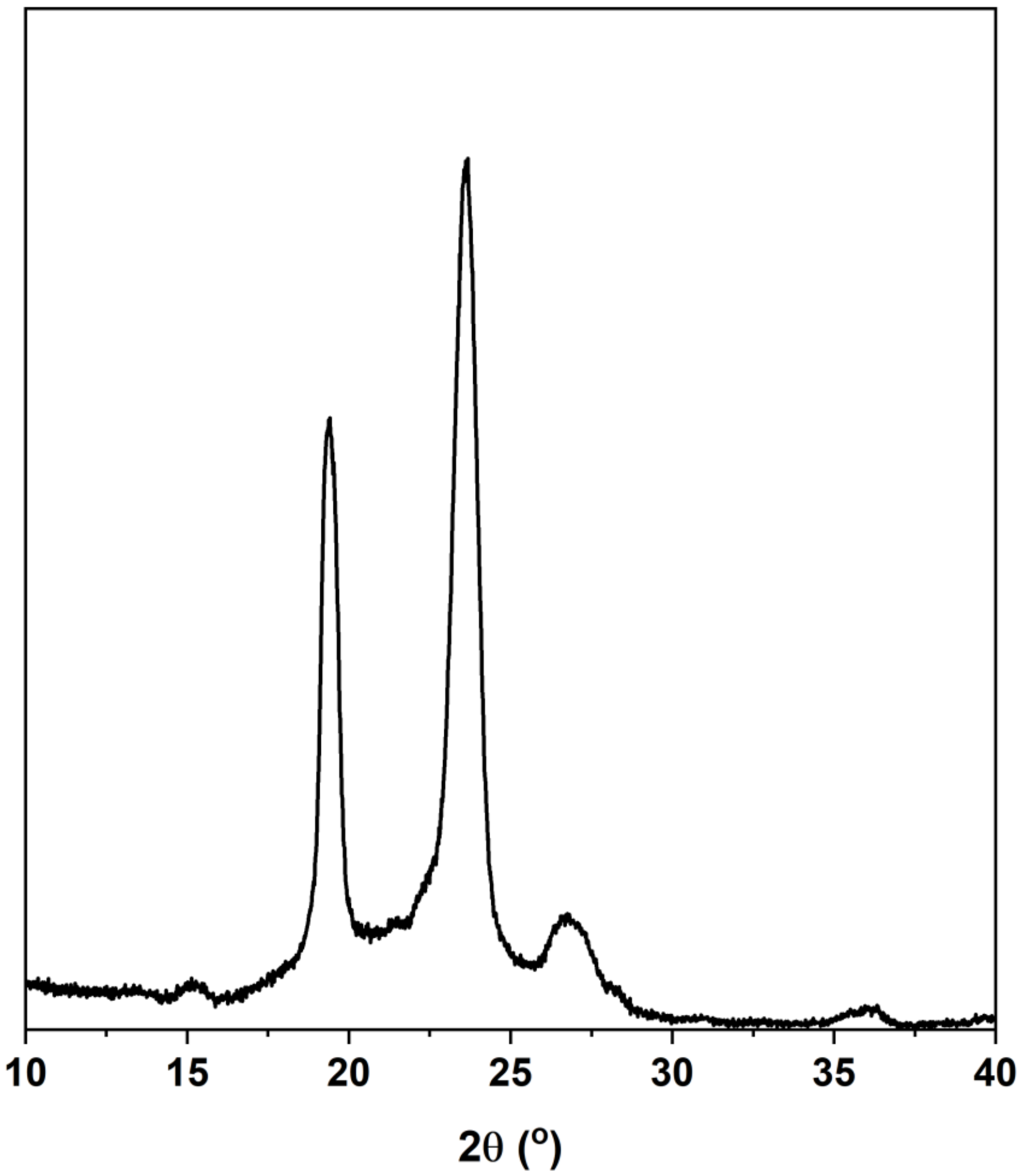


**Figure S61.** PXRD pattern of scale-up Cu-BCN-5 at room temperature.

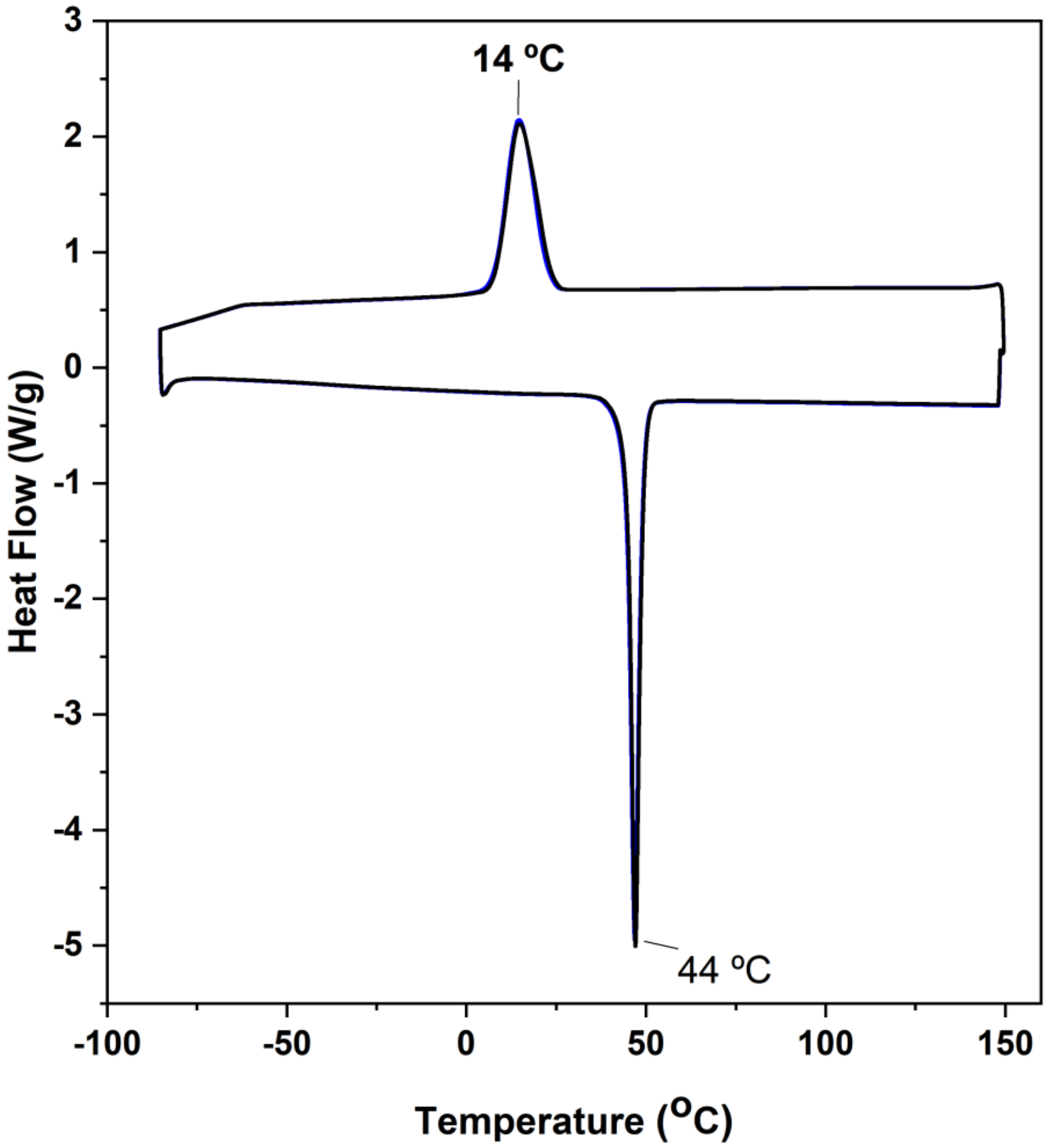


**Figure S62.** DSC curves of scale-up Cu-BCN-5, showing two consecutive runs.

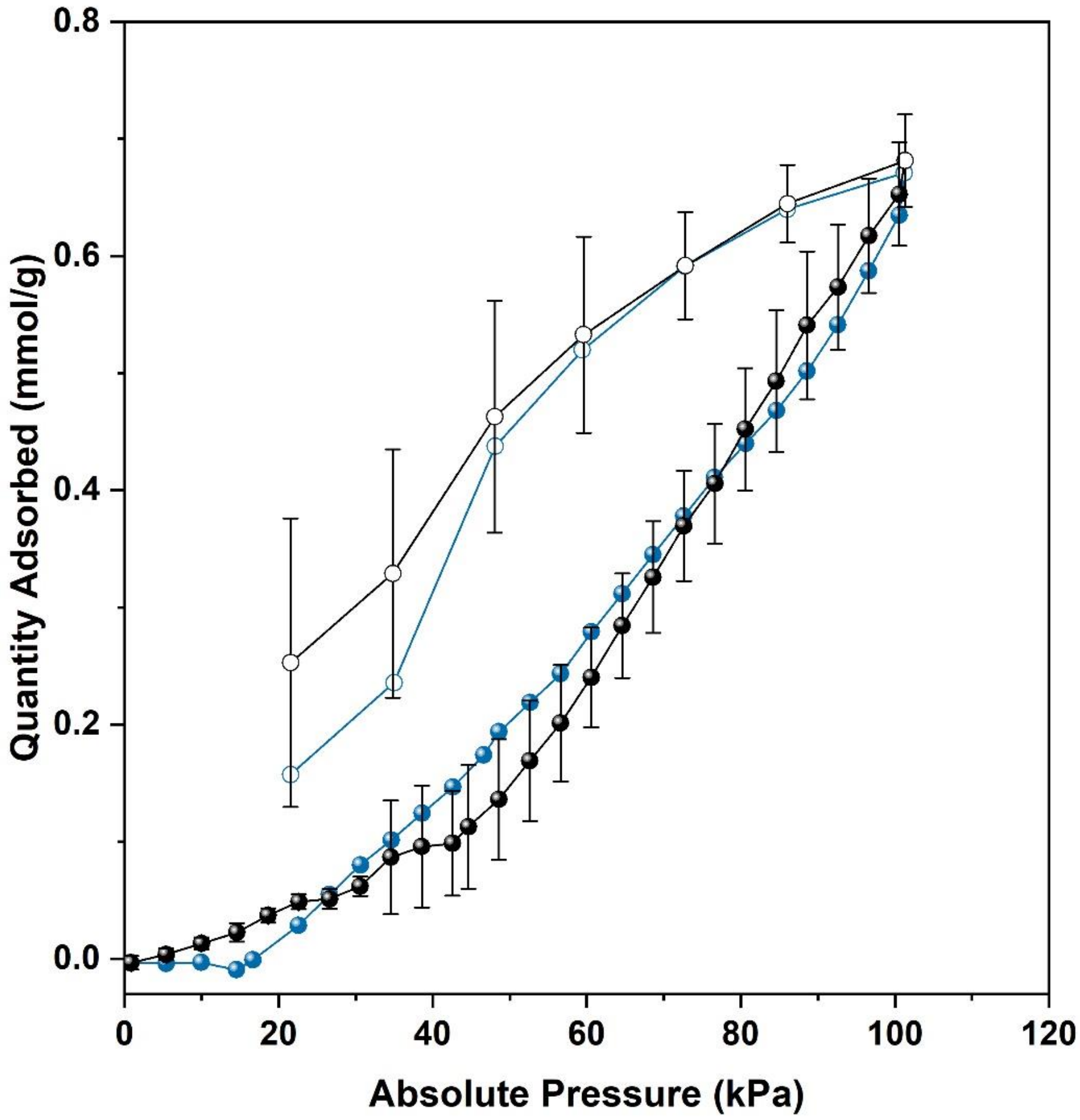


**Figure S63.** $CO_2$ adsorption comparison of Cu-BCN-5 (black) and scale-up Cu-BCN-5 (blue).

## S3.8. Shaping

### S3.8.1. Cu-BCN-5 disk

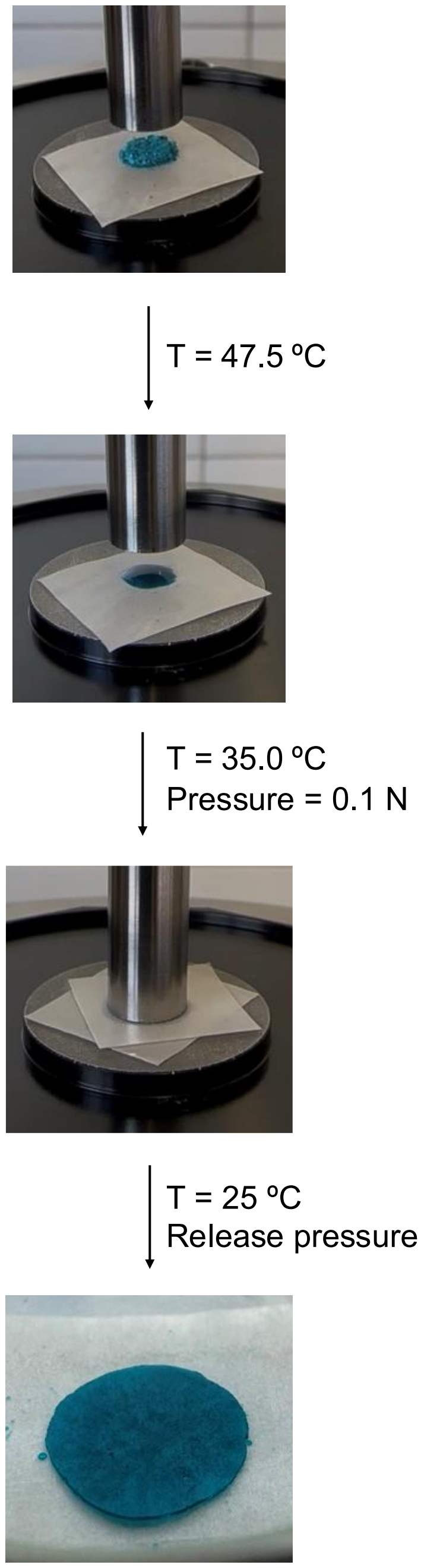


**Figure S64.** Photographs illustrating the hot-pressing method used to fabricate Cu-BCN-5 disks.

a)

b)

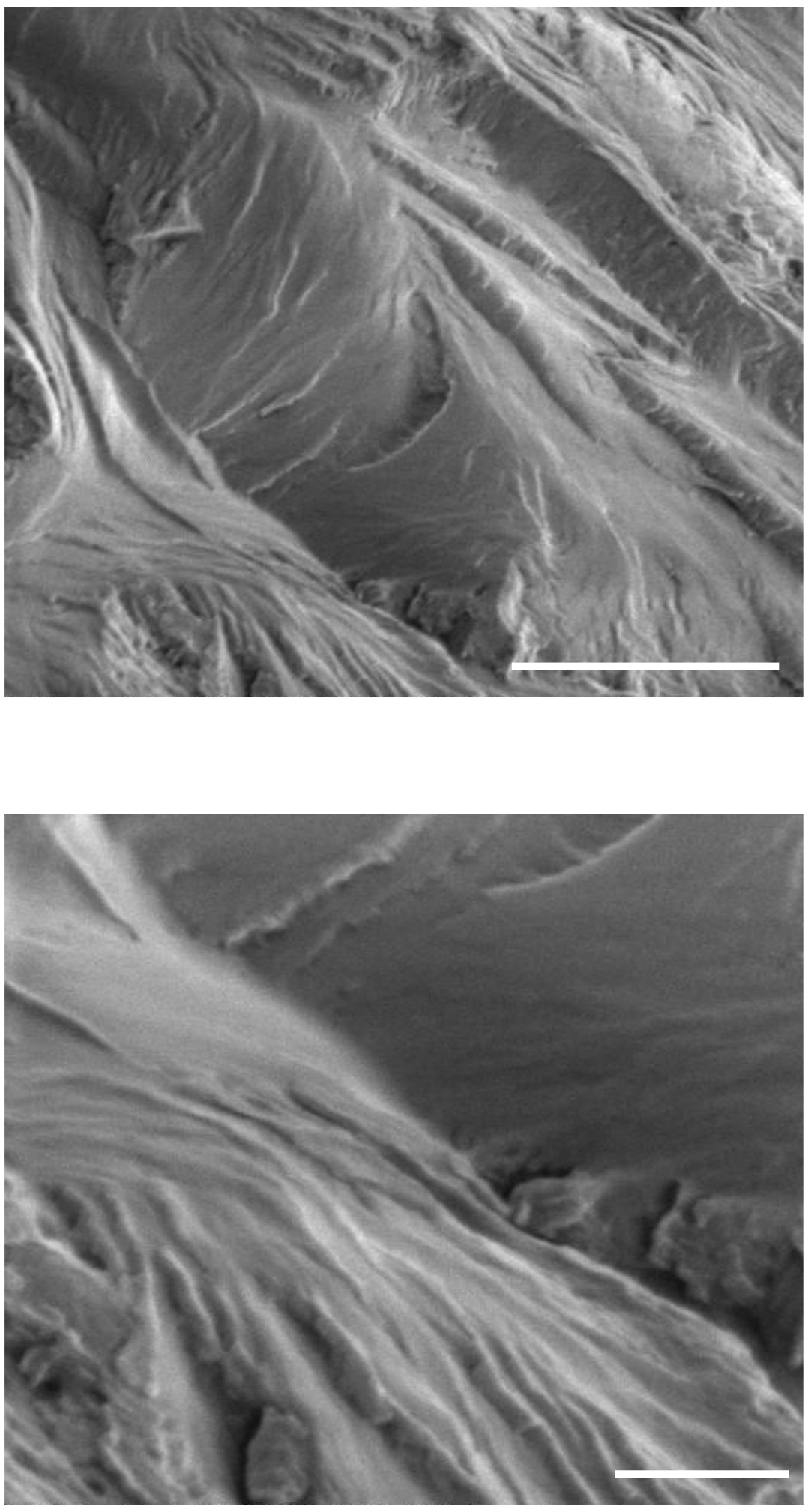

**Figure S65.** Close FE-SEM images of the surface of Cu-BCN-5 disk. Scale bars: 4 µm (a) and 1 µm (b).

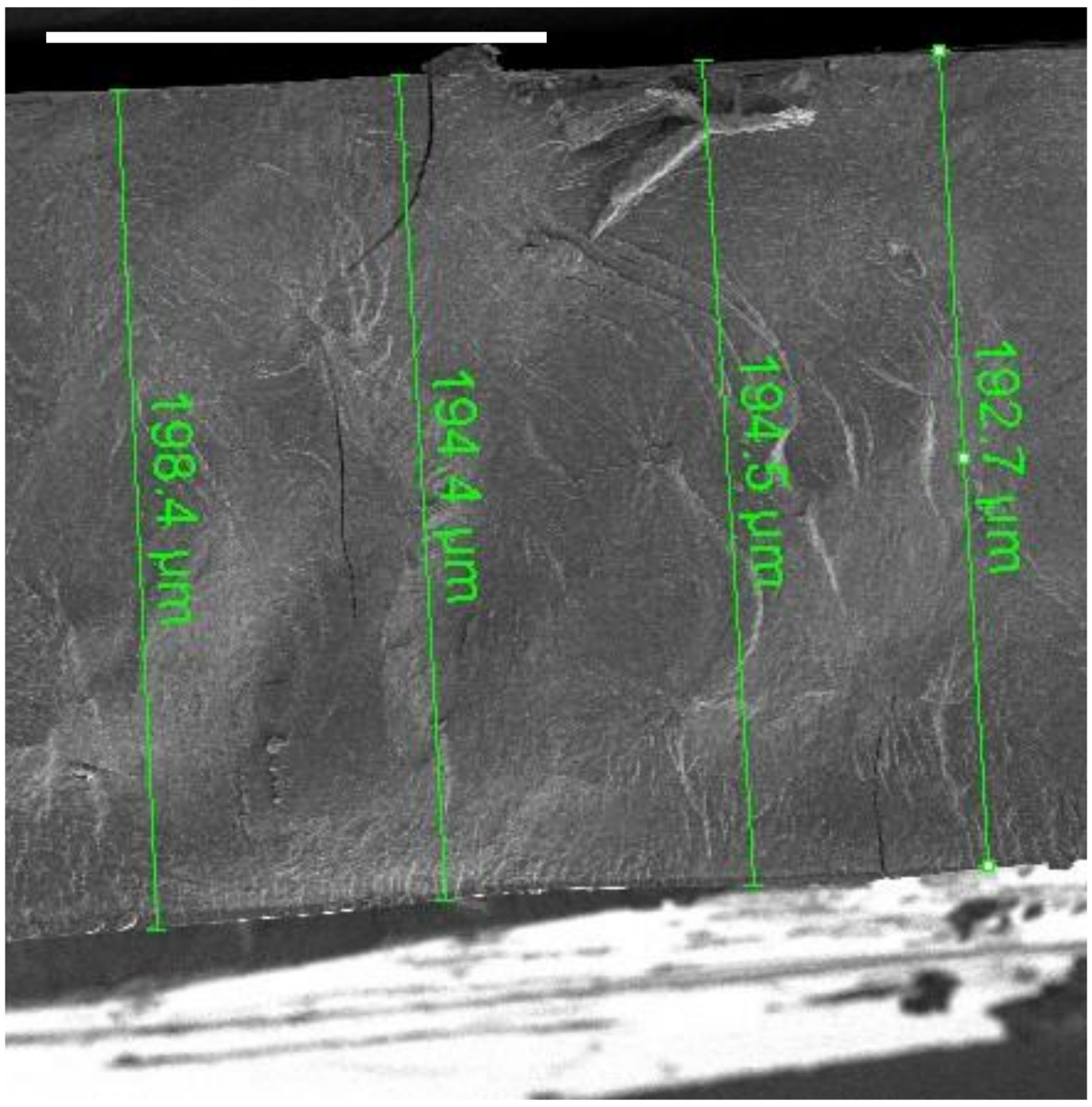


**Figure S66.** Cross-sectional FE-SEM images of the Cu-BCN-5 disk Scale bar = 100 µm

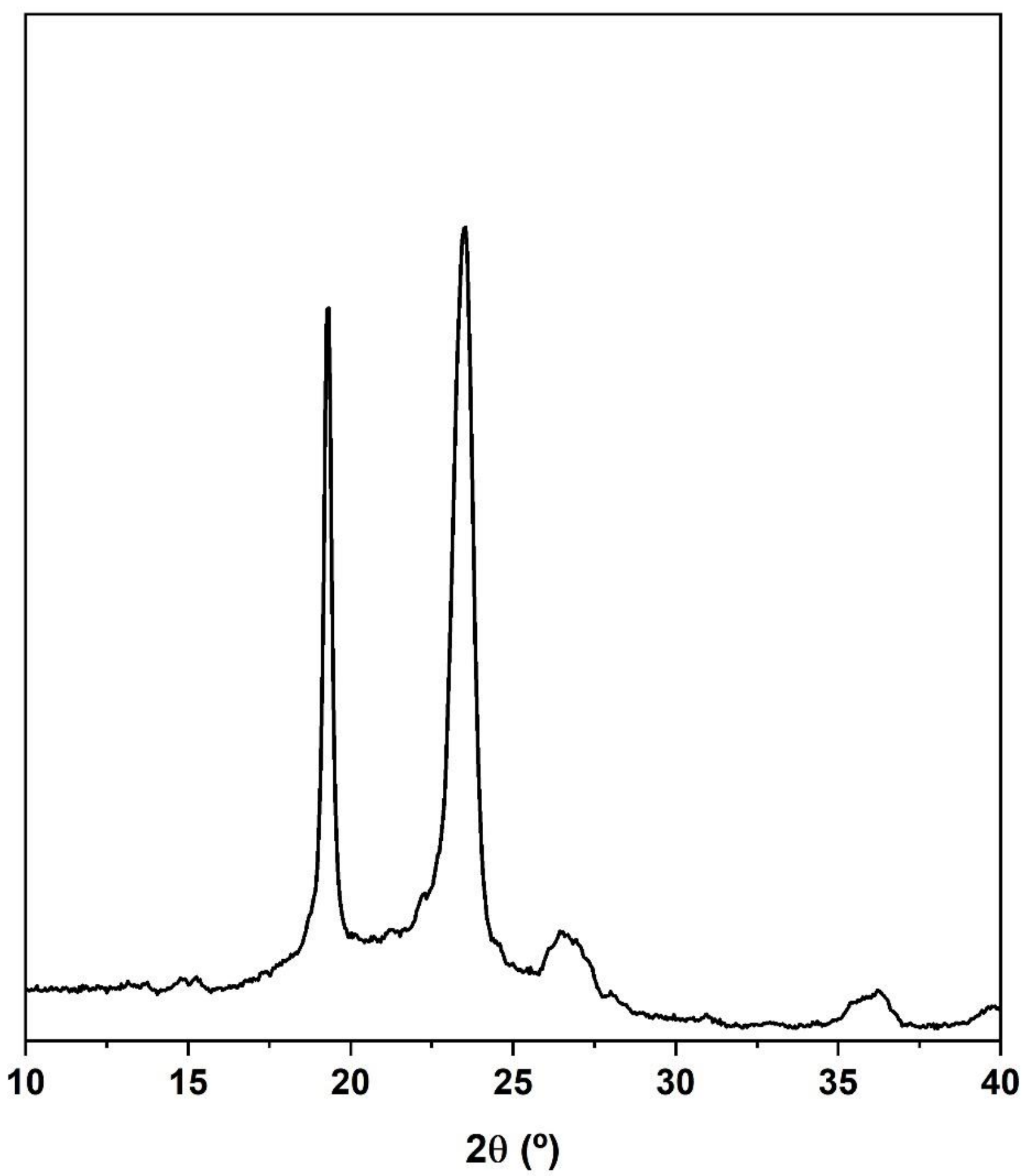


**Figure S67.** PXRD pattern of Cu-BCN-5 disk.

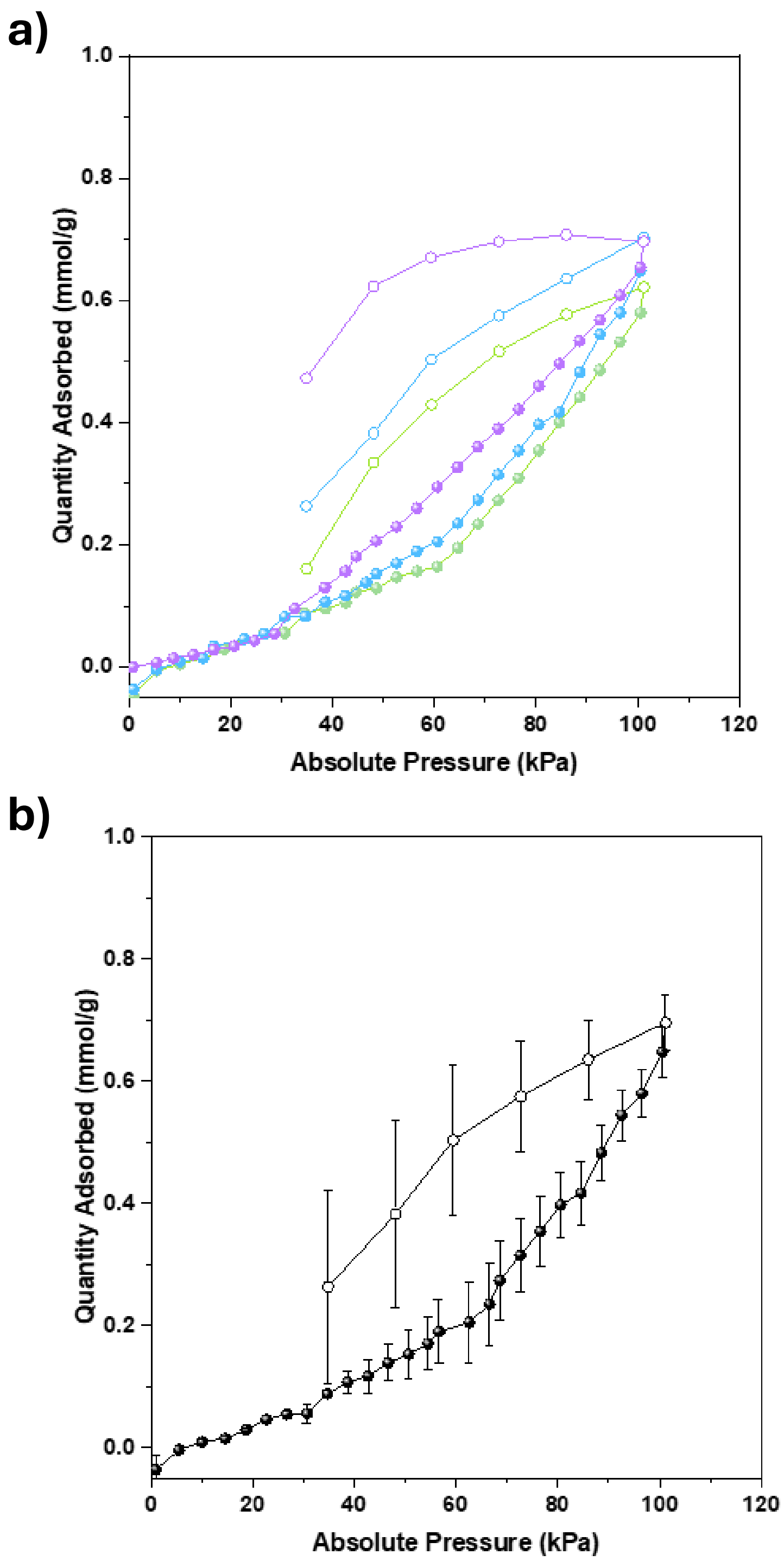


**Figure S68. a)** $CO_2$ adsorption isotherms of three independent Cu-BCN-5 disks measured at 298 K. b) $CO_2$ adsorption isotherm showing the average uptake with standard deviation from three independent Cu-BCN-5 disks at 298 K. The equilibration time at each pressure step was 200 s.

**S3.8.2. Cu-BCN-5 monolith**

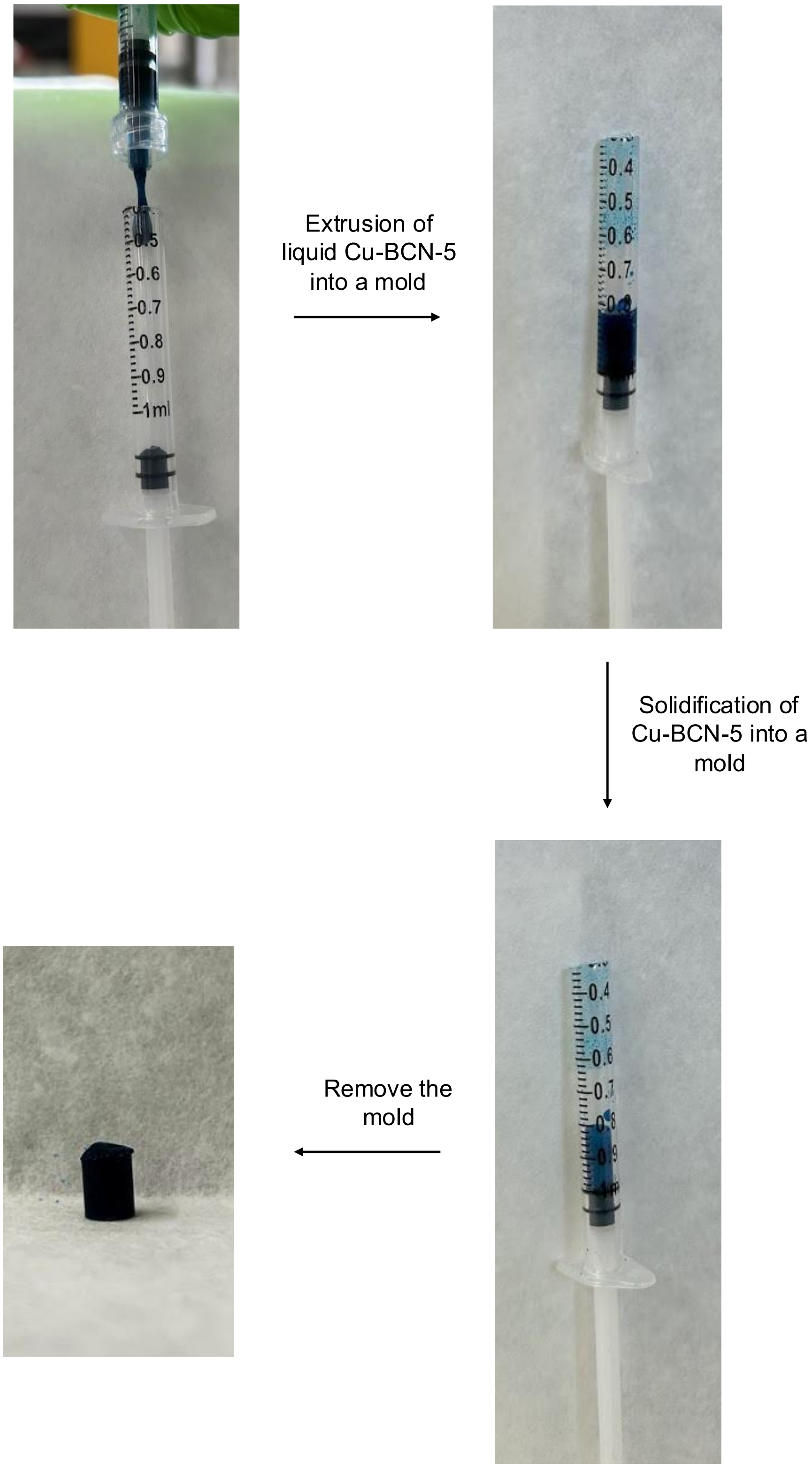


**Figure S69.** Photographs illustrating the extrusion method used to fabricate Cu-BCN-5 monolith.

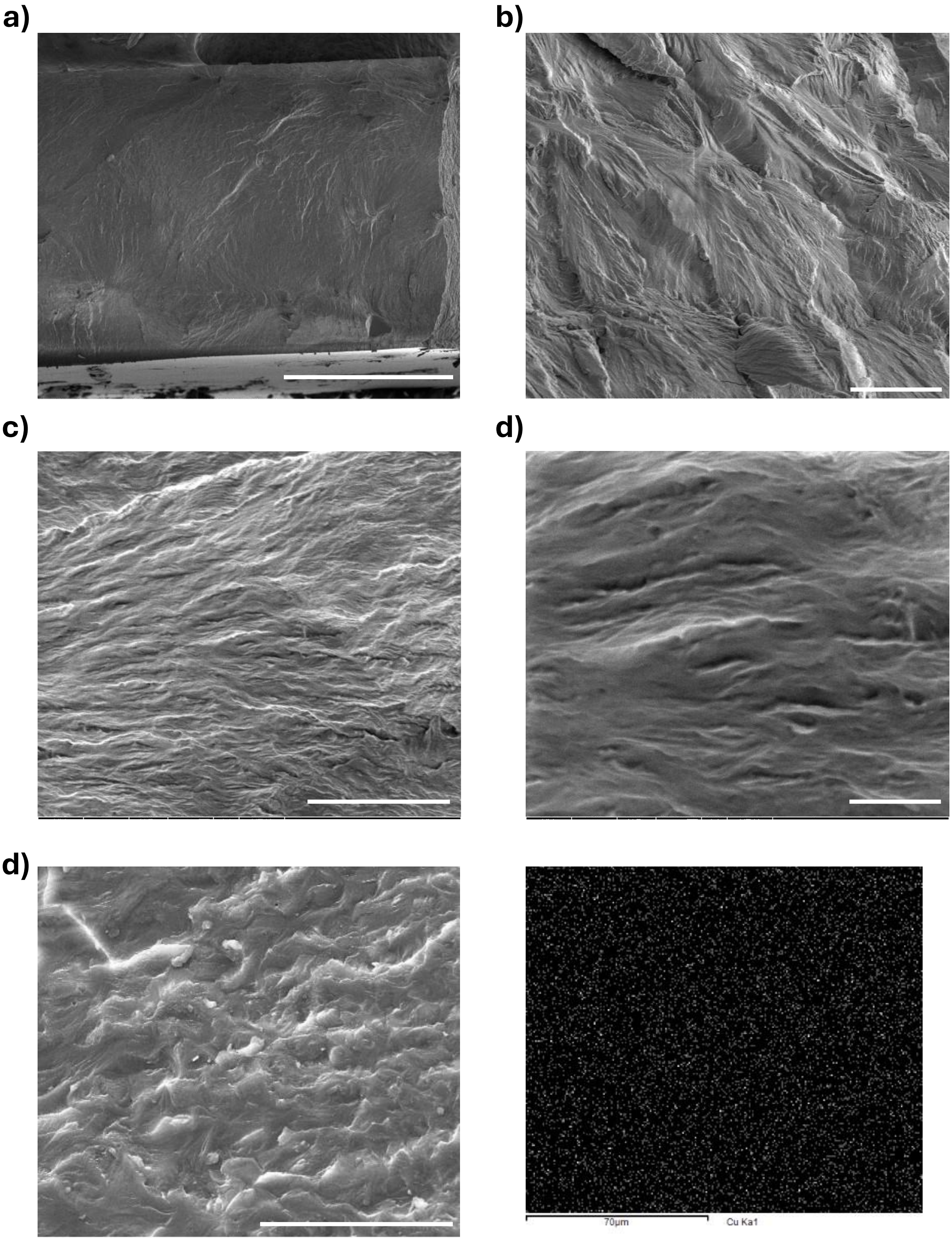


**Figure S70.** FE-SEM images of Cu-BCN-5 monolith. **a)** Inner surface exposed by mechanically fracturing the monolith. **b-d)** Higher magnification images of the fractured surface. Scale bar = 100 µm (a), 10 µm (b), 5 µm (c) and 1 µm (d). **e)** SEM-EDX analysis of Cu-BCN-5 monolith showing the distribution of Cu. Scale bar = 70 µm.

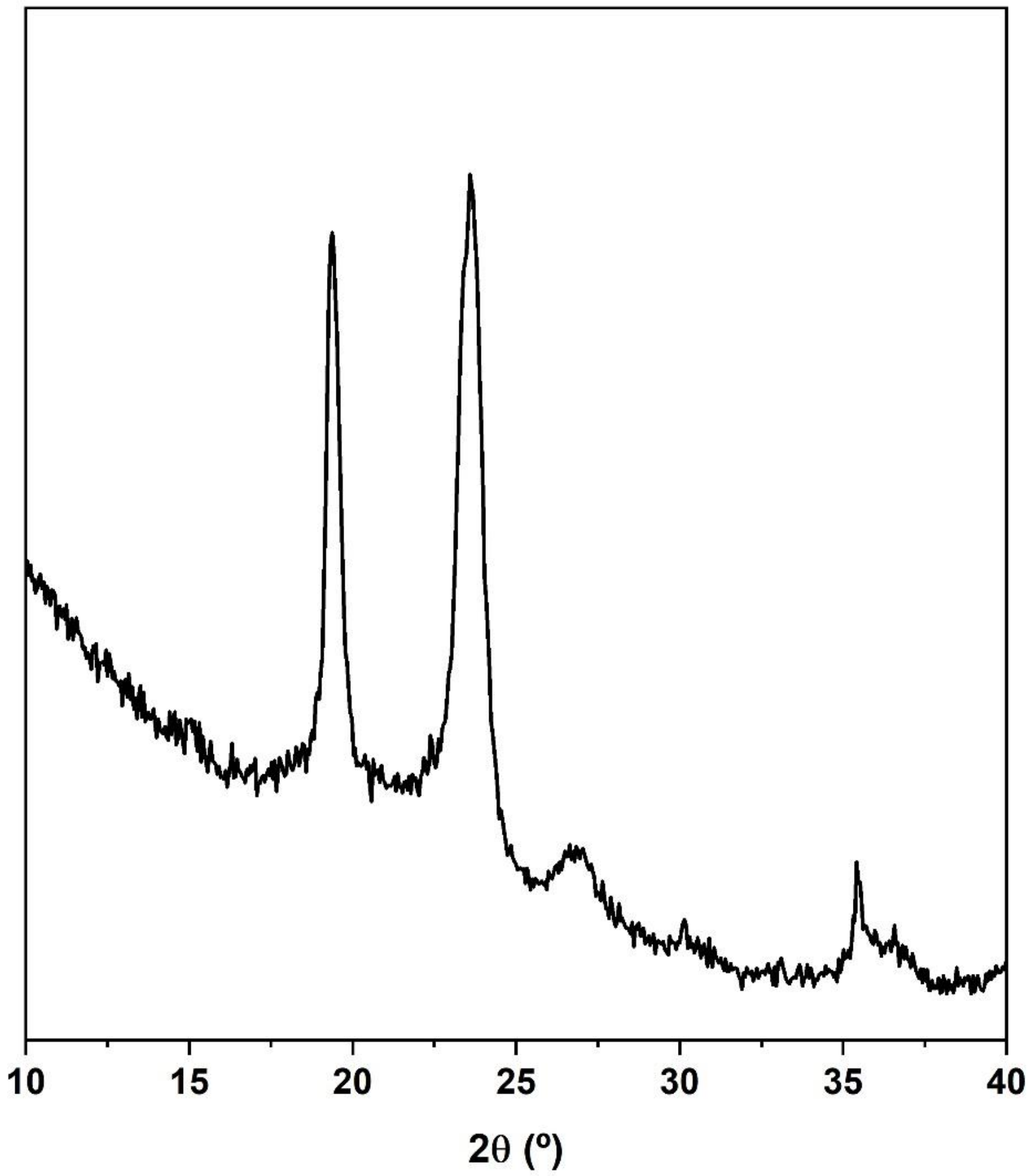


**Figure S71.** PXRD pattern of Cu-BCN-5 monolith.

a)

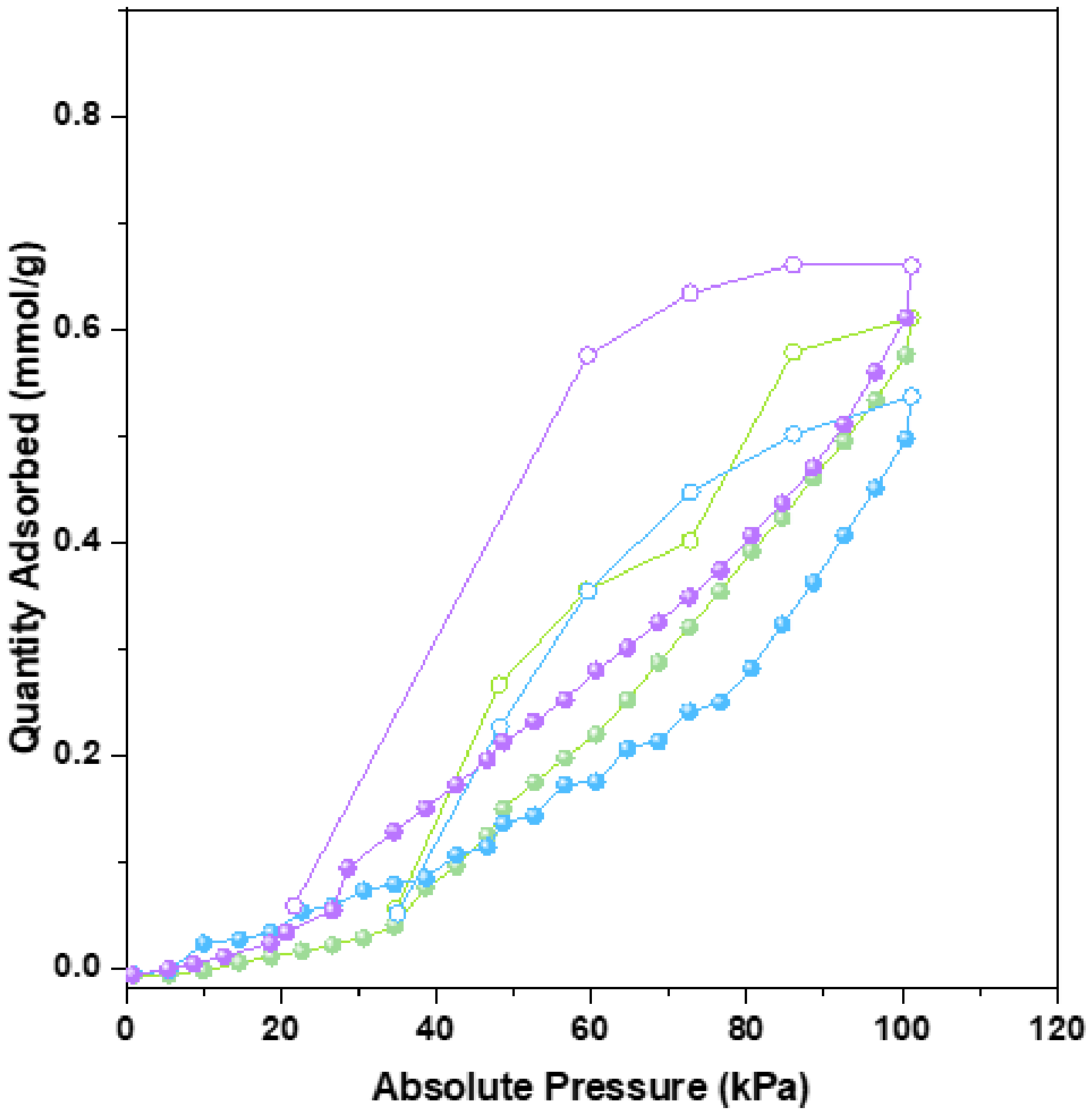


b)

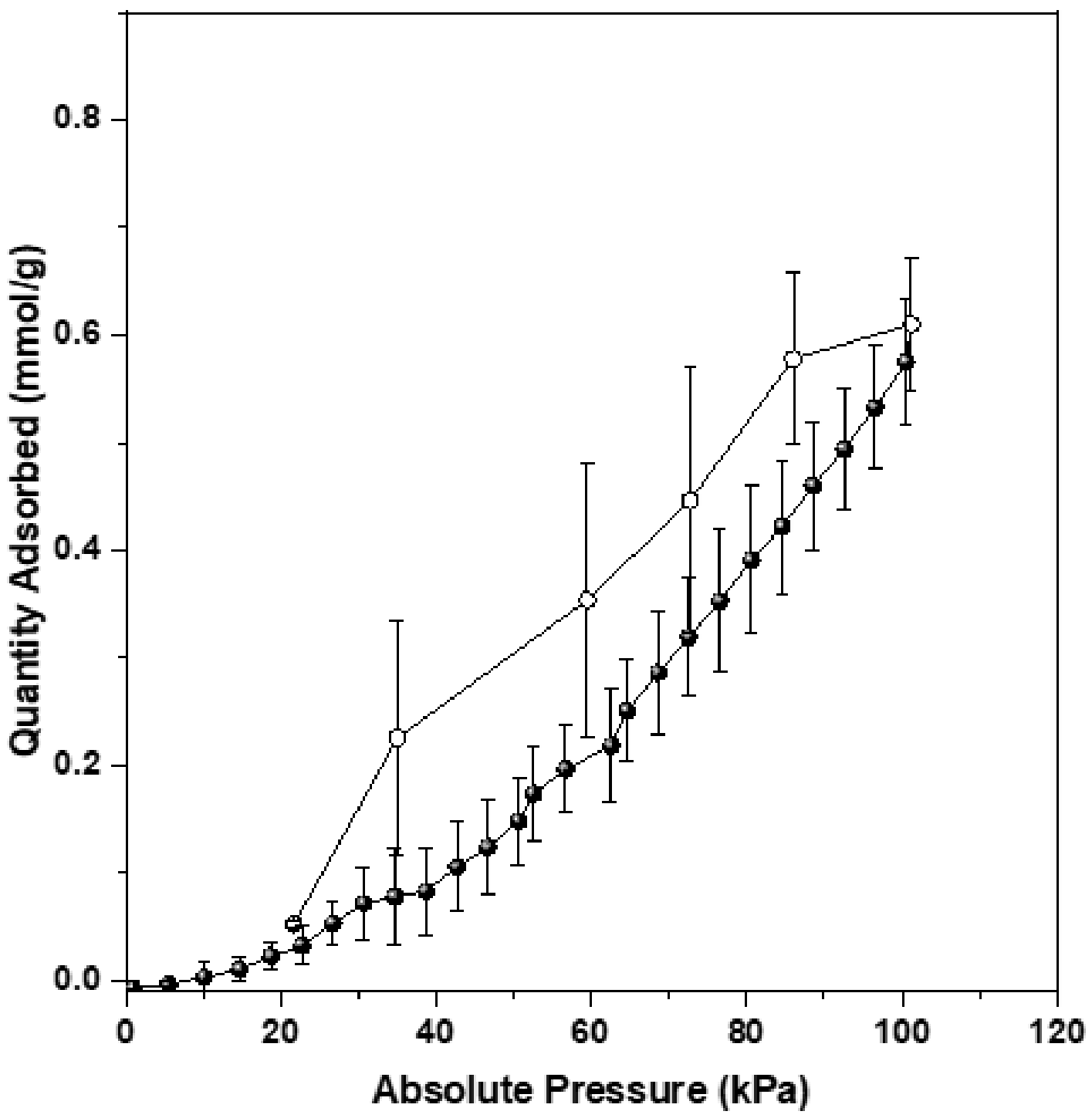


**Figure S72. a)** $CO_2$ adsorption isotherms of three independent Cu-BCN-5 monolith measured at 298 K. **b)** $CO_2$ adsorption isotherm showing the average uptake with standard deviation from three independent Cu-BCN-5 monolith at 298 K. The equilibration time at each pressure step was 200 s.

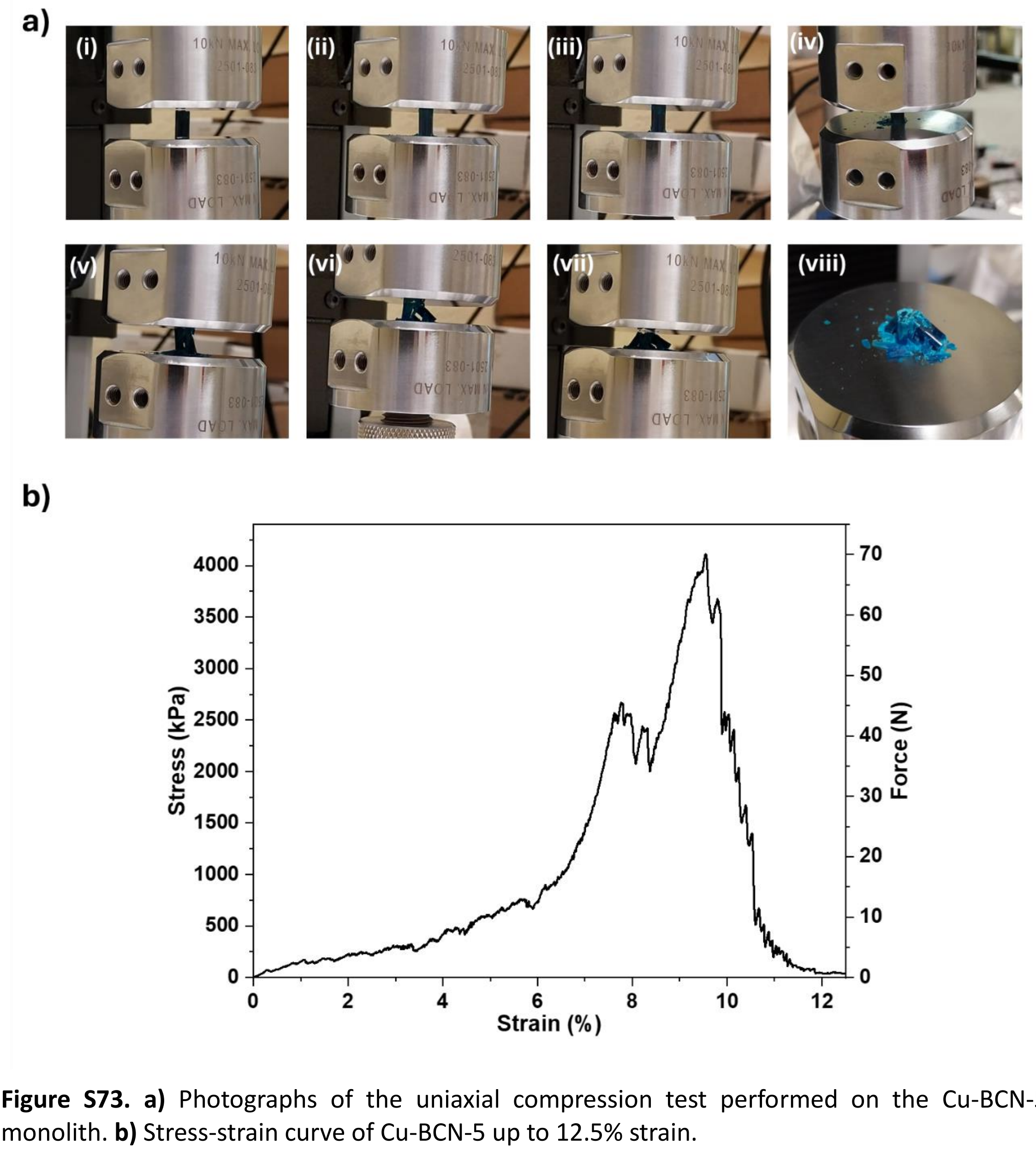


**Figure S73. a)** Photographs of the uniaxial compression test performed on the Cu-BCN-5 monolith. **b)** Stress-strain curve of Cu-BCN-5 up to 12.5% strain.

## S4. References


[1] P. Li, J. A. Schott, J. Zhang, et al., "Electrostatic-Assisted Liquefaction of Porous Carbons," *Angewandte Chemie International Edition* 2017, 56 (47), 14958–14962, https://doi.org/10.1002/anie.201708843.

[2] H. Jin, Y. Liu, Z. Xing, Y. Li, and L. Sun, "Light-Controlled Exposure/Blockage of Permanent Cavities in Metal-Organic Cages-Based Type II Porous Liquids," *Small* 2026, 22 (22), https://doi.org/10.1002/smll.202513827.

[3] M. K. Dinker, K. Zhao, S. Liu, et al., "Permanent Cavities in Ionic Liquids Created by Metal–Organic Polyhedra," *Journal of Materials Chemistry A* 2022, 10 (30), 16204–16211, https://doi.org/10.1039/D2TA02860C.

[4] Y. Xin, D. Wang, W. Zhang, et al., "Dissolution of Metal Organic Cage in Deep Eutectic Solvent: A Solution for Preparing Type II Porous Liquids," *Industrial & Engineering Chemistry Research* 2025, 64 (13), 7044–7055, https://doi.org/10.1021/acs.iecr.5c00183.

[5] Z. Zhang, H. Ning, H. Liu, et al., "Synthesis of Type II Porous Liquids from Nonporous Metal–Organic Polyhedra Enhanced by Host–Guest Complexation*," Angewandte Chemie International Edition* 2025, 64 (50), e202519288, https://doi.org/10.1002/anie.202519288.

[6] Y. Liu, B. Xue, and P. Yin, "Microscopic Mechanism of Gas Sorption in Type II Porous Liquid of Metal–Organic Polyhedra," *The Journal of Physical Chemistry Letters* 2026, 17 (15), 4497–4504, https://doi.org/10.1021/acs.jpclett.6c00811.

[7] Y. Liu, H.-Y. Jin, M.-M. Li, et al., "From the Same Supramolecular Framework to Distinct Types of Porous Liquids via In-Situ Transformation," *Nature Communications* 2026, 17 (1), 3072, https://doi.org/10.1038/s41467-026-69837-8.

[8] M. K. Dinker, M. Li, K. Zhao, et al., "Transformation of Type III to Type II Porous Liquids by Tuning Surface Rigidity of Rhodium(II)-Based Metal-Organic Polyhedra for CO2 Cycloaddition," *Angewandte Chemie International Edition* 2023, 62 (31), e202306495, https://doi.org/10.1002/anie.202306495.

[9] Y. Xu, Y. Ren, G. Zhou, et al., "Amide-Engineered Metal–Organic Porous Liquids Toward Enhanced $CO_2$ Photoreduction Performance," *Advanced Functional Materials* 2024, 34 (19), 2313695, https://doi.org/10.1002/adfm.202313695.

[10] M.-M. Li, M. K. Dinker, Y. Liu, et al., "Role of Cavities Created by Azobenzene-Modified UiO-66 in Bulky Ionic Liquid for High Photoresponsive $CO_2$ Uptake Behavior," *Journal of Materials Chemistry A* 2023, 11 (39), 21058–21065, https://doi.org/10.1039/D3TA04786E.

[11] X. Li, D. Wang, Z. He, et al., "Zeolitic Imidazolate Frameworks-Based Porous Liquids with Low Viscosity for CO2 and Toluene Uptakes," *Chemical Engineering Journal* 2021, 417, 129239, https://doi.org/10.1016/j.cej.2021.129239.

[12] S. He, L. Chen, J. Cui, et al., "General Way To Construct Micro- and Mesoporous Metal–Organic Framework-Based Porous Liquids," *Journal of the American Chemical Society* 2019, 141 (50), 19708–19714, https://doi.org/10.1021/jacs.9b08458.

[13] J. Avila, L. F. Lepre, C. C. Santini, et al., "High-Performance Porous Ionic Liquids for Low-Pressure CO2 Capture," *Angewandte Chemie International Edition* 2021, 60 (23), 12876–12882, https://doi.org/10.1002/anie.202100090.

[14] J. Cahir, M. Y. Tsang, B. Lai, et al., “Type 3 Porous Liquids Based on Non-Ionic Liquid Phases – a Broad and Tailorable Platform of Selective, Fluid Gas Sorbents,” *Chemical Science* 2020, 11 (8), 2077–2084, https://doi.org/10.1039/C9SC05770F.

[15] P.-C. Han, C.-H. Chuang, S.-W. Lin, et al., “Phase-Transformable Metal-Organic Polyhedra for Membrane Processing and Switchable Gas Separation,” *Nature Communications* 2024, 15 (1), 9523, https://doi.org/10.1038/s41467-024-53560-3.

[16] P. Li, J. A. Schott, J. Zhang, et al., “Electrostatic-Assisted Liquefaction of Porous Carbons,” *Angewandte Chemie International Edition* 2017, 56 (47), 14958–14962, https://doi.org/10.1002/anie.201708843.

[17] D. Wang, Y. Xin, W. Fan, et al., “Type I Porous Liquids with Super-Low Viscosities: The Construction through a Rather Simple One-Step Covalent Linkage Strategy and Its Facile Regulation by a Mixed-Ligand Strategy,” *Small* 2025, 21 (4), 2408466, https://doi.org/10.1002/smll.202408466.

[18] X. Xiang, Z. Wang, M. Yamashita, et al., “Viscosity and Pore Accessibility Control of Type I Porous Liquids by Modifying Surface Functionality of Metal-Organic Polyhedra,” Viscosity and Pore Accessibility Control of Type I Porous Liquids by Modifying Surface Functionality of Metal-Organic Polyhedra, 2026, https://doi.org/10.26434/chemrxiv.15001444/v1.

[19] C. von Baeckmann, J. Martínez-Esaín, J. A. Suárez del Pino, et al., “Porous and Meltable Metal–Organic Polyhedra for the Generation and Shaping of Porous Mixed-Matrix Composites,” *Journal of the American Chemical Society* 2024, 146 (11), 7159–7164, https://doi.org/10.1021/jacs.4c00407.

[20] C. He, Y.-H. Zou, D.-H. Si, et al., “A Porous Metal-Organic Cage Liquid for Sustainable CO2 Conversion Reactions,” *Nature Communications* 2023, 14 (1), 3317, https://doi.org/10.1038/s41467-023-39089-x.

[21] L. Tan, K. Zheng, J.-H. Zhou, et al., “Direct Liquefying Organic Cages into Porous Liquid Molecules for Enhanced Near-Infrared Photothermal Conversion and Catalysis,” *Nature Communications* 2025, 16 (1), 8033, https://doi.org/10.1038/s41467-025-63126-6.

[22] K. Jie, N. Onishi, J. A. Schott, et al., “Transforming Porous Organic Cages into Porous Ionic Liquids via a Supramolecular Complexation Strategy,” *Angewandte Chemie International Edition* 2020, 59 (6), 2268–2272, https://doi.org/10.1002/anie.201912068.

[23] M. C. Brand, F. Greenwell, R. Clowes, et al., “Melt-Quenched Porous Organic Cage Glasses,” *Journal of Materials Chemistry A* 2021, 9 (35), 19807–19816, https://doi.org/10.1039/D1TA01906F.

[24] C. Zhou, L. Longley, A. Krajnc, et al., “Metal-Organic Framework Glasses with Permanent Accessible Porosity,” *Nature Communications* 2018, 9 (1), 5042, https://doi.org/10.1038/s41467-018-07532-z.

[25] L. Frentzel-Beyme, M. Kloß, R. Pallach, et al., “Porous Purple Glass – a Cobalt Imidazolate Glass with Accessible Porosity from a Meltable Cobalt Imidazolate Framework,” *Journal of Materials Chemistry* A 2019, 7 (3), 985–990, https://doi.org/10.1039/C8TA08016J.

[26] Y. Wang, H. Jin, Q. Ma, et al., "A MOF Glass Membrane for Gas Separation," *Angewandte Chemie International Edition* 2020, 59 (11), 4365–4369, https://doi.org/10.1002/anie.201915807.

[27] A. M. Bumstead, C. Castillo-Blas, I. Pakamorė, et al., "Formation of a Meltable Purinate Metal–Organic Framework and Its Glass Analogue," *Chemical Communications* 2023, 59 (6), 732–735, https://doi.org/10.1039/D2CC05314D.

[28] H. Xia, H. Jin, Y. Zhang, et al., "A Long-Lasting TIF-4 MOF Glass Membrane for Selective CO2 Separation," *Journal of Membrane Science* 2022, 655, 120611, https://doi.org/10.1016/j.memsci.2022.120611.

[29] S. Li, C. Ma, J. Hou, et al., "Highly Porous Metal-Organic Framework Glass Design and Application for Gas Separation Membranes," *Nature Communications* 2025, 16 (1), 1622, https://doi.org/10.1038/s41467-025-56295-x.

[30] L. Frentzel-Beyme, P. Kolodzeiski, J.-B. Weiß, A. Schneemann, and S. Henke, "Quantification of Gas-Accessible Microporosity in Metal-Organic Framework Glasses," *Nature Communications* 2022, 13 (1), 7750, https://doi.org/10.1038/s41467-022-35372-5.
